# A New Class of Vitamin K Analogues Containing the Side Chain of Retinoic Acid Have Enhanced Activity for Inducing Neuronal Differentiation

**DOI:** 10.1021/acschemneuro.5c00111

**Published:** 2025-07-03

**Authors:** Yoshihisa Hirota, Taiki Sato, Rina Watanabe, Kazuki Takeda, Sho Sano, Satoshi Asano, Yuki Shibahashi, Yumi Yasuda, Yuta Takagi, Yutaro Yamashita, Wu YuXin, Mikino Arakawa, Yuri Maitani, Vannessa Lawai, Kurumi Nakagawa, Natsuko Furukawa, Atsuko Takeuchi, Chisato Tode, Maya Kamao, Akimori Wada, Zainab Ngaini, Yoshitomo Suhara

**Affiliations:** † Laboratory of Biochemistry, Department of Bioscience and Engineering, College of Systems Engineering and Science, 496995 Shibaura Institute of Technology, 307 Fukasaku, Minuma-ku, Saitama 337-8570, Japan; ‡ Laboratory of Organic Synthesis and Medicinal Chemistry, Department of Bioscience and Engineering, College of Systems Engineering and Science, Shibaura Institute of Technology, 307 Fukasaku, Minuma-ku, Saitama 337-8570, Japan; § Laboratory of Toxicology, School of Veterinary Medicine, 12877 Kitasato University, E23-35-1, Towada, Aomori 034-0021, Japan; ∥ Department of Computer Science, Tokyo Institute of Technology, 4259-J3-1818, Nagatsuta-cho, Midori-ku, Yokohama-shi, Kanagawa 226-0026, Japan; ⊥ Faculty of Resource Science and Technology, 54706 Universiti Malaysia Sarawak, Kota Samarahan 94300 Sarawak, Malaysia; # Instrumental Analysis Center, 12883 Kobe Pharmaceutical University, 4-19-1 Motoyamakita-machi, Higashinada-ku, Kobe 658-8558, Japan; ¶ Extension Center, Kobe Pharmaceutical University, 4-19-1 Motoyamakita-machi, Higashinada-ku, Kobe 658-8558, Japan; ∇ Department of Life Science for Organic Chemistry, Kobe Pharmaceutical University, 4-19-1 Motoyamakita-machi, Higashinada-ku, Kobe 658-8558, Japan

**Keywords:** vitamin K, retinoic acid, neuronal differentiation, nuclear receptor, pharmacokinetics, biological metabolism

## Abstract

Vitamin K, primarily known for its roles in coagulation and bone metabolism, has recently been implicated in neuroprotection and neuronal differentiation, particularly via its bioactive form, menaquinone-4 (MK-4). Here, we synthesized 12 vitamin K compounds with retinoic acid-conjugated side chains and methyl ester modifications to enhance neuroactive properties. Among these, compound **7** demonstrated superior stability, robust transcriptional activation via steroid and xenobiotic receptor and retinoic acid receptor, and efficient induction of neuronal differentiation in mouse neural progenitor cells. Mechanistic analyzes revealed that Vitamin K activates metabotropic glutamate receptor 1 (mGluR1). Docking simulations confirmed its stronger mGluR1-binding affinity compared to MK-4. In vivo pharmacokinetics in C57BL/6 mice showed effective blood–brain barrier penetration, with compound **7** metabolizing into MK-4 over time. These findings establish compound **7** as a promising candidate for neurodegenerative disease therapies through its unique neuroactive mechanisms.

## Graphical abstract

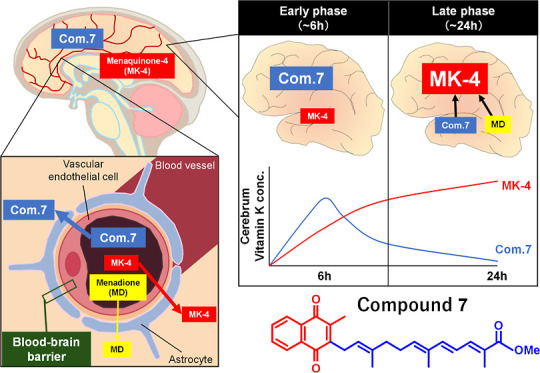

## Introduction

Vitamin K is a fat-soluble vitamin composed of a naphthoquinone ring and a side chain structure. The natural vitamin K homologues are vitamin K_1_ (phylloquinone), which has a phytyl group as its side chain, and vitamin K_2_ (menaquinone-*n*; MK-*n*, *n* = 1–14), whose side chain comprises multiple isoprenyl groups.^1^ Vitamin K_3_ (menadione; MD) lacks a side chain and is a synthetic vitamin K (Figure 1).^2^

**Figure 1:**
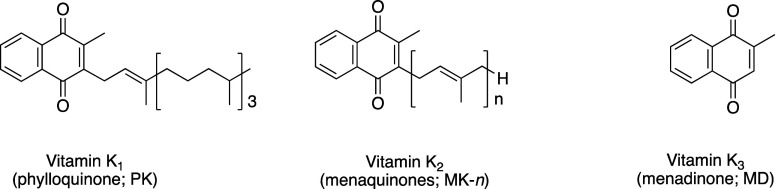
Chemical structures of vitamin K homologues.

The main physiological action of vitamin K is its coenzyme activity with γ-glutamyl carboxylase, which is involved in blood coagulation and bone formation.^3^ Thus, vitamin K was once used in clinical practice to prevent bleeding in newborns and as a therapeutic drug for osteoporosis, although alternative therapies are generally used today. In recent years, several additional actions of vitamin K have been identified. For example, it is an agonist of a nuclear receptor, steroid and xenobiotic receptor (SXR), through which it enhances signal pathway related to bone formation.^4^ Vitamin K is recently reported to be a potent inhibitor of ferroptosis, a form of cell death.^5–7^ Vitamin K also protects nerves from oxidative stress^8^ and can repair damaged neurons via the vitamin K-dependent protein Gas6, which is activated by γ-glutamyl carboxylase.^9^

Recently, we found that one of the vitamin K_2_ homologues, MK-4, can induce the differentiation of neural progenitor cells to neurons.^10^ Neural progenitor cells, which are located in several regions of the adult human brain and contribute to neurogenesis, are capable of differentiating into neurons, astrocytes, and oligodendrocytes.^11^ Our finding that MK-4 can induce neuronal differentiation suggests a potential means of producing new neurons to alleviate the decrease of neurons that is characteristic of several neurodegenerative diseases such as Alzheimer’s, Parkinson’s, Huntington’s, and prion disease.^12^ Alzheimer’s is the most common neurological disease, accounting for about half of all dementia diagnoses worldwide, and this disease has become a major social problem because it significantly impairs memory, communication, and thinking, which makes round-the-clock nursing care essential.^13^ Although there are drugs available to alleviate symptoms of Alzheimer’s, there is currently no cure. If it were possible to induce differentiation of endogenous neural stem cells into neurons, it may be possible to restore the brain to normalcy by replacing the neurons that have been depleted by disease, thereby delaying the onset of disease or even reversing symptoms. Although some naturally occurring compounds with such activity have been reported,^14,15^ they do not exhibit sufficient activity for application as a regenerative medicine. To address this lack of activity, in previous studies we have introduced hydrophobic functional groups such as a phenyl group at the ω-terminal position of the side chain of several of the vitamin K_2_ homologues, resulting in the identification of several compounds with higher activity than the natural vitamin K homologues.^10,16,17^

Another naturally occurring compound with neuronal differentiation-inducing activity is retinoic acid, an active metabolite of vitamin A that acts as a signaling molecule for retinoic acid receptor (RAR), a nuclear receptor that is involved in various physiological activities such as cell proliferation and differentiation.^18,19^ Retinoic acid is reported to inhibit neural stem cell self-renewal and promote neuronal differentiation.^15^ Since it has been reported that the degree to which vitamin K homologues induce neuronal differentiation differs depending on their side chain structure,^10^ we hypothesized that the side chain is also important for the differentiation-inducing activity of retinoic acid. The side chain of retinoic acid has a conjugated structure and a terminal carboxy group. We anticipated that if those characteristic structures were introduced to the side chain of vitamin K, the differentiation activity of the resulting vitamin K analogues would be increased.

In the present study, we designed and synthesized several hybrid homologues and examined their activities. We began by synthesizing compounds **1** and **2**, originally reported by Fujii et al.,^20^ in which a carboxylic acid moiety was introduced at the ω-terminal side chain of vitamin K_2_ (Figure 2A). We also synthesized similar compounds **3** and **4** in which a methyl ester was introduced at the ω-terminal side chain. We then evaluated the neuronal differentiation activity of these compounds by quantifying the change in mRNA expression of *Map2*, a surface antigen expressed by neurons, induced by these compounds in neural progenitor cells by reverse transcription polymerase chain reaction (RT-PCR). We found that the methyl ester homologues had more potent differentiation-inducing activity than the carboxylic acid homologues, although their activities were still lower than those of the natural vitamin K homologues MK-2 to -4 (Figure 2B). Based on these findings, we predicted that vitamin K analogues containing methyl ester moieties would have more potent activity than homologues containing carboxylic acid moieties; however, it has been reported that the carboxylic acid form of vitamin K produced by ω-oxidation cannot exist stably in the body for a long time and is catabolically metabolized to K acids and excreted by sequential cleavage of the side chain upon β-oxidation.^20^

**Figure 2:**
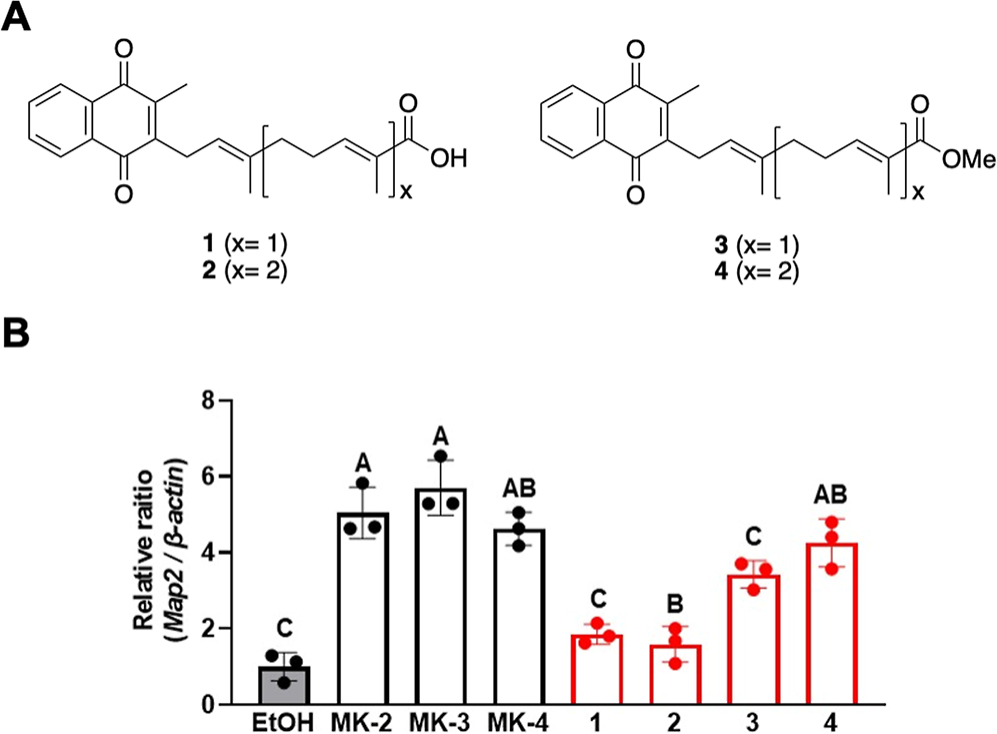
(A) Structures of vitamin K analogues into which a carboxylic acid (**1**, **2**) or methyl ester (**3**, **4**) moiety was introduced at the ω-terminal side chain. (B) Induction of neuronal differentiation in neural progenitor cells by three natural vitamin K homologues (MK-2, MK-3, and MK-4) and four synthetic vitamin K analogues (**1**–**4**). Neural progenitor cells were isolated from embryonic mouse cerebrum, treated with 1 μM of the vitamin K compounds, and then the intracellular *Map2* mRNA levels were determined. Values not sharing a common letter in each group are significantly different (*n* = 3); *p* < 0.01 (Tukey–Kramer HSD test).

Next, we synthesized five additional vitamin K homologues in which the conjugated side chain motif of retinoic acid was added to the naphthoquinone ring of vitamin K. Compounds **5** and **6** were synthesized by adding the conjugated structural motif to the side chain of compounds **3** and **4**, respectively. Based on our earlier findings obtained for compounds **1**–**4** (Figure 3), compounds **7** and **8** were synthesized by changing the moiety at the end of the side chain to a methyl ester. Finally, compound **9** was synthesized by adding the whole side chain of retinoic acid, also capped with a methyl ester moiety, to the naphthoquinone ring of vitamin K. The structure of the side chain of compound **9** differs from that of the other compounds. Compounds **5**–**8** have a double bond between C8′–C9′ or C12′–C13′ of the side chain of vitamin K, forming a conjugated structure between C6′–C11′ or C10′–C15′, whereas compound **9** has a conjugated structure between C3′ and C8′, and has the same structure as part of the side chain of ATRA (Figure 3). To determine the degree to which these compounds retained the functional properties of retinoic acid and vitamin K, we examined their ability to induce neuronal differentiation, as well as their transcriptional activity via the nuclear receptors SXR and RAR, which are the target proteins of vitamin K and retinoic acid, respectively. Finally, we also conducted an in vivo pharmacokinetics study in mice to examine the stability and effects of compound **7**, the compound that showed the highest transcription- and neuronal differentiation-inducing activities.

**Figure 3:**
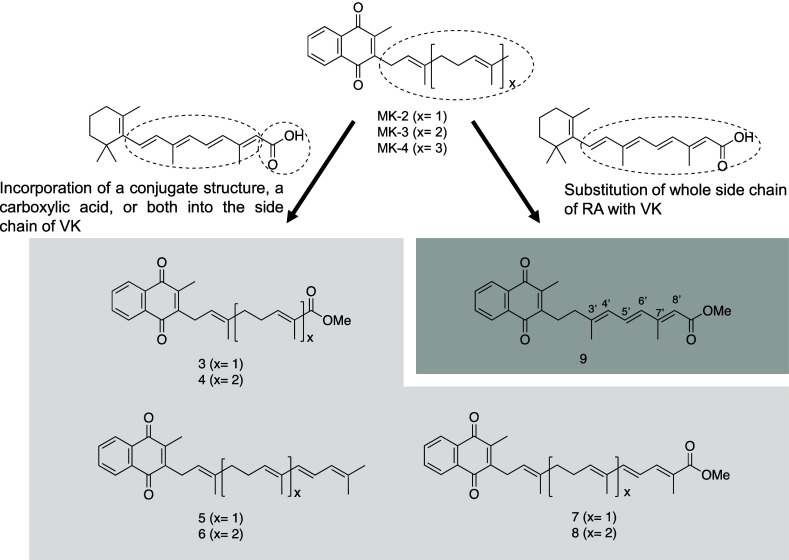
Structures of novel vitamin K analogues **3–9**. Analogues **3–8** in which either or both of the conjugated structure of retinoic acid and a carboxylic acid cap were introduced to the side chain of vitamin K. Analogue **9** contained the whole side chain of RA capped with a methyl ester.

## Results and Discussion

### Molecular Design and Synthesis

Scheme 1 shows the synthetic pathway used to prepare compounds **1**–**8**. First, we synthesized **1**–**4**, in which the side chain of vitamin K was converted to a carboxylic acid or methyl ester. These compounds were obtained by first converting MK-2 and MK-3 to aldehydes **10** and **11** in two steps, and then subjecting the terminal aldehydes to Pinnick oxidation to afford **1** and **2** in 48% and 58% yield, respectively; the detailed methods can be found in a previous report.^20^ Then, the carboxylic acid moieties of **1** and **2** were converted to methyl esters by using diazomethane to afford **3** and **4** in good yields (85%–90%).

**Scheme 1:**
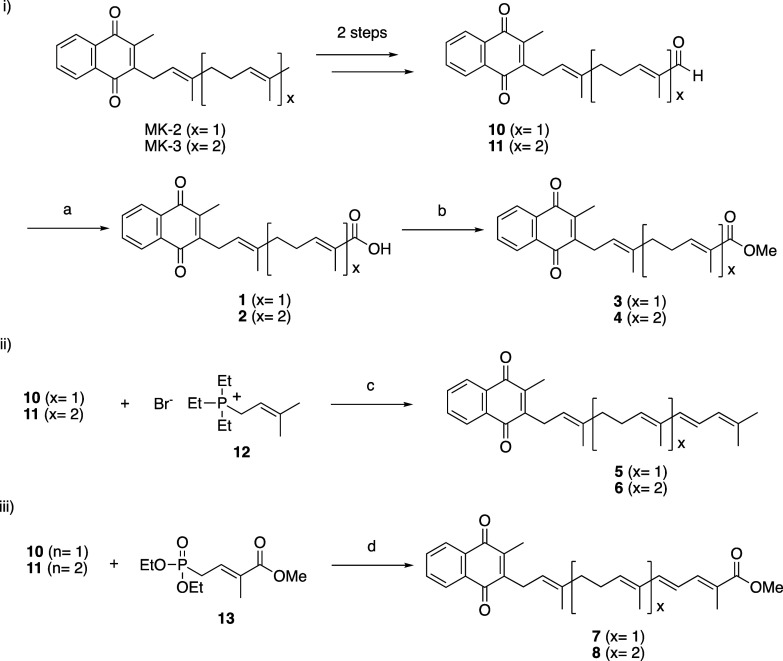
Synthesis of Vitamin K Analogues **1**–**8**

Reagents and conditions: (a) 2-methyl-2-butene, NaH_2_PO_4_, sodium chlorite, THF/H_2_O (3:1), r.t., 2 h, 48%–58%; (b) CH_2_N_2_, ether, 0 °C to r.t., 1 h, 85%–90%; (c) *n*-BuLi, THF, −78 °C, 3 h, 30%; (d) *n*-BuLi, THF, 0 °C to r.t., 3 h, 37%–40%.

For the synthesis of analogues **5** and **6**, in which only the conjugated structural motif of retinoic acid was introduced to the side chain of vitamin K, and the synthesis of analogues **7** and **8**, in which both the conjugated structural motif and a carboxy group were incorporated, we first attempted to use a vitamin K derivative in which the naphthoquinone structure was converted to 1,4-dimethoxy-2-methylnaphthalene. We did this because the Wittig or Horner–Wadsworth–Emmons reagent needed to construct the conjugated structure via formation of double bonds was expected to react with the quinone part of the naphthoquinone and reduce the reaction yield. Although we succeeded in introducing the conjugated structure by the Wittig reaction and obtained **5**–**8**, the conjugated structure decomposed when the methyl group of 1,4-dimethoxy-2-methylnaphthalene was deprotected with ceric ammonium nitrate in the final step. Presumably, the conjugated structure could not bear the acidic conditions. Therefore, we synthesized **5**–**8** without protecting the quinone moiety and accepted the reduced yield. As with the synthesis of **1**–**4**, aldehydes **10** and **11** were also used as intermediates for the synthesis of **5**–**8**. For the synthesis of **5** and **6**, we first attempted to use diethylphosphono reagent as a Horner–Wadsworth–Emmons reagent, but this was not successful. It is probably because the reactivity of the stable phosphonate anion generated from the reagent was low. Instead, we used triethyl phosphite **12** as a Wittig reagent, which was obtained from the reaction of bromide **14** with triethyl phosphite (Scheme 2). Thus, aldehydes **10** and **11** were treated with Wittig reagent **12** to afford trans-isomers **5** and **6**, each in 30% yield. Compounds **7** and **8** were synthesized from **10** and **11** with Horner–Wadsworth–Emmons reagent **13**, which was obtained via the Arbuzov reaction^17^ of bromide **15** with triethyl phosphite (Scheme 2); the yields of **7** and **8** were 37% and 40%, respectively.

**Scheme 2:**
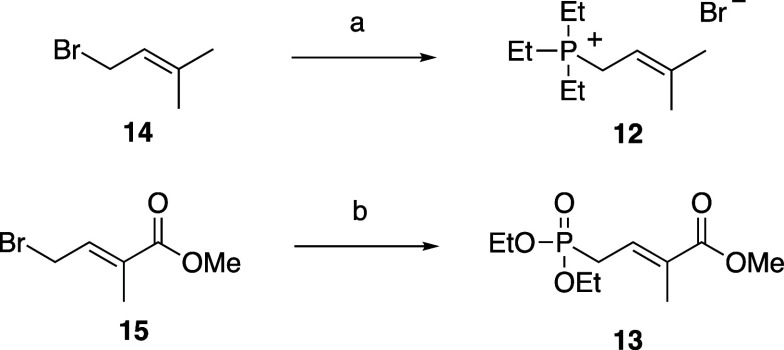
Synthesis of Wittig Reagent **12** and Horner–Wadsworth–Emmons Reagent **13** ^a^

a Reagents and conditions: (a) triethyl phosphine, toluene, 120 °C, 3 h, 96%; (b) triethyl phosphite, toluene, 120 °C, 5 h, 70%.

Scheme 3 shows the synthesis of analogue **9**, in which the vitamin K side chain was replaced with the whole retinoic acid side chain capped with a methyl ester moiety. To begin, compound **17** was derived from menadione and levulinic acid (**16**) in 51% yield. Next, the ketone group of the side chain of **17** was converted to an acetal to afford **18**, and the naphthoquinone ring of **18** was reduced and methylated to afford **19**. After deprotection of the acetal group by acid hydrolysis to obtain ketone **20**, the side chain was elongated by Horner–Wadsworth–Emmons reaction to obtain methyl ester **21** in 68% yield. Reduction of **21** with lithium aluminum hydride led to **22** with an alcohol moiety at the terminal position of the side chain, and then deprotection of the methyl group using ceric ammonium nitrate gave **23**. Next, oxidation of **23** with MnO_2_ gave aldehyde **24** in 78% yield. Finally, compound **9** was produced in 63% yield by the formation of the conjugated structural motif by Horner–Wadsworth–Emmons reaction using **25**.

**Scheme 3:**
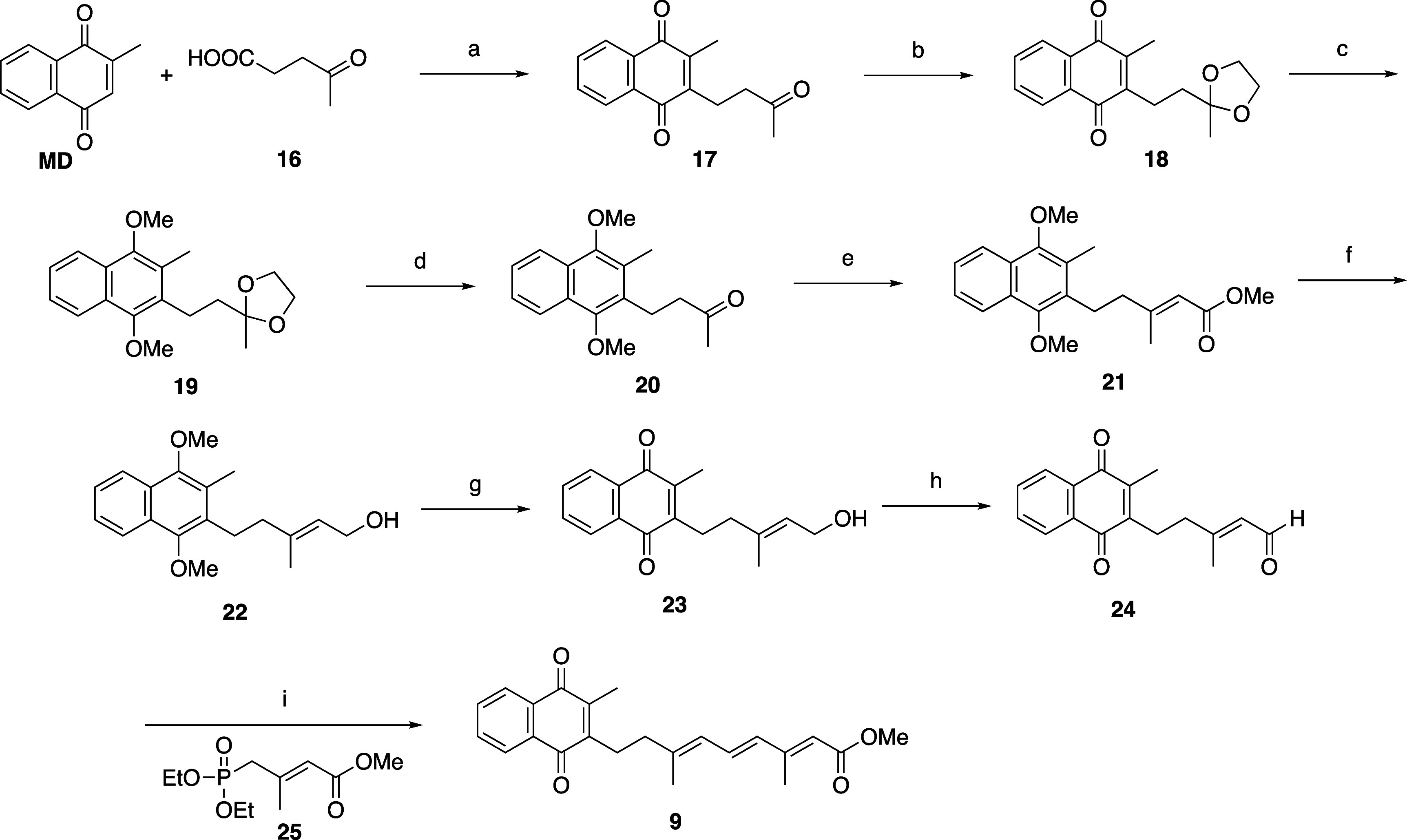
Synthesis of Vitamin K Analogue **9** ^a^

a Reagents and conditions: (a) ammonium peroxodisulfate, silver nitrate, CH_3_CN, H_2_O, 70 °C, 2 h, 51%; (b) ethylene glycol, H_2_SO_4_, benzene, 90 °C, 2 h, 97%; (c) TBABr, sat. Na_2_S_2_O_4_ aq., THF, 0 °C to r.t., 24 h, 67%; (d) 1 M HCl aq., THF, r.t., 19 h, 98%; (e) trimethylphosphonoacetate, NaH, THF, 0 to 65 °C, 24 h, 68%; (f) LAH, THF, 0 °C to r.t., 24 h, 94%; (g) ceric ammonium nitrate, H_2_O, CH_3_CN, r.t., 40 min, 82%; (h) MnO_2_, CH_2_Cl_2_, r.t., 5 h, 78%; (i) *n*-BuLi, THF, r.t., 24 h, 63%.

Horner–Wadsworth–Emmons reagent **25** was prepared from methyl-3,3-dimethyl acrylate (**26**) according to a reported method (Scheme 4).^21^ In short, the allyl position of **26** was brominated and converted to **27** using *N*-bromosuccinimide. Then, **27** and triethyl phosphite were heated and refluxed to obtain **25** with a yield of 47%.

**Scheme 4:**
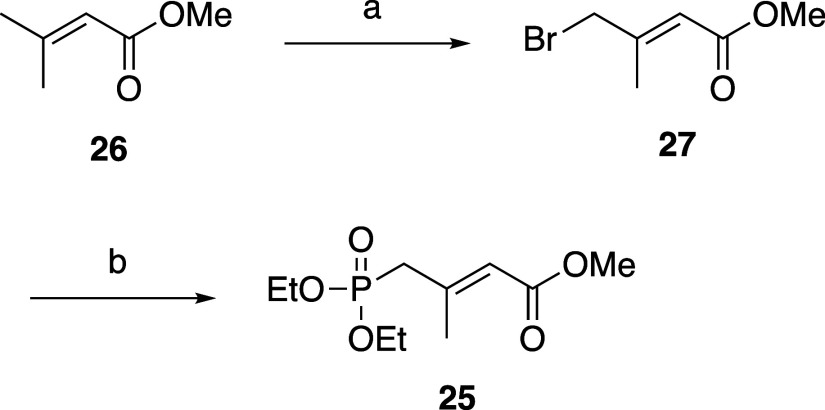
Synthesis of Horner–Wadsworth–Emmons Reagent **25** ^a^

a Reagents and conditions: (a) *N*-bromosuccinimide, CH_2_Cl_2_, 0 to 40 °C, 2 h, 37%; (b) triethyl phosphite, 120 °C, 5 h, 47%.

### Regulation of Transcriptional Activity via SXR and RAR

To evaluate whether the biological activity of vitamin K was preserved in **5–9**, we evaluated their ability to regulate transcription activity via SXR.^22^ In addition, since these analogues included part of the retinoic acid side chain, we also evaluated the ability of the analogues to regulate transcriptional activity via RAR.^23^ Transcriptional activity was assessed by luciferase assay using murine neural progenitor cells and luciferase reporter plasmids containing the binding sequences of SXR and RAR, following the method we reported previously.^24^ When the cells were treated with 1 μM of each of the analogues, significantly higher SXR-mediated transcriptional activity was observed compared not only with control (EtOH treatment) but also with the naturally occurring vitamin K homologues MK-2 to -4 (Figure 4A). In particular, **7** and **8**, which possessed the conjugated structural motif of retinoic acid capped with a carboxylic acid moiety, induced the greatest increases in transcriptional activity. Similarly, treatment with the analogues significantly increased RAR-mediated transcriptional activity compared with control, although the increase did not match that induced by all-trans retinoic acid, which was used as a positive control (Figure 4B). Thus, given that the synthesized analogues increased transcriptional activity via SXR more than did the natural vitamin K homologues, but did not increase transcriptional activity via RAR to the same degree as all-trans retinoic acid, we concluded that the activities of **5–9** are closer to that of vitamin K than to that of retinoic acid.

**Figure 4:**
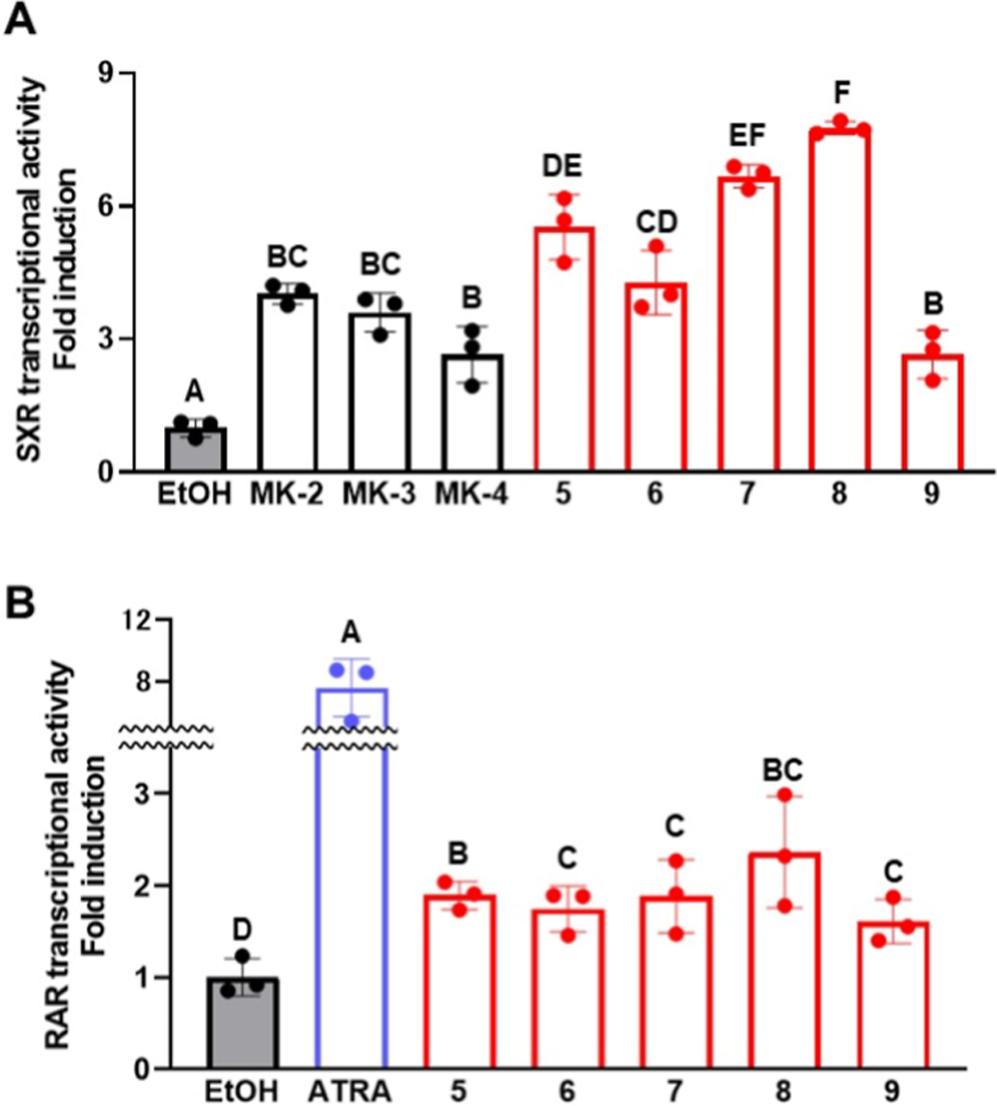
Effects of vitamin K analogues **5**–**9** on steroid and xenobiotic receptor (SXR)- and retinoic acid receptor (RAR)-mediated transcriptional activity in murine neural progenitor cells. Neural progenitor cells were isolated from embryonic mouse cerebrum and then treated with 1 μM (A) or 10 μM (B) of the indicated vitamin K analogues or control compounds. (A) Effects of the natural vitamin K homologues MK-2 to -4, and of the synthesized vitamin K analogues **5**–**9**, on SXR-mediated transcriptional activity. EtOH was used as the negative control. Values not sharing a common letter in each group are significantly different (*n* = 3); *p* < 0.01 (Tukey–Kramer HSD test). (B) Effects of all-*trans* retinoic acid (ATRA; positive control) and analogues **5**–**9** on RAR-mediated transcriptional activity. Values not sharing a common letter in each group are significantly different (*n* = 3); *p* < 0.05 (Tukey–Kramer HSD test).

### Induction of Neuronal Differentiation

The ability of the vitamin K analogues to induce neuronal differentiation was investigated in neural progenitor cells by fluorescent immunostaining and RT-PCR, as we reported previously.^10,11^ First, we used fluorescent immunostaining to examine the differentiation status of neural stem cells treated with each of the compounds and stained for microtubule-associated protein 2 (Map2), a marker of neural growth that is specifically expressed in neurons (Figure 5A). Strong Map2-positive signals were observed in the cells treated with the natural vitamin K homologues MK-2 to -4 and compounds **5**–**8**, but only a weak signal was observed in cells treated with compound **9** (Figure 5A).

**Figure 5:**
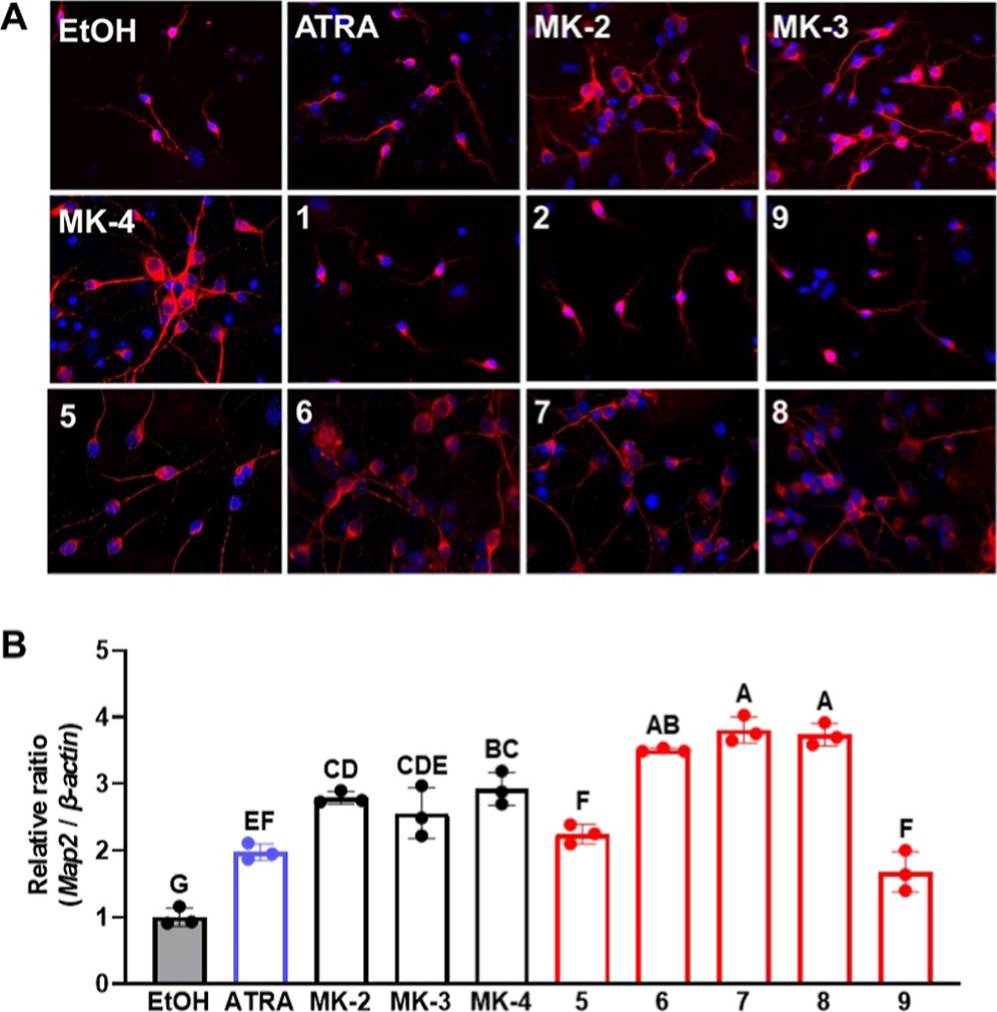
Effects of vitamin K analogues **5**–**9** on neuronal differentiation in murine neural progenitor cells. Neural progenitor cells were isolated from embryonic mice cerebrum and treated with 1 μM of a natural vitamin K homologue (MK-2 to -4) or a vitamin K analogue (**5**–**9**), or with 0.1 μM all-*trans* retinoic acid (ATRA). (A) After 48 h, the cells were immunostained for microtubule-associated protein 2 (Map2; red), a marker of neural growth, and with the nuclei stain DAPI (blue). Map2 localization was considered to indicate the induction of neuronal differentiation. (B) In other neural progenitor cells, also treated for 48 h, the expression of *Map2* mRNA was assessed by real-time polymerase chain reaction. Values not sharing a common letter in each group are significantly different (*n* = 3); *p* < 0.05 (Tukey–Kramer HSD test).

Since the fluorescent immunostaining pictures could not be accurately quantified, we instead used RT-PCR to quantify the mRNA expression level of the *Map2* gene^11^ in neural progenitor cells treated with the analogues (Figure 5B). A significant increase in *Map2* mRNA expression was observed in cells treated with **5**–**8** compared to EtOH-treated cells. For compounds **5** and **6**, which possessed only the conjugated structure of retinoic acid, the activity was greater for the compound with the longest side chain structure (i.e., compound **6**). Compounds **7** and **8**, which possessed both the conjugated structure of retinoic acid and a methyl ester moiety at the end of the side chain, showed about three times higher neuronal differentiation-inducing activity compared with control, and significantly higher activity than the natural vitamin K homologues. In contrast, compound **9**, which possessed a part of retinoic acid side chain and a methyl ester moiety, showed little activity, inducing only a small increase in the mRNA expression level of *Map*2 compared with control. Taken together, these results indicate that both the conjugated structure and the methyl ester of the side chain are important for enhancing the neuronal differentiation-inducing activity of vitamin K analogues.

### Mechanism of Induction of Neuronal Differentiation

MK-4, the most bioactive of the vitamin K homologues, is reported to be present at high concentrations in the brain (approximately 31.8–56.6 pmol/g in rat, 422.7–993.5 pmol/g in mouse, and 6.3 nmol/g in human.^25–27^ ). This suggests that vitamin K may have some as-yet-unidentified role in the brain. Our data presented in the previous section suggest that one of the roles of vitamin K in the brain is to induce neuronal differentiation. We therefore examined the potential mechanism of this activity in detail.

We treated primary cultured murine neurons with MK-4, compound MP3, which promotes the differentiation of neural stem cells into neurons, and compound 23P2, which suppresses the differentiation of neural stem cells into neurons (Figure S1), and then analyzed the transcriptome of 48,441 genes expressed in the cerebrum (Figure S2A,B). Compared to EtOH-treated cells, the expression levels of 3780 genes were significantly altered in the MK-4-treated neural stem cells. Among them, 273 genes were identified as being involved in neuronal differentiation and 110 genes as being involved in MAPK signaling (Figure S2B–D). Focusing on the genes involved in MAPK signaling, we found that MK-4 and MP3, which both promote neuronal differentiation, act upon many of the same variable genes, and 23P2, which suppresses neuronal differentiation, acts upon a very different set of variable genes (Figure S2E). Through this transcriptome analysis, we finally identified metabotropic glutamate receptors (mGluRs) (Figure 6A) as a playing a significant role in mechanism of induction of neuronal differentiation by vitamin K analogues. Since primary cultured cells are very difficult to knock down genes by RNA interference due to low efficiency of siRNA delivery, we evaluated the involvement of mGluRs using inhibitors of mGluRs.

**Figure 6:**
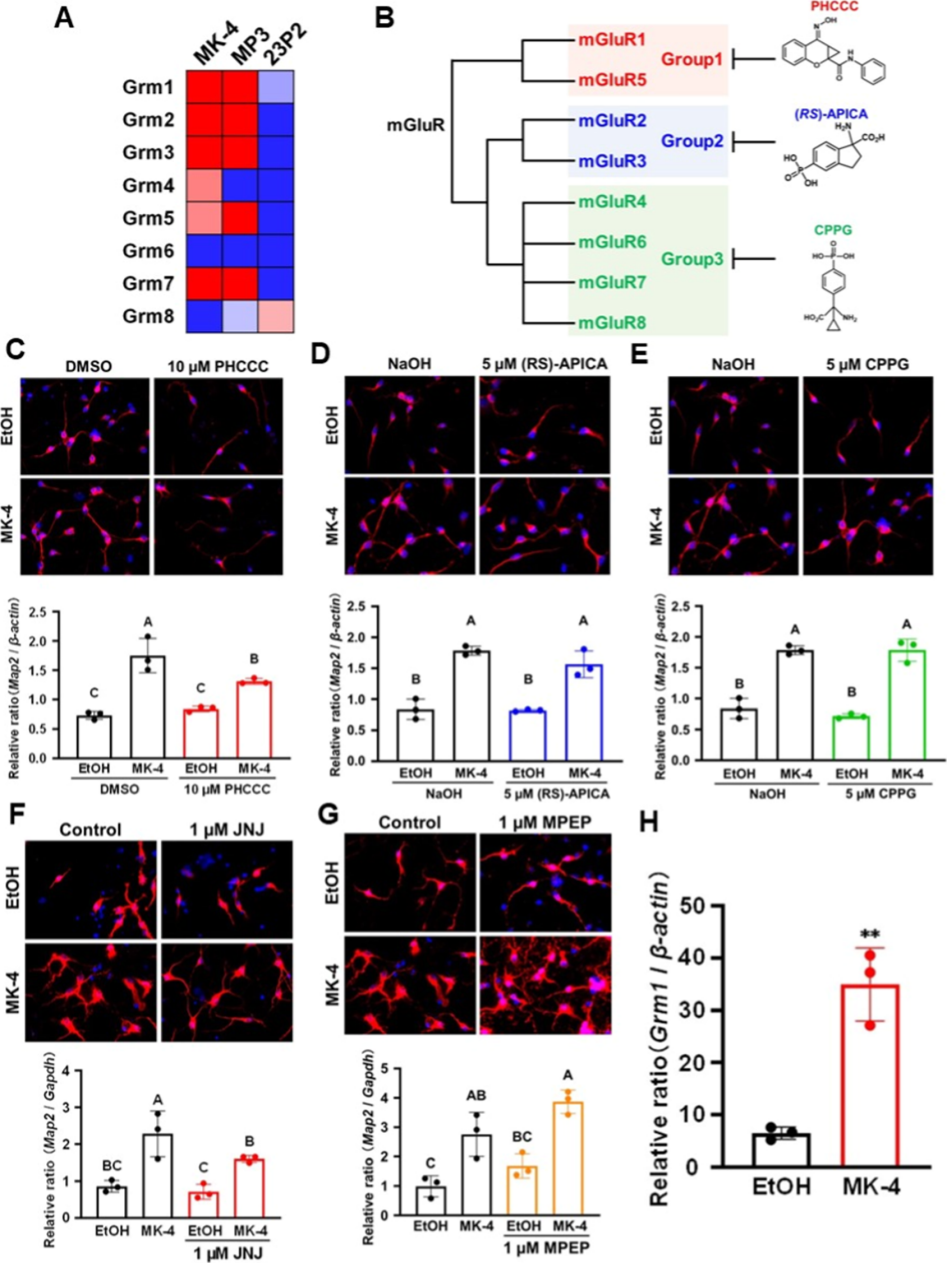
Elucidation of the mechanism underlying the induction of neuronal differentiation by vitamin K analogues. (A) Gene expressions under treatment with MK-4, MP3, or 23P2 as a ratio to gene expression under treatment with EtOH control were calculated as log values and quantitatively compared. Heat maps were produced for genes whose expression levels were decreased by more than half (blue) and increased by more than 2-fold (red) compared to control. (B) Classification of the metabotropic glutamate receptors (mGluRs) into three groups and the structural formulas of three mGluR group-specific inhibitors. (C–G) Neuronal differentiation activity was examined in murine neural stem cells treated with the indicated mGluR inhibitors by fluorescence immunostaining and real-time polymerase chain reaction analysis of *Map2* mRNA expression. JNJ16259685, mGluR1-specific inhibitor; MPEP, mGluR5-specific inhibitor. Values not sharing a common letter in each group were significantly different (*n* = 3); *p* < 0.05 (Tukey–Kramer HSD test). (H) mGluR1 mRNA expression level in MK-4-treated neural stem cells. Significant difference from EtOH; **p < 0.01 (Student’s *t*-test).

Based on existing reports, the mGluRs are classified into three groups: Group 1 includes mGluR1 and mGluR5; Group 2 includes mGluR2 and mGluR3; and Group 3 includes mGluR4, mGluR6, mGluR7, and mGluR8 (Figure 6B).^28^ The receptors in each group are inhibited by different compounds: PHCCC is a Group 1 inhibitor, (*RS*)-APICA is a Group 2 inhibitor, and CPPG is a Group 3 inhibitor (Figure 6B). We evaluated the effects of these inhibitors on the induction of neuronal differentiation by MK-4 in neural stem cells. Cells treated with PHCCC showed significant inhibition of neuronal differentiation (Figure 6C), whereas those treated with (*RS*)-APICA or CPPG showed no difference in neuronal differentiation (Figure 6D, E). Since only the Group 1 inhibitor inhibited neuronal differentiation, we then examined the effects of two additional Group 1 inhibitors: JNJ16259685, a mGluR1-specific inhibitor, and MPEP, a mGluR5-specific inhibitor. MK-4-treated neural stem cells treated with JNJ16259685 showed significantly reduced neuronal differentiation compared with cells not treated with the inhibitor (Figure 6F); however, no change in neuronal differentiation was observed in cells treated with MPEP (Figure 6G). In addition, mRNA expression of the gene encoding mGluR1 (*Grm1*) was significantly increased in MK-4-treated neural stem cells (Figure 6H). Taken together, these findings indicate that vitamin K induces the differentiation of neural stem cells into neurons via mGluR1. However, since the mGluR1-specific inhibitor suppressed MK-4-induced neuronal differentiation by only around 20%, another mechanism must exist.

It is reported that mGluR1 on the plasma membrane induces neural stem cells to differentiate into neurons via signal transduction.^29,30^ In addition, all of the mGluRs except mGluR6 are known to be expressed in neural stem cells in the brain.^31,32^ In particular, mGluR1 has been shown to induce differentiation into neurons in the mouse cerebellum.^33^ mGluR1 has also been shown to be involved in synaptic transmission, and it has been reported that motor dysfunction and neurodegenerative disorders due to synaptic dysfunction occur in mGluR1-knockout mice.^34^ Furthermore, it has been suggested that synaptic dysfunction in old age may contribute to the development of neurodegenerative brain diseases such as Alzheimer’s disease.^35^ Recently, microglia, the immune cells of the brain, have been shown to be involved in synapse formation and removal, and microglial abnormalities have been found to induce synaptic dysfunction, leading to neurodegenerative brain diseases, and mGluR is also expressed in microglia.^35,36^ Together, these findings show that the mGluRs play crucial roles in the brain. If vitamin K activates mGluR-mediated signaling in the brain and induces differentiation into neurons, this activity could potentially be harnessed to improve synaptic dysfunction for the prevention and treatment of neurodegenerative brain diseases such as Alzheimer’s disease.

mGluR1 activation promotes Gαq/11 protein deconjugation and stimulates phospholipase C beta 1, which in turn promotes diacylglycerol and inositol trisphosphate formation, leading to the release of Ca^2+^ from the endoplasmic reticulum; both Ca^2+^ and diacylglycerol have been shown to activate protein kinase C and regulate gene expression.^37^ Besides these signaling pathways, activation of transcription factors and epigenetic controls associated with neuronal differentiation in the nucleus have been reported to induce neuronal differentiation.^38,39^ Indeed, in our present transcriptome analysis we found 27 transcription factors associated with neuronal differentiation in the nucleus (Figure S2C). Among them, the gene expression of chromodomain helicase DNA binding protein 5 (Chd5) was particularly variable, suggesting its involvement in the mechanism by which vitamin K induces neuronal differentiation (Figure S3A,B). Chd5 is highly expressed in neural stem cells, neural progenitor cells, and mature hippocampal neurons in the human, rat, and mouse cerebrum.^40–43^ It is reported that loss of Chd5 in the brain promotes neural stem cell proliferation but inhibits their differentiation into neurons.^44^ This suggests that Chd5 may be involved in the mechanism of induction of neuronal differentiation by vitamin K. Indeed, *Chd5* mRNA expression was markedly increased in MK-4-treated neural stem cells compared to EtOH-treated cells (Figure S3B). We therefore examined the involvement of Chd5 and mGluR1 in the mechanism of induction of neuronal differentiation using JNJ16259685, an inhibitor of mGluR1. Cells treated with the mGluR1 inhibitor showed a significant decrease in Chd5 mRNA levels (Figure S3C).

It has been reported that Chd5 induces neuronal differentiation by directly binding to histones, thereby increasing histone methylation and histone acetylation activity.^41^ Histone methylation and acetylation are mechanisms by which histone methyltransferases and histone acetylases add methyl and acetyl groups to amino acid residues of histone proteins, resulting in changes in chromatin structure and regulation of gene transcription. To examine whether MK-4 activates histone modifications, we assessed protein expression levels of methylated histone (H3Me) and acetylated histone (H3Ac) in MK-4-treated cells. In MK-4-treated cells, although histone H3 methylation was not affected (Figure S3D), expression of acetylated histone H3 was elevated (Figure S3E), suggesting that MK-4 may induce neuronal differentiation by increasing histone acetylation activity. Previously, Hwang et al. reported that in neural stem cells isolated from the cerebrum of embryonic Chd5-deficient mice, the expression of neurons is reduced by decreased acetylation at histone H3 lysine residue 27 (H3K27Ac) .^43^ This suggests that MK-4 may induce neuronal differentiation by increasing the activity of H3K27Ac via Chd5.

Although we did not have Chd5-deficient mice in this study, we may be able to elucidate the mechanism of MK-4-induced neuronal differentiation by using Chd5-deficient mice in the future.

### mGluR1 Docking Simulation Analysis Using Compound **7**

In the previous experiments, we found that mGluR1 was involved in the mechanism of neuronal differentiation induced by the vitamin K analogues. The mGluRs have attracted attention as a therapeutic target for the treatment of neurodegenerative brain diseases.^45,46^ mGluRs are composed of three main domains: an extracellular ligand-binding domain (Venus flytrap domain) to which glutamate binds, a seven-transmembrane domain, and a cysteine-rich domain connecting the two.^47^ Since the mechanism underlying the induction of neuronal differentiation by MK-4 involves mGluR1, we evaluated whether compound **7**, which was the compound with the highest transcription- and neuronal differentiation-inducing activities, binds to mGluR1 as well. A homology analysis of human and mouse mGluR1 revealed 93.5% homology (Figure S4); therefore, although primary cultured mouse cells were used in the previous experiments, here we simulated the docking of both human and mouse mGluR1 for future application to humans.

The interactions of human and mouse mGluR1 with MK-4 and compound **7** are shown (Figures 7A, S5 and S6). The protein structure of mGluR1 indicates the presence of a central cavity, where MK-4 and other vitamin K analogues bind. Docking simulation revealed that the naphthoquinone ring of vitamin K is inserted at the back of the cavity and the side chain is located on the outside (Figure 7A, S5 and S6). Sitemap analysis indicated a predominantly hydrophilic binding pocket (mostly green in these figures), yet both compound **7** and MK-4 bound in similar conformations, as determined by induced-fit docking (Figure 7A). The binding poses for the other vitamin K analogues and human and mouse mGluR1 are shown in the Supporting Information (Figures S5 and S6). Molecular dynamics simulations revealed frequent π–π stacking interactions between the naphthoquinone ring of MK-4 and His55 and Trp110, as well as interactions between the carbonyl groups of MK-4 and Gln56 and Arg323 (Figure 7B). Arg323 also interacted with the terminal carbonyl oxygen of compound **7**. A temporal binding frequency analysis (from 0.00 to 50.00 ns) of MK-4, compound **7**, MP3, and 23P2 with binding residues His55, Trp110, and Arg323 (Figure 7C and S5) revealed that 23P2 had ceased interacting with the residues midway through the simulation and had exited the binding pocket by 30.00 ns (Figure 7C, Figures S5, S6 and Supplementary Video). In contrast, MK4, compound **7**, and MP3 remained bound to His55 and Trp110 out to 50.00 ns. An interesting result was that MK4 had stopped interacting with Arg323 at 23.00 ns, whereas compound **7** and MP3 remained bound to the residue at 50.00 ns (Figures 7C and S5). Since both compound **7** and MP3 exhibited greater activity to induce neuronal differentiation compared with MK-4, binding to Arg323 may underly this high mGluR1 agonist activity. For the development of more potent analogues, it may be important to synthesize compounds that bind more strongly to Arg323 while also binding to His55 and Trp110.

**Figure 7:**
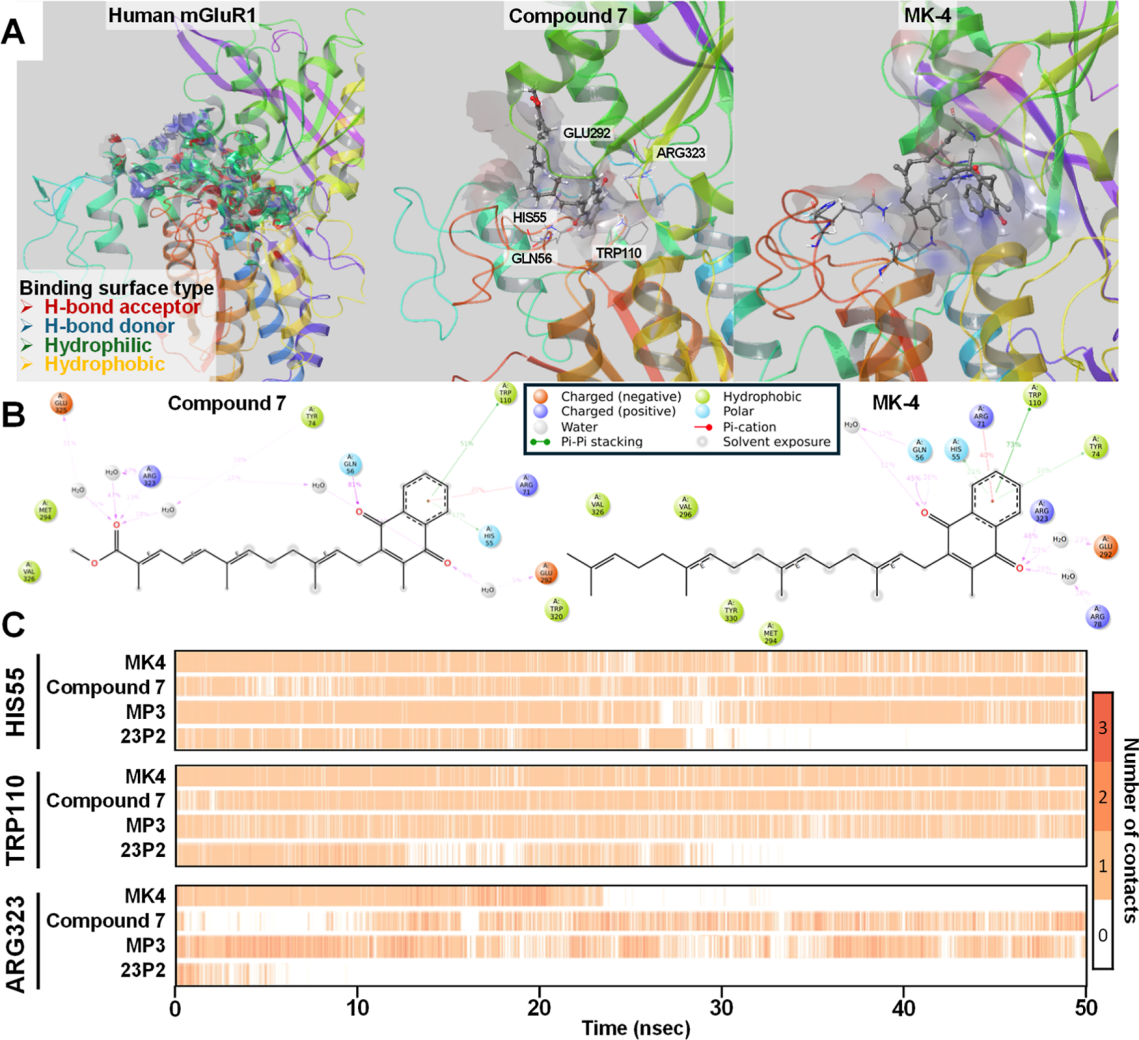
Structural analysis of the binding of human mGluR1 with the indicated vitamin K analogues. (A) Molecular docking analysis of the agonist binding site of mGluR1 and the binding poses of compound **7** and MK-4. Binding surfaces are shown with an electrostatic potential overlay (blue, positive; red, negative). (B) Schematic diagrams of the interactions between ligand atoms and protein residues. Interactions that occurred for more than 5.0% of the simulation time from 0.00 to 50.00 ns are shown. (C) Timeline of the interactions between three representative binding residues (His55, Trp110, and Arg323) and four vitamin K analogues (MK-4, compound **7**, MP3, and 23P2). Some residues make more than one specific contact with the ligand, which is represented by a darker shade of orange, according to the scale to the right.

Compound **7** is expected to undergo hydrolysis of its methyl ester moiety by endogenous esterases, yielding a carboxylic acid-type vitamin K analog (compound **7′**). Since compound **7′** is highly unstable and predicted to degrade rapidly after synthesis, biological assays were considered impractical. Therefore, molecular docking analysis was performed using human mGluR1. When comparing the docking scores of compound **7** and compound **7′**, the latter exhibited a binding affinity more than 1.0 kcal/mol stronger than that of compound **7**—corresponding to approximately a 10-fold difference in the dissociation constant (*K*_d_). Additionally, polar interactions involving the terminal carboxylic acid group were observed more frequently (Figure S7).

A more negative docking score (kcal/mol) indicates stronger predicted binding affinity, while a more negative e-model score (kcal/mol) suggests a more favorable binding pose. Based on these results, compound **7′** demonstrated both lower docking and e-model scores compared to compound **7**, indicating stronger and more stable binding. Notably, the scores for compound **7′** were comparable to those of the positive control, quisqualic acid, suggesting that it may exhibit similarly strong binding affinity to human mGluR1. These findings support the hypothesis that compound **7** is likely hydrolyzed to compound **7′** in biological systems, thereby exerting enhanced activity in its acid form.

### Intracellular Uptake of Compound 7 and Its Conversion to MK-4

Previous studies have revealed several of the mechanisms by which vitamin K compounds exhibits their various bioactivities. For example it has been reported that vitamin K homologues and compounds are converted to MK-4 by UbiA prenyltransferase domain-containing protein 1.^48^ Therefore, we evaluated the cellular uptake of compound **7** and its conversion to MK-4 in MG63 cells, a human osteosarcoma cell line widely used as an in vitro model for studying osteoblast behavior and bone biology. A significant concentration-dependent increase in intracellular concentration was observed for both compound **7** and PK (phylloquinone [vitamin K_1_]; positive control; Figure 8A). With respect to conversion to MK-4, treatment with PKH_2_ (2′,3′-dihydrophylloquinone), which is not a substrate that can be converted to MK-4, showed no conversion to MK-4 (Figure 8B).^49^ However, significant increases in MK-4 concentration were observed in cells treated with 10 μM PK or compound **7**. Furthermore, treatment with 5 μM compound **7** also resulted in a significant increase in MK-4 concentration, suggesting that compound **7** is more easily converted to MK-4 compared with PK (Figure 8B). Consistent with a previous report, about 10% of each vitamin K compound was found to be converted to MK-4 relative to the intracellular concentration.^25^ Thus, although compound **7** was partially converted to MK-4, the majority was absorbed as **7**.

**Figure 8:**
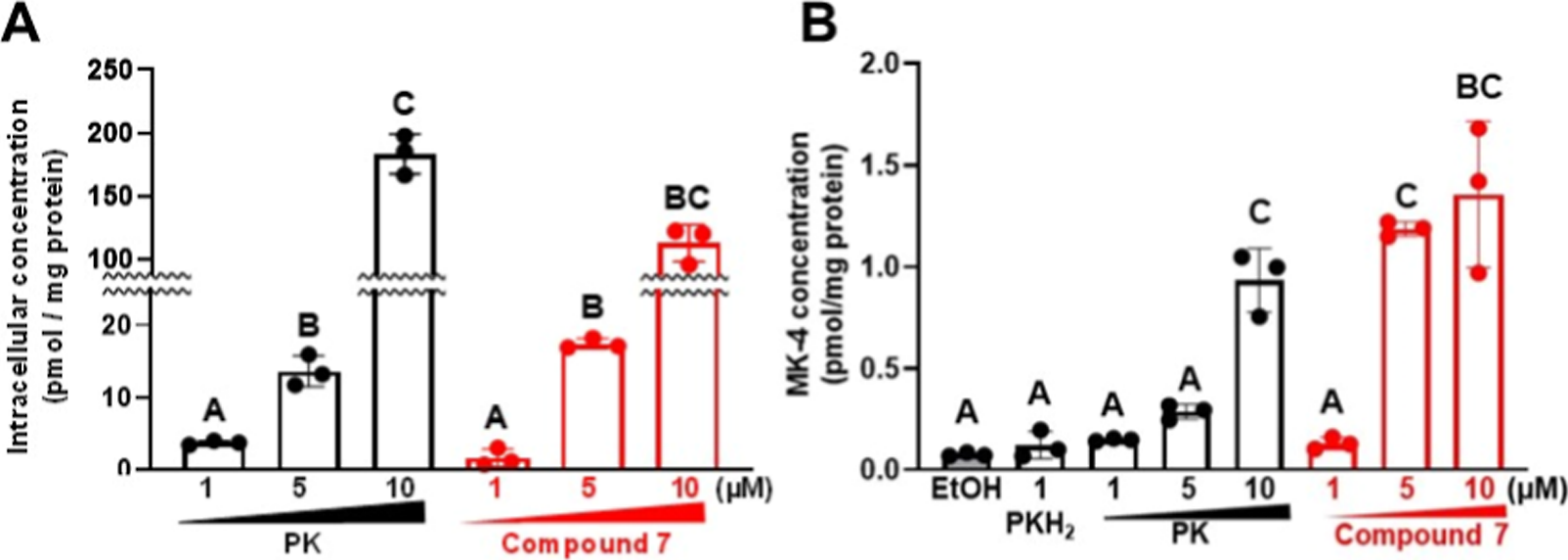
Uptake of compound **7** into MG63 cells and conversion to MK-4. (A) Concentration-dependent uptake of phylloquinone (PK) and compound **7** by MG63 cells. (B) Concentration-dependent conversion of 2′,3′-dihydrophylloquinone (PKH_2_), PK, and compound **7** to MK-4 in MG63 cells. Values not sharing a common letter in each group were significantly different (*n* = 3); *p* < 0.05 (Tukey–Kramer HSD test).

### Pharmacokinetics Analysis of Compound **7** in Mice

Finally, we investigated the in vivo stability of compound **7** by conducting a pharmacokinetics study in C57BL6J mice. Compound **7** was administered orally, and the changes in intracellular uptake and conversion to MK-4 in plasma, liver, and cerebrum were monitored over time. In order for a compound to induce neuronal differentiation, it is necessary for it to penetrate the blood–brain barrier. Compounds with a topological polar surface area greater than 140 Å^2^ are generally considered to have low membrane permeability;^50,51^ however, compound **7** has a topological polar surface area of only 60.44 Å^2^. In addition to its low topological polar surface area, compound **7** was also expected to have excellent blood–brain barrier permeability because of its high fat solubility because of its high fat solubility parameter value (*c* Log *P*, 7.05).

In the plasma, concentrations of compound **7** peaked at 4 h after administration (*C*_max_, 1.72 pmol/mL) and compound **7** was still detected at 12 h after administration (Figure 9A). The MK-4 concentration in plasma increased immediately after administration of compound **7**, compared to the vehicle group, but had returned to baseline at 24 h after administration (Figure 9B). The MK-4 concentration in plasma increased immediately after administration compared to the vehicle group, but had returned to baseline at 24 h after administration. In the liver, concentrations of compound **7** peaked at 4 h after administration (*C*_max_, 34.3 pmol/g) as well as plasma and compound **7** was barely detectable after 12 h (Figure 9C). Surprisingly, MK-4 concentrations in the liver showed exactly the opposite trend as in plasma, with MK-4 concentrations falling immediately after compound **7** administration and not recovering until 24 h later (Figure 9D). In the cerebrum, unlike in the plasma and liver, the concentration of compound **7** peaked at 6 h after administration (*C*_max_, 8.17 pmol/g) and remained significantly higher than in the vehicle group until 24 h (Figure 9E). MK-4 concentrations in the cerebrum increased over time starting immediately after administration of compound **7**, and the maximum cerebral MK-4 concentration was reached at 24 h (Figure 9F).

**Figure 9:**
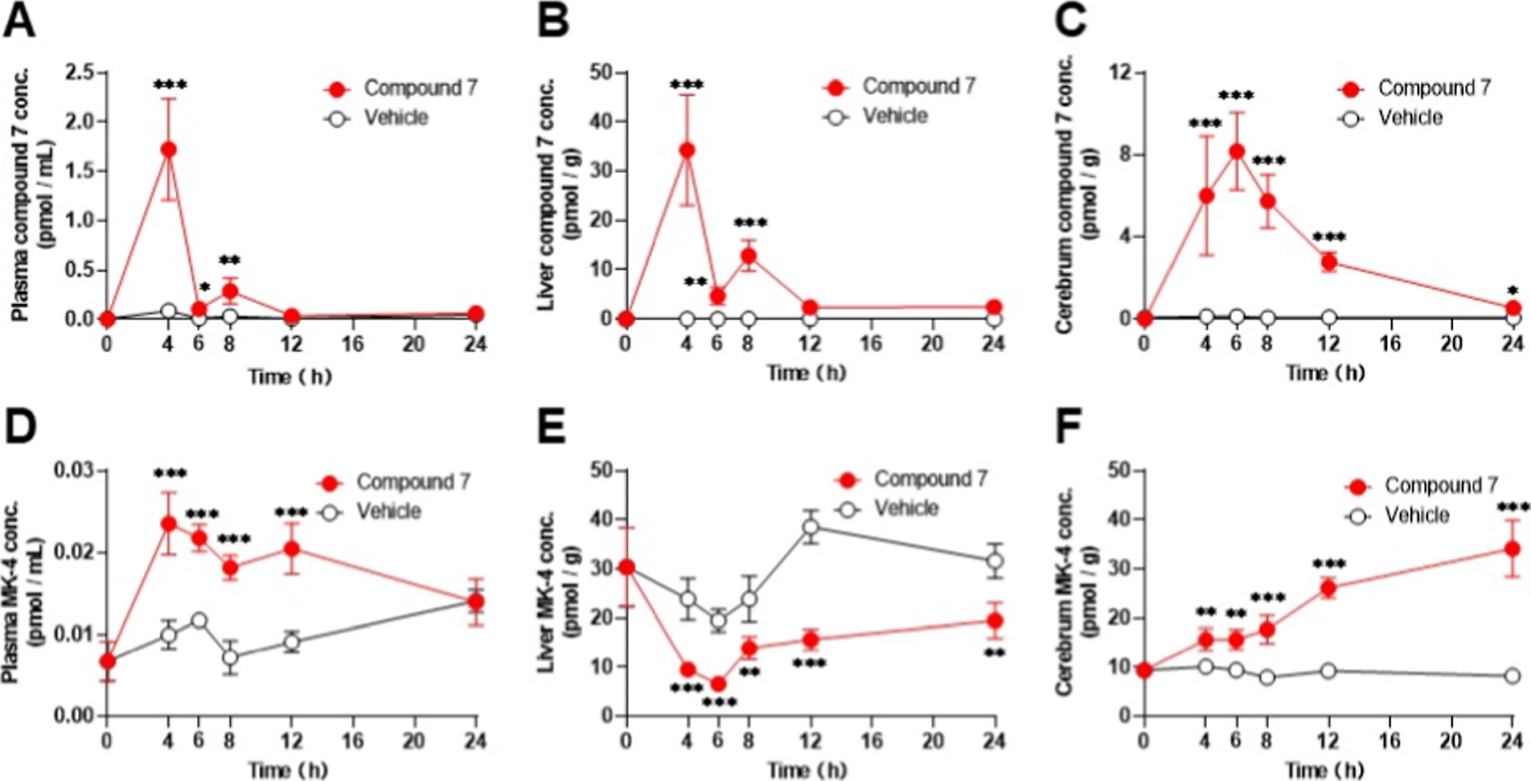
Pharmacokinetics of compound **7** and MK-4 conversion in mice. (A) Time course of compound **7** concentration in plasma. (B) Time course of compound **7** concentration in liver. (C) Time course of compound **7** concentration in cerebrum. (D) Time course of MK-4 concentration in plasma. (E) Time course of MK-4 concentration in liver. (F) Time course of MK-4 concentration in cerebrum. Significant difference from vehicle group at the same time point; *p < 0.05, **p < 0.01, ***p < 0.001 (Student’s *t*-test).

It has been reported that the concentration of deuterium-labeled phylloquinone (PK-*d*_7_) in the liver of mice reaches its peak approximately 3 h after oral administration of 10 μM PK-*d*_7_.^52^ However, the maximum concentration of compound **7** in the liver was only 34.3 pmol/g, indicating that there was very low localization of compound **7** in the liver. Also, the concentration of PK-*d*_7_ in the cerebrum of mice orally administered 10 μM PK-*d*_7_ peaks at approximately 4.5 pmol/g,^52^ whereas the highest concentration of compound **7** in the cerebrum was 8.17 pmol/g, suggesting that compound **7** has a higher cerebral vascular permeability than PK and easily localizes in the brain.

Comparing the concentrations of compound **7** and MK-4 in the cerebrum, the concentration of MK-4 at 24 h was more than twice the maximum concentration of compound **7**. We expect that, similar to PK, compound **7** is partially absorbed by lymphatic vessels and migrates directly to the brain in the early stage up to 6 h after administration, similar to PK. However, it is expected that similarly to PK, compound 7 is cleaved to MD in the small intestine and circulates in the blood, and at 24 h, compound **7** and MD are converted to MK-4 by UBIAD1 in the brain, resulting in increased MK-4 concentration in the brain.^52^ These results indicate that compound **7** enters the brain earlier than MK-4 and is converted to MK-4 by 24 h after administration. The role of vitamin K in the brain is not entirely clear, but vitamin K may inhibit ferroptosis and repair damaged neurons via Gas6.^9^

Since the mechanism of induction of neural stem cell differentiation into neurons was partially elucidated from this study, compound **7** and novel vitamin K analogues are expected to be applicable to the treatment of Alzheimer’s disease and other brain degenerative diseases in the future.

## Conclusions

In this study, we synthesized novel vitamin K derivatives **3**–**9** including known **1** and **2**, which have introduced carboxylic acid esters or conjugated structures, or both, which are characteristic of the side chain structure of retinoic acid. Those synthesized vitamin K analogues showed potent induction of transcriptional activity via SXR and RAR. These compounds also exhibited strong neuronal differentiation-inducing activity, with compounds **7** and **8** showing the greatest activity. The mechanism behind neuronal differentiation induction seems to involve mGluR1 activation, subsequently leading to histone acetylation and transcription factor regulation. Compound **7**, being the most active of the synthesized compounds, showed promising binding affinity to mGluR1, which underlies its role in inducing neuronal differentiation. Moreover, compound **7** was effectively converted to MK-4, highlighting its potential therapeutic effects. A pharmacokinetics analysis in mice revealed the ability of compound **7** to penetrate the blood–brain barrier, enter the cerebrum, and be converted to MK-4, suggesting its potential for development as neuroprotective strategies against neurodegenerative diseases such as Alzheimer’s.

## Experimental Section

^1^H NMR and ^13^C NMR spectra were recorded at 400 and 100 MHz, respectively, using CDCl_3_ as a solvent unless otherwise specified. Chemical shifts are given in parts per million (δ) using tetramethylsilane as an internal standard. High-resolution ESI-MS (ESI-HRMS) was performed with a Micromass Q-TOF mass spectrometer. Column chromatography was carried out with silica gel 60 (70–230 mesh), and preparative thin layer chromatography was run with silica gel 60F_254_. All reagents were purchased from commercial suppliers unless otherwise noted. We confirmed that the purities of the compounds investigated their biological activities were satisfied more than 95%.

### Methyl (2*E*,6*E*)-2,6-Dimethyl-8-(3-methyl-1,4-dioxo-1,4-dihydronaphthalen-2-yl) Octa-2,6-dienoate (**3**)

To a solution of the carboxylic acid **1** (100 mg, 0.296 mmol) in CH_2_Cl_2_, ether solution of CH_2_N_2_ was added until the color of the mixture kept yellow at 0 °C and then stirred at room temperature for 1 h. After the reaction mixture was concentrated in vacuo, the resulting residue was purified by silica gel column chromatography (*n*-hexane: ethyl acetate = 20:1) to obtain compound **3** (94 mg, 266 μmol) in 90% yield as yellow oil. ^1^H NMR (CDCl_3_, 400 MHz): δ 8.08–8.07 (2H, m), 7.70–7.69 (2H, m), 6.69–6.65 (1H, m), 5.07–5.03 (1H, m), 3.65 (3H, s), 3.39 (2H, d, *J* = 6.8 Hz), 2.29–2.24 (2H, m), 2.18 (3H, s), 2.11 (2H, t, *J* = 3.6 Hz), 1.82 (3H, s), 1.78 (3H. s). ^13^C NMR (CDCl_3_, 100 MHz): δ 185.5, 184.6, 168.7, 145.9, 143.6, 141.8, 133.45, 133.42, 132.2, 127.7, 126.4, 126.3, 120.2, 51.8, 38.3, 27.3, 26.1, 16.4, 15.4, 12.8, 12.5. HRMS ([M + Na]^+^) *m*/*z*: calcd for C_22_H_24_O_4_Na, 375.1572; found, 375.1579.

### Methyl (2*E*,6*E*,10*E*)-2,6,10-Trimethyl-12-(3-methyl-1,4-dioxo-1,4-dihydronaphthalen-2-yl) Dodeca-2,6,10-trienoate (**4**)

Similar to the synthesis of **3** from **1**, the crude product **4**, which was obtained from **2** (100 mg, 0.246 mmol) and ether solution of CH_2_N_2_, was purified with silica gel column chromatography (*n*-hexane: ethyl acetate = 20:1), which gave compound **4** (88 mg, 85%) as a yellow oil. ^1^H NMR (CDCl_3_, 400 MHz): δ; 8.08–8.07 (2H, m), 7.73–7.67 (2H, m), 6.71–6.67 (1H, m), 5.10–5.06 (1H, m), 5.04–5.00 (1H, m), 3.72 (3H, s), 3.37 (2H, d, *J* = 6.8 Hz), 2.31–1.97 (8H, m), 2.19 (6H, s), 1.79 (6H, s). ^13^C NMR (CDCl_3_, 100 MHz): δ: 185.5, 184.6, 168.7, 146.2, 143.4, 142.3, 137.2, 133.8, 133.45, 133.39, 132.1, 132.0, 126.4, 126.3, 124.5, 119.3, 51.8, 39.6, 38.2, 27.3, 26.5, 26.1, 16.5, 16.1, 12.8, 12.4. HRMS ([M + Na]^+^) *m*/*z*: calcd for C_27_H_32_O_4_Na, 443.2198; found, 443.2193.

### 2-Methyl-3-((2*E*,6*E*,8*E*)-3,7,11-trimethyldodeca-2,6,8,10-tetraen-1-yl) Naphthalene-1,4-dione (**5**)

Compound **10** (115 mg, 0.430 mmol) was dissolved in 5 mL of anhydrous THF and cooled to −78 °C. Then *n*-BuLi (244 μL, 0.391 mmol) was gradually added, and the color of the solution became yellow. After stirring at −78 °C for 1 h, compound **12** (42 mg, 0.130 mmol) in anhydrous THF (2 mL) was added dropwise to the reaction mixture. After stirring at −78 °C for 3 h, the mixture was quenched with water and extracted with ethyl acetate 3 times. The combined organic layer was washed with brine and dried over MgSO_4_ to concentrate the product. The residue was purified by silica gel column chromatography (*n*-hexane: ethyl acetate = 20:1) to obtain compound **5** (15 mg, 40.1 μmol) in 30% yield as yellow oil. ^1^H NMR (CDCl_3_, 400 MHz): δ 8.09–8.05 (2H, m), 7.70–7.68 (2H, m), 6.25 (1H, t, *J* = 13.0 Hz), 6.05 (1H, d, *J* = 14.8 Hz), 5.79 (1H, d, *J* = 10.4 Hz), 5.36 (1H, t, *J* = 7.4 Hz), 5.03 (1H, t, *J* = 6.8 Hz), 3.37 (2H, d, *J* = 6.8 Hz), 2.22–2.18 (2H, m), 2.19 (3H, s), 2.05 (2H, t, *J* = 7.6 Hz), 1.80–1.73 (12H, m). ^13^C NMR (CDCl_3_, 100 MHz): δ 185.5, 184.6, 146.1, 143.5, 137.2, 135.0, 134.4, 133.4, 133.3, 132.2, 131.3, 126.4, 126.3, 125.8, 122.9, 119.6, 39.5, 26.9, 26.2, 26.1, 18.5, 16.5, 12.8, 12.5. HRMS ([M + Na]^+^) *m*/*z*: calcd for C_26_H_30_O_2_Na, 397.2143; found, 397.2154.

### 2-Methyl-3-((2*E*,6*E*,10*E*,12*E*)-3,7,11,15-tetramethylhexadeca-2,6,10,12,14-pentaen-1-yl) Naphthalene-1,4-dione (**6**)

Similar to the synthesis of **5** from **10**, the crude product **6**, which was obtained from **11** (239 mg, 0.896 mmol), *n*-BuLi (508 μL, 0.813 mmol) and **12** (106 mg, 0.271 mmol) in anhydrous THF (3 mL) was purified with silica gel column chromatography (*n*-hexane: ethyl acetate = 20:1) to obtain compound **6** (36 mg, 81.3 μmol) in 30% yield as yellow oil. ^1^H NMR (CDCl_3_, 400 MHz): δ 8.09–8.06 (2H, m), 7.70–7.67 (2H, m), 6.29 (1H, t, *J* = 13.0 Hz), 6.10 (1H, d, *J* = 14.8 Hz), 5.85 (1H, d, *J* = 10.8 Hz), 5.37 (1H, t, *J* = 7.4 Hz), 5.08–5.00 (2H, m), 3.37 (2H, d, *J* = 7.2 Hz), 2.19 (3H, s), 2.16–1.99 (8H, m), 1.81–1.78 (9H, m), 1.73 (3H, s), 1.57 (3H, s). ^13^C NMR (CDCl_3_, 100 MHz): δ 185.5, 184.6, 146.2, 143.4, 137.6, 135.2, 134.9, 134.3, 134.1, 133.42, 133.36, 131.8, 126.4, 126.3, 125.8, 124.3, 122.8, 119.2, 39.7, 39.4, 27.1, 26.5, 26.4, 26.2, 26.1, 18.5, 16.5, 16.1, 12.8, 12.5. HRMS ([M + Na]^+^) *m*/*z*: calcd for C_31_H_38_O_2_Na, 465.2764; found, 465.2757.

### Triethyl­(3-methylbut-2-en-1-yl)­phosphonium Bromide (**12**)

1-Bromo-3-methyl-2-butene (**14**) (2.00 g, 13.4 mmol) and triethyl phosphite (1.81 mL, 13.4 mmol) were dissolved in 30 mL of toluene and refluxed at 120 °C for 3 h. Thereafter, the mixture was concentrated in vacuo. Then *n*-hexane (40 mL) was added, and the mixture was refluxed at 80 °C for 1 h. The solution was cooled to room temperature and filtered under reduced pressure. Compound **12** as a white solid was obtained (3.45 g, 12.9 mmol) of in 96% yield as colorless oil. ^1^H NMR (CD_3_OD, 400 MHz): δ 5.18–5.13 (1H, m), 3.14–3.08 (2H, m), 2.31–2.22 (6H, m), 1.83 (3H, s), 1.77 (3H, s), 1.31–1.23 (9H, m). ^13^C NMR (CD_3_OD, 400 MHz): δ 141.5, 108.8, 24.6, 18.3, 17.8, 11.2, 10.7, 4.5, 4.4. HRMS ([M + H]^+^) *m*/*z*: calcd for C_11_H_25_BrP, 267.0857; found, 267.0882.

### Methyl (*E*)-4-(Diethoxyphosphoryl)-2-methylbut-2-enoate (**13**)

Triethyl phosphite (3.49 g, 21.0 mmol) was added to compound **15** (4.00 g, 21.0 mmol), and the mixture was heated under reflux at 120 °C for 5 h. After the reaction, the wall of the flask was washed with CH_2_Cl_2_, and then concentrated using an evaporator and a vacuum pump. After concentration, the residue was purified by silica gel column chromatography (*n*-hexane: ethyl acetate = 1:1) to obtain 3.22 g (14.8 mmol) of compound **13** in a yield of 70% as colorless oil. ^1^H NMR (CDCl_3_, 400 MHz): δ 6.80–6.73 (1H, m), 4.17–4.05 (4H, m), 3.75 (3H, s), 2.76 (2H, dd, *J* = 7.6, 22.0 Hz), 1.89 (3H, s), 1.43–1.24 (6H, m). ^13^C NMR (CDCl_3_, 400 MHz): δ 167.8, 131.5, 130.5, 62.3, 52.0, 26.9, 16.53, 16.51, 12.7. HRMS ([M + H]^+^) *m*/*z*: calcd for C_10_H_20_O_5_P, 251.1048; found, 251.1050.

### Methyl (2*E*,4*E*,6*E*,10*E*)-2,6,10-Trimethyl-12-(3-methyl-1,4-dioxo-1,4-dihydronaphthalen-2-yl) Dodeca-2,4,6,10-tetraenoate (**7**)

Compound **10** (512 mg, 2.05 mmol) dissolved in 4 mL of THF (dehydrated) was cooled to 0 °C under Ar atmosphere. Then *n*-BuLi (1.14 mL, 1.86 mmol) was gradually added, and the mixture was stirred at 0 °C for 30 min. After the color of the solution turned yellow, compound **13** (200 mg, 0.620 mmol) in 2 mL of THF was added dropwise to the solution and stirred at room temperature for 4 h. The mixture was quenched with sat. NH_4_Cl aq. and extracted with ethyl acetate 3 times. The combined organic layer was washed with brine and dried over MgSO_4_ to concentrate the product. After concentration, the residue was purified by silica gel column chromatography (*n*-hexane/ethyl acetate = 15:1) to obtain compound **7** (106 mg, 0.253 mmol) in 40% yield as yellow oil. ^1^H NMR (CDCl_3_, 400 MHz): δ 8.10–8.05 (2H, m), 7.70–7.69 (2H, m), 7.17 (1H, d, *J* = 10.0 Hz), 6.45 (1H, d, *J* = 15.2 Hz), 6.34–6.27 (1H, m), 5.58 (1H, t, *J* = 7.2 Hz), 5.04 (1H, t, *J* = 7.2 Hz), 3.76 (3H, s), 3.37 (2H, d, *J* = 6.8 Hz), 2.19 (3H, s), 2.08–1.95 (4H, m), 1.95 (3H, s), 1.81 (3H, s), 1.77 (3H, s). ^13^C NMR (CDCl_3_, 100 MHz): δ 185.5, 184.6, 169.1, 146.0, 144.5, 139.3, 137.5, 136.9, 136.8, 133.5, 133.4, 132.2, 126.4, 125.4, 121.5, 119.9, 51.8, 39.1, 27.2, 26.1, 16.5, 12.8, 12.4. HRMS ([M + Na]^+^) *m*/*z*: calcd for C_27_H_30_O_4_Na, 441.2036; found, 441.2028.

### Methyl (2*E*,4*E*,6*E*,10*E*,14*E*)-2,6,10,14-Tetramethyl-16-(3-methyl-1,4-dioxo-1,4-dihydronaphthalen-2-yl) Hexadeca-2,4,6,10,14-pentaenoate (**8**)

Similar to the synthesis of **7** from **10**, the crude product **8**, which was obtained from **11** (173 mg, 0.691 mmol) in dehydrated THF (4 mL), *n*-BuLi (390 μL, 0.640 mmol), and **13** in dehydrated THF (1 mL) was purified by silica gel column chromatography (*n*-hexane/ethyl acetate = 15:1) to obtain compound **8** (46 mg, 94.7 μmol) in 37% yield as yellow oil. ^1^H NMR (CDCl_3_, 400 MHz): δ 8.10–8.05 (2H, m), 7.71–7.67 (2H, m), 7.24 (1H, d, *J* = 12.0 Hz), 6.51 (1H, d, *J* = 15.2 Hz), 6.38–6.31 (1H, m), 5.60 (1H, t, *J* = 7.4 Hz), 5.08–5.00 (2H, m), 3.75 (3H, s), 3.37 (2H, d, *J* = 6.8 Hz), 2.22–2.17 (2H, m), 2.19 (3H, s), 2.13–2.07 (2H, m), 2.01–1.95 (7H, m), 1.80 (3H, s), 1.77 (3H, s), 1.57 (3H, s). ^13^C NMR (CDCl_3_, 100 MHz): δ 185.5, 184.6, 169.2, 146.2, 144.8, 143.4, 139.4, 137.5, 137.4, 134.5, 134.1, 133.7, 133.43, 133.38, 132.2, 126.4, 126.3, 125.3, 124.6, 121.3, 119.3, 51.8, 39.7, 39.0, 27.3, 26.5, 26.1, 16.5, 16.1, 12.82, 12.77, 12.4. HRMS ([M + Na]^+^) *m*/*z*: calcd for C_32_H_38_O_4_Na, 509.2662; found, 509.2653.

### 2-Methyl-3-(3-oxobutyl) Naphthalene-1,4-dione (**17**)

Menadione (3.00 g, 17.4 mmol), silver nitrate (4.44 g, 26.1 mmol) and levulinic acid (**16**) (3.03 g, 26.1 mmol) were dissolved in 30 mL of H_2_O and 60 mL of CH_3_CN. To the stirred solution, a solution of ammonium peroxodisulfate (15.9 g, 69.7 mmol) in 30 mL of H_2_O was added dropwise over 2 h at 70 °C. The mixture was further stirred for 30 min at same temperature. After cooling to room temperature, the mixture was diluted with diethyl ether and washed with NaHCO_3_ aq., water, and brine. The organic layer was dried over MgSO_4_, filtered, and concentrated in vacuo. The residue was purified by silica gel column chromatography (*n*-hexane/ethyl acetate = 8:1) to obtain compound **17** (2.15 g, 8.87 mmol) in 51% yield as yellow powder. ^1^H NMR (CDCl_3_, 400 MHz): δ 8.08–8.05 (2H, m), 7.71–7.69 (2H, m), 2.89 (2H, t, *J* = 7.0 Hz), 2.67 (2H, t, *J* = 8.4 Hz), 2.21 (3H, s), 2.18 (3H, s). ^13^C NMR (CDCl_3_, 100 MHz): δ 207.1, 185.1, 184.7, 145.9, 144.2, 133.6, 133.5, 132.2, 126.4, 126.3, 42.0, 30.0, 21.7, 12.7. HRMS ([M + Na]^+^) *m*/*z*: calcd for C_15_H_14_O_3_Na, 265.0835; found, 265.0831.

### 2-Methyl-3-(2-(2-methyl-1,3-dioxolan-2-yl)­ethyl)­naphthalene-1,4-dione (**18**)

To a solution of compound **17** (500 mg, 2.06 mmol) dissolved in benzene (25 mL), ethylene glycol (1.13 mL, 20.6 mmol) and H_2_SO_4_ were added in catalytic amounts, and the mixture was refluxed at 95 °C for 2 h under an Ar atmosphere. The reaction mixture was diluted with ethyl acetate and washed with NaHCO_3_ aq., water, and brine. The organic layer was dried over MgSO_4_, filtered, and concentrated in vacuo. The residue was purified by silica gel column chromatography (*n*-hexane/ethyl acetate = 5:1) to obtain compound **18** (569 mg, 1.99 mmol) in 97% yield as yellow powder. ^1^H NMR (CDCl_3_, 400 MHz): δ 8.08–8.06 (2H, m), 7.71–7.67 (2H, m), 4.02–3.95 (4H, m), 2.75 (2H, t, *J* = 8.2 Hz), 2.21 (3H, s), 1.81 (2H, t, *J* = 8.2 Hz), 1.41 (3H, s). ^13^C NMR (CDCl_3_, 100 MHz): δ 185.4, 184.6, 147.2, 143.3, 133.44, 133.39, 132.28, 132.21, 126.32, 126.29, 109.6, 64.8, 37.3, 23.7, 21.9, 12.5. HRMS ([M + Na]^+^) *m*/*z*: calcd for C_17_H_18_O_4_Na, 309.1097; found, 309.1091.

### 2-(2-(1,4-Dimethoxy-3-methylnaphthalen-2-yl) ethyl)-2-methyl-1,3-dioxolane (**19**)

To a solution of compound **18** (100 mg, 0.349 mmol) dissolved in 20 mL of THF, tetrabutylammonium bromide (23 mg, 69.8 μmol) and 10 mL of sat. Na_2_S_2_O_4_ aq. were added and the mixture was stirred at room temperature for 20 min under an Ar atmosphere. Then 10 mL of 2 M KOH aq. and iodomethane (109 μL, 1.75 mmol) were added to the mixture and stirred at room temperature for 15 h. The resulting mixture was diluted with ethyl acetate and washed with NaHCO_3_ aq., water, and brine. The organic layer was dried over MgSO_4_, filtered, and concentrated in vacuo. The residue was purified by silica gel column chromatography (*n*-hexane/ethyl acetate = 8:1) to obtain compound **19** (74 mg, 0.234 mmol) in 67% yield as colorless oil. ^1^H NMR (CDCl_3_, 400 MHz): δ 8.06–8.00 (2H, m), 7.46–7.39 (2H, m), 4.04–4.01 (4H, m), 3.92 (3H, s), 3.86 (3H, s), 2.94–2.90 (2H, m), 2.42 (3H, s), 1.91–1.87 (2H, m), 1.45 (3H, s). ^13^C NMR (CDCl_3_, 100 MHz): δ 150.3, 150.0, 131.2, 127.5, 127.3, 126.5, 125.5, 125.4, 122.3, 122.2, 109.9, 64.9, 62.3, 61.3, 39.2, 23.8, 22.0, 12.3. HRMS ([M + Na]^+^) *m*/*z*: calcd for C_19_H_24_O_4_Na, 339.1567; found, 339.1561.

### 4-(1,4-Dimethoxy-3-methylnaphthalen-2-yl) Butan-2-one (**20**)

Compound **19** (449 mg, 1.42 mmol) was dissolved in a mixture of THF (3 mL) and 1 M HCl aq. (6 mL) and stirred at room temperature for 19 h. After adding water, the mixture was extracted with ethyl acetate, and washed with brine. The organic layer was dried over MgSO_4_, filtered, and concentrated in vacuo. The residue was purified by silica gel column chromatography (*n*-hexane: ethyl acetate = 8:1) to obtain compound **20** (379 mg, 1.39 mmol) in 98% yield as white powder. ^1^H NMR (CDCl_3_, 400 MHz): δ 8.06–7.98 (2H, m), 7.48–7.43 (2H, m), 3.89 (3H, s), 3.86 (3H, s), 3.06 (2H, t, *J* = 8.0 Hz), 2.70 (2H, t, *J* = 8.0 Hz), 2.39 (3H, s), 2.18 (3H, s). ^13^C NMR (CDCl_3_, 100 MHz): δ 150.3, 150.1, 130.0, 127.7, 127.2, 126.2, 125.7, 125.5, 122.3, 122.2, 62.2, 61.4, 43.9, 29.9, 21.6, 12.4. HRMS ([M + Na]^+^) *m*/*z*: calcd for C_17_H_20_O_3_Na, 295.1305; found, 295.1298.

### Methyl (*E*)-5-(1,4-Dimethoxy-3-methylnaphthalen-2-yl)-3-methylpent-2-enoate (**21**)

To a solution of trimethylphosphonoacetate (605 mg, 3.32 mmol) in 10 mL of dehydrated THF, NaH (113 mg, 2.82 mmol) was gradually added at 0 °C under Ar atmosphere. After the mixture was stirred at 0 °C for 1 h, compound **20** (452 mg, 1.66 mmol) dissolved in 5 mL of dehydrated THF was added dropwise to the mixture. Then the reaction mixture was stirred at 65 °C for 24 h. After cooling to room temperature, a small amount of methanol was added and quenched with sat. NH_4_Cl aq. The mixture was diluted with ethyl acetate, washed with brine. The organic layer was dried over MgSO_4_, filtered, and concentrated in vacuo. The residue was purified by silica gel column chromatography (*n*-hexane/ethyl acetate = 8:1) to obtain compound **21** (371 mg, 1.13 mmol) in 68% yield as colorless oil. ^1^H NMR (CDCl_3_,400 MHz): δ 8.06–7.99 (2H, m), 7.47–7.44 (2H, m), 5.79 (1H, s), 3.91 (3H, s), 3.86 (3H, s), 3.71 (3H, s), 2.95 (2H, t, *J* = 8.4 Hz), 2.41 (3H, s), 2.38–2.29 (2H, m), 2.29 (3H, s). ^13^C NMR (CDCl_3_, 100 MHz): δ 171.1, 146.7, 144.3, 125.8, 125.5, 124.6, 121.8, 121.5, 114.7, 74.0, 51.7, 43.7, 31.1, 24.8, 31.1, 24.8, 20.7, 12.1. HRMS ([M + Na]^+^) *m*/*z*: calcd for C_20_H_24_O_4_Na, 351.1567; found, 351.1558.

### (*E*)-5-(1,4-Dimethoxy-3-methylnaphthalen-2-yl)-3-methylpent-2-en-1-ol (**22**)

To a solution of compound **21** (294 mg, 0.895 mmol) in 4 mL of dehydrated THF, lithium aluminum hydride (136 mg, 3.58 mmol) was slowly added to this solution at 0 °C under an Ar atmosphere. After stirring for 15 min, the mixture was warmed to room temperature and stirred for 24 h. After cooling again to 0 °C, 1 mL of water and then 1 mL of 15% NaOH aq. were added to the reaction mixture. The mixture was filtered through Celite, and the filtrate was extracted with ethyl acetate 3 times. The combined organic layer was washed with brine, dried over MgSO_4_, filtered, and concentrated in vacuo. The residue was purified by silica gel column chromatography (*n*-hexane/ethyl acetate = 4:1) to obtain compound **22** (252 mg, 0.839 mmol) in 94% yield as colorless oil. ^1^H NMR (CDCl_3_, 400 MHz): δ 8.06–8.00 (2H, m), 7.47–7.45 (2H, m), 5.48 (1H, dd, *J* = 6.4 Hz), 4.18 (2H, d, *J* = 6.8 Hz), 3.91 (3H, s), 3.87 (3H, s), 2.93 (2H, t, *J* = 8.2 Hz), 2.42 (3H, s), 2.27–2.25 (2H, m), 1.82 (3H, s). ^13^C NMR (CDCl_3_, 100 MHz): δ 150.3, 150.0, 139.9, 131.0, 127.6, 127.2, 126.3, 125.54, 125.47, 123.6, 122.3, 62.3, 61.4, 59.4, 39.9, 26.3, 16.5, 12.4. HRMS ([M + Na]^+^) *m*/*z*: calcd for C_19_H_24_O_3_Na, 323.1618; found, 323.1613.

### (*E*)-2-(5-Hydroxy-3-methylpent-3-en-1-yl)-3-methylnaphthalene-1,4-dione (**23**)

To a solution of compound **22** (158 mg, 0.526 mmol) in 4 mL of CH_3_CN, ceric ammonium nitrate (577 mg, 1.052 mmol) dissolved in 2 mL of H_2_O was added at 0 °C under an Ar atmosphere. The mixture was warmed to room temperature and stirred for 40 min. The mixture was diluted with water and extracted with ethyl acetate 3 times. The combined organic layer was washed with brine, dried over MgSO_4_, filtered, and concentrated in vacuo. The residue was purified by silica gel column chromatography (*n*-hexane/ethyl acetate = 2:1) to obtain compound **23** (72 mg, 0.268 mmol) in 82% yield as yellow oil. ^1^H NMR (CDCl_3_, 400 MHz): δ 8.10–8.06 (2H, m), 7.71–7.69 (2H, m), 5.46 (1H, t, *J* = 6.8 Hz), 4.15 (2H, d, *J* = 6.8 Hz), 2.77 (2H, t, *J* = 8.0 Hz), 2.20 (3H, s), 2.19 (2H, t, *J* = 13.2 Hz), 1.79 (3H, s). ^13^C NMR (CDCl_3_, 100 MHz): δ 185.3, 184.7, 146.8, 143.6, 138.8, 133.5, 132.2, 126.3, 124.4, 59.4, 38.2, 25.9, 16.4, 12.7. HRMS ([M + H]^+^) *m*/*z*: calcd for C_17_H_19_O_3_, 271.1329; found, 271.1324.

### (*E*)-3-Methyl-5-(3-methyl-1,4-dioxo-1,4-dihydronaphthalen-2-yl) Pent-2-enal (**24**)

To a solution of compound **23** (116 mg, 0.429 mmol) in 4 mL of dehydrated CH_2_Cl_2_, MnO_2_ (1.12 g, 12.9 mmol) was added, and the reaction mixture was stirred at room temperature for 8 h. The mixture was filtered through Celite, and the filtrate was concentrated in vacuo. Thereafter, purification was performed by silica gel column chromatography (*n*-hexane: ethyl acetate = 6:1) to obtain compound **24** (90 mg, 0.335 mmol) in 78% yield as yellow powder. ^1^H NMR (CDCl_3_, 400 MHz): δ 10.0 (1H, d, *J* = 8.0 Hz), 8.11–8.05 (2H, m), 7.73–7.69 (2H, m), 5.92 (1H, d, *J* = 8.0 Hz), 2.85 (2H, t, *J* = 8.0 Hz), 2.37 (2H, t, *J* = 8.0 Hz), 2.29 (3H, s), 2.21 (3H, s). ^13^C NMR (CDCl_3_, 100 MHz): δ 191.2, 185.0, 184.5, 162.1, 145.4, 144.0, 133.7, 132.0, 125.5, 126.4, 40.0, 25.2, 17.7, 12.8. HRMS ([M + Na]^+^) *m*/*z*: calcd for C_17_H_16_O_3_Na, 291.0992; found, 291.0985.

### Methyl (*E*)-4-(Diethoxyphosphoryl)-3-methylbut-2-enoate (**25**)

To a solution of triethyl phosphite (2.01 g, 12.1 mmol) in toluene, compound **27** (2.34 g, 12.1 mmol) was added and the mixture was refluxed at 120 °C for 5 h. After cooling to room temperature, the mixture was concentrated in vacuo. The residue was purified by silica gel column chromatography (*n*-hexane: ethyl acetate = 1:1) to give compound **25** (1.43 g, 5.64 mmol) in 47% yield as colorless oil. ^1^H NMR (CDCl_3_, 400 MHz): δ 5.80 (1H, s), 4.18–4.12 (4H, m), 3.70 (3H, s), 2.68 (2H, d, *J* = 16.0 Hz), 2.31 (3H, s), 1.30 (6H, m). ^13^C NMR (CDCl_3_, 100 MHz): δ 166.5, 150.1, 119.5, 62.4, 51.1, 39.3, 37.9, 20.1, 16.4. HRMS ([M + Na]^+^) *m*/*z*: calcd for C_10_H_19_O_5_NaP, 273.0862; found, 273.0854.

### Methyl (2*E*,4*E*,6*E*)-3,7-Dimethyl-9-(3-methyl-1,4-dioxo-1,4-dihydronaphthalen-2-yl)­nona-2,4,6-trienoate (**9**)

To a solution of compound **25** (319 mg, 1.28 mmol) in 2 mL of dehydrated THF, *n*-BuLi (648 μL, 1.06 mmol) was slowly added at −78 °C under an Ar atmosphere. After the mixture was stirred at – 78 °C for 1 h, compound **24** in 3 mL of dehydrated THF was added to the reaction solution and stirred at 0 °C for 3 h. The mixture was quenched with sat. NH_4_Cl aq. and extracted with ethyl acetate 3 times. The combined organic layer was washed with brine, dried over MgSO_4_, filtered, and concentrated in vacuo. The residue was purified by silica gel column chromatography (*n*-hexane: ethyl acetate = 8:1) to obtain compound **9** (97 mg, 0.266 mmol) in 63% yield as yellow powder. ^1^H NMR (CDCl_3_, 400 MHz): δ 8.10–8.05 (2H, m), 7.71–7.69 (2H, m), 6.84–6.78 (1H, m), 6.17 (1H, d, *J* = 7.6 Hz), 6.01 (1H, d, *J* = 5.6 Hz), 5.74 (1H, s), 3.71 (3H, s), 2.80 (2H, t, *J* = 8.0 Hz), 2.33 (3H, s), 2.26–2.21 (2H, m), 2.21 (3H, s), 1.96 (3H, s). ^13^C NMR (CDCl_3_, 100 MHz): δ 185.3, 184.6, 167.6, 153.2, 146.5, 143.6, 142.5, 134.3, 133.5, 132.2, 130.8, 126.7, 126.4, 125.7, 118.0, 51.1, 39.0, 26.1, 17.2, 13.9, 12.8. HRMS ([M + Na]^+^) *m*/*z*: calcd for C_23_H_24_O_4_Na, 387.1567; found, 387.1558.

### Methyl (*E*)-4-Bromo-3-methylbut-2-enoate (**27**)

To a solution of methyl-3,3-dimethyl acrylate (**26**) (5.00 g, 43.8 mmol) in 50 mL of dehydrated CH_2_Cl_2_, *N*-bromo succinimide (9.36 g, 52.6 mmol) was added at 0 °C under an Ar atmosphere. Then the mixture was stirred at 40 °C for 18 h. The solution was extracted with ethyl acetate 3 times. The combined organic layer was washed with sat. Na_2_S_2_O_3_aq., water, and brine, dried over MgSO_4_, filtered, and concentrated in vacuo. The residue was purified by silica gel column chromatography (*n*-hexane/ethyl acetate = 20:1) to give compound **27** (3.18 g, 16.4 mmol) in 37% yield as colorless oil. ^1^H NMR (CDCl_3_, 400 MHz): δ 5.97 (1H, s), 3.95 (2H, s), 3.72 (3H, s), 2.28 (3H, s). ^13^C NMR (CDCl_3_, 100 MHz): δ 166.4, 152.8, 119.0, 51.4, 38.3, 17.3. HRMS ([M + H]^+^) *m*/*z* calcd for C_6_H_10_O_2_Br, 192.9859; found, 192.9857.

### Ethics Statement

All animal experiments were performed in accordance with the Shibaura Institute of Technology Animal Experiment Guidelines (#21013) and were approved by the Shibaura Institute of Technology Animal Research and Ethics Committee, Saitama, Japan. All surgeries were performed under sodium pentobarbital anesthesia, and efforts were made to minimize suffering.

### Evaluation of SXR- and RAR-Mediated Transcriptional Activity

Human liver cancer-derived HepG2 cells were grown in Dulbecco’s modified Eagle’s medium supplemented with 10% fetal bovine serum (FBS) and 1% penicillin/streptomycin. The cells were cultured under 5% CO_2_ conditions at 37 °C. Passage was performed every 5 days to maintain the cells. To evaluate transcriptional activity, HepG2 cells were seeded into 96-well plates and incubated for 48 h. To obtain a transfection solution for assessment of SXR-mediated transcriptional activity, Opti-MEM medium was loaded with pcDNA3.1-FLAG-SXR, pGL4.10-CYP3A4 promoter, and pRL-CMV, and was then was mixed with Lipofectamine 2000 and incubated at room temperature for 20 min. For assessment of RAR-mediated transcriptional activity, Opti-MEM were mixed with pGL3-RARE, pRL-CMV, and Lipofectamine 2000, and then incubated at room temperature for 20 min. Except for the medium, the transfection solution was pipetted dropwise over the cells and the plates incubated for a further 3 h. The transfection solutions were then removed and cells were incubated for an additional 21 h at 37 °C. The vitamin K homologues and derivatives were incubated in Dulbecco’s modified Eagle’s medium containing 2.5% FBS and 1% penicillin/streptomycin; vitamin K homologues and derivatives were 1 μM (SXR transcriptional activity) and 10 μM (RAR transcriptional activity). In all cases, the ethanol content in the medium was 0.1%. Except for the medium, medium containing the compound of interest was added to each well and the plates incubated for 48 h. The cells were washed with PBS after removing the medium containing the compound, and the cells were measured for firefly luciferase activity using the Dual-Luciferase Reporter Assay System. A fluorometer was used to measure the luciferase luminescence. Transcriptional activity was calculated by dividing the measured value of firefly luciferase activity by the measured value of murine luciferase activity, corrected for the vector transfer efficiency. The data are reported as fold-induction for transcriptional activity.

### Isolation of Mouse Cerebrum-Derived Neural Stem Cells

Fetuses were harvested from euthanized female C57BL/6J mice at 14.5 days of gestation. Under stereomicroscopic observation, the cerebrum was isolated from the brains of the explanted fetuses and washed 5 times with L-15 medium. The obtained cerebrums were enzymatically dissociated with trypsin solution and DNase I solution at 37 °C. DMEM (low glucose) medium containing 10% FCS was added, the cerebrum was loosened by pipetting, and the cells were dispersed and centrifuged. The cells were suspended in DMEM medium containing 10% FCS and seeded at 3 × 10^6^ cells/well in coated with polyethylenimine supports neural stem cell attachment and growth glass bottom culture dishes. Neural stem cells expressing Hes1 were cultured at 37 °C in the presence of 5% CO_2_.

### Evaluation of Neuronal Differentiation

The obtained neural stem cells were cultured in DMEM medium containing 10% FBS supplemented with 1 μM vitamin K homologue or analogue for 5 days with the medium refreshed every 48 h. Ethanol (final concentration, 0.1%) was used as the control treatment. At the end of the culture period, the cells were fixed with PBS(−) containing 4% paraformaldehyde at room temperature, washed with PBS(−), treated with PBS(−) containing 0.2% Triton X-100, and left at room temperature. The neural stem cells were treated with PBS containing 10% goat serum with anti-Map2 antibody as the primary antibody and allowed to react overnight at 4 °C. The next day, the cells were treated with CF594 dye as a secondary antibody and samples of the cell culture were mounted with DAPI-containing mounting medium for microscopy analysis. Nuclei were stained blue with DAPI (excitation wavelength, 360 nm; fluorescence wavelength, 460 nm) and neurons were stained red with CF594 (excitation wavelength, 593 nm; fluorescence wavelength, 614 nm) and observed under a fluorescence microscope using pseudoconfocal mode.

### Quantification of Genes Related to Neuronal Differentiation by Vitamin K Analogues

Neural stem cells were seeded in 6-well plates at 3 × 10^6^ cells/well and cultured for 24 h at 37 °C. The cells were then treated with 1 μM vitamin K homologue or analogue, or EtOH as the control, every other day for 4 days. The cells were then collected and mRNA was extracted. RT-PCR was used to quantify the mRNA levels of *Map2* and β*-actin*. The results were normalized to the EtOH control. β-actin was used as a housekeeping gene. The ratio of Map2/β-actin mRNA levels was used as an index of the differentiation-inducing activity of the vitamin K homologues and analogues on neurons.

### Transcriptome Analysis of Genes Expressed in the Cerebrum

Neural stem cells isolated from mouse embryos were seeded in 6-well plates and treated with EtOH (negative control, final concentration 0.1%), MK-4 (positive control), MK-3-*m*-methylphenyl (MP3) as a compound that increases neuronal differentiation, and MK-2-*o*,*m*-dimethylphenyl (23P2) as a compound that suppresses neuronal differentiation.^16^ Before use, 1 μM of the compounds were added to L-DMEM medium containing 10% FBS. Cells were cultured for 5 days at 37 °C in the presence of 5% CO_2_ with the medium refreshed every 48 h. After incubation, the cells were collected and RNA was extracted. The extracted RNA was prepared to a RIN value of 7 or higher and a concentration of 100 μg/μL. Transcriptome analysis (RNA-seq) of genes expressed in the cerebrum were performed on the extracted RNA using a Next Generation Sequencer. Gene expressions under treatment with MK-4, MP3, or 23P2 as a ratio to gene expression under treatment with EtOH control were calculated as log values and quantitatively compared. Heat maps were generated using statistical analysis software and color-coded for genes whose expression levels decreased by less than half (blue) and increased by more than 2-fold (red) compared to controls.

### Analysis of Neuronal Differentiation-inducing Activity Using mGluR Inhibitors

The mGluRs are broadly divided into three groups: Group 1 includes mGluR1 and mGluR5; Group 2 includes mGluR2 and mGluR3; and Group 3 includes mGluR4, mGluR6, mGluR7, and mGluR8 (Figure 6B). Inhibitors of the Group 1 mGluRs include PHCCC (*N*-phenyl-7-(hydroxyimino)­cyclopropa­[*b*]­chromen-1a-carboxamide); inhibitors of the Group 2 mGluRs include (*RS*)-APICA ((*RS*)-1-amino-5-phosphonoindan-1-carboxylic acid); and inhibitors of the Group 3 mGluRs include CPPG ((*R,S*)-α-cyclopropyl-4-phosphonophenylglycine] (Figure 6B). mGluR1-specific inhibitors include JNJ16259685 [(3,4-dihydro-2*H*-pyrano­[2,3-*b*]­quinolin-7-yl)-(*cis*-4-methoxycyclohexyl)-methanone], and mGluR5-specific inhibitors include MPEP [2-methyl-6-(phenylethynyl)­pyridine hydrochloride]. For neural stem cells isolated from mouse embryos, 1 μM of MK-4 was added simultaneously with 10 μM of PHCCC, 5 μM of (*RS*)-APICA, or 5 μM of CPPG in L-DMEM medium containing 10% FBS, respectivel.^53,54^ PHCCC was dissolved in DMSO, (*RS*)-APICA and CPPG were dissolved in 0.1 M NaOH, JNJ16259685 and MPEP were dissolved in EtOH, and the dissolution reagents were used as controls (0.1% content in the medium). Cells were cultured for 5 days at 37 °C in the presence of 5% CO_2_ with the medium refreshed every 48 h.

### mGluR1 Docking Simulation Analysis

Human mGluR1 (Uniprot ID: Q13255) and mouse mGluR1 (Uniprot ID: P97772) were retrieved from the AlphaFold Protein Structure Database (https://academic.oup.com/nar/article/50/D1/D439/6430488?login=true). The three-dimensional structures of the chemicals were constructed using Open Babel (https://jcheminf.biomedcentral.com/articles/10.1186/1758-2946-3-33). Subsequent analyzes were conducted with the Schrödinger Small Molecule Discovery suite (Schrödinger, New York, NY, USA). The stereostructures of proteins and ligands were subjected to hydrogenation, structural refinement, and energy minimization using the OPLS4 force field (https://pubs.acs.org/doi/full/10.1021/acs.jctc.1c00302). Grid box was defined by a 30-Å^3^ space centered on the endogenous ligand quisqualic acid, fitted from its Protein Databank structure (PDBID: 6N50 10.1038/s41586-019-0881-4) using AlphaFill (https://www.nature.com/articles/s41592-022-01685-y). Ligand docking was performed via induced-fit docking using Glide XP (https://pubs.acs.org/doi/10.1021/jm051256o). Each ligand–protein complex was subjected to a 50.00 ns molecular dynamics simulation with Desmond software (https://dl.acm.org/doi/proceedings/10.1145/1188455 D.E. Shaw Research, New York, NY, USA) after neutralization with the TIP3P water model and addition of sodium and chloride ions. Sampling comprised 2500 trajectories under conditions of 300 K and atmospheric pressure using the OPLS4 force field.

### Analysis of Cellular Uptake and MK-4 Conversion of Compound **7**

MG63 osteoblast-like cells were seeded in a 6-well plate, and either PKH_2_, which is not a substrate for conversion to MK-4, was added at a final concentration of 1 μM, or PK or compound **7** was added at a final concentration of 1, 5, or 10 μM. Each compound was dissolved in ethanol so that the ethanol content in the medium was 0.1%; after 24 h, the medium was removed and the cells were washed twice with PBS(−), detached with a cell scraper, and collected in brown centrifuge tubes. As previously reported, an internal standard solution of [^18^O]-PK and [^18^O]-MK-4 dissolved at 3.6 ng/mL in ethanol and hexane was added to the brown centrifuge tube and mixed for 5 min^48,52,55^ The solution was then centrifuged at 4 °C, 2500 rpm for 10 min. The supernatant was separated and removed under reduced pressure with an evaporator, and the residue was dissolved in methanol. The resulting sample was examined by ultraperformance liquid chromatography-atmospheric pressure chemical ionization tandem mass spectrometry.

### Pharmacokinetics Analysis of Compound 7 in Mice

Forty-eight 8 week-old male C57BL/6J mice were used for the experiments. Compound **7** was prepared to a concentration of 10 μmol/mL in ethanol solution containing 10% SDS and 0.1% NP-40). Vehicle was prepared as 0.1% SDS, 0.1% NP-40 solution, and 10% ethanol. Mice were fasted for 12 h prior to dosing, and each solution was administered as a single dose. Blood, liver, and cerebrum were harvested from the treated mice at 0, 4, 6, 8, 12, and 24 h after administration (four mice per time point). Blood was mixed well with heparin, centrifuged at 4 °C, 3000 rpm for 10 min, and the supernatant was collected to obtain plasma. A sample of the plasma (100 μL) was then placed in a brown centrifuge tube and diluted with Milli-Q water to a total volume of 1 mL. The liver and cerebrum were wet weighed and then crushed with a bead crusher. Ethanol was then added to the crushed tissue and the mixture was transferred to a brown centrifuge tube. Ethanol solution containing 100 ng/mL of [^18^O]-PK and [^18^O]-MK-4 as internal standards, ethanol, Milli-Q water, and hexane were added and vortexed for 5 min. The solution was centrifuged at 4 °C, 2500 rpm for 10 min, and the supernatant was separated. The solvent was removed under reduced pressure using a rotary evaporator. The resulting residue was dissolved in 100 μL of methanol and subjected to ultraperformance liquid chromatography–atmospheric pressure chemical ionization tandem mass spectrometry.

## Supplementary Material





## Notes

^1^H NMR and ^13^C NMR spectra of **5**–**9**, **10**–**13**, **17**–**25**, and **27**; HPLC data of **3–9** used in biological assays; Biological assay data; Molecular docking analysis (PDF)
A binding frequency analysis of MK-4, compound **7**, MP3, and 23P2 (MP4)

## Abbreviations

MK-*n*: menaquinone-*n*
ATRA: all-trans retinoic acid
SXR: steroid and xenobiotic receptor
RAR: retinoic acid receptor
Map2: microtubule-associated protein 2
