# Supplementary material for: A New Class of Vitamin K Analogues Containing the Side Chain of Retinoic Acid Have Enhanced Activity for Inducing Neuronal Differentiation
Source: ACS Chem Neurosci. 2025 Jul 3;16(15):2812–28. doi: 10.1021/acschemneuro.5c00111 (PMC12333027; doi:10.1021/acschemneuro.5c00111)
Supplement: Supplementary file 1 [file cn5c00111_si_001.pdf]

Supporting Information

A New Class of Vitamin K Analogues Containing the Side Chain of Retinoic Acid Have Enhanced Activity for Inducing Neuronal Differentiation

*Yoshihisa Hirota†, Taiki Sato‡, Rina Watanabe†, Kazuki Takeda§, ||, Sho Sano†, Satoshi Asano†, Yuki Shibahashi†, Yumi Yasuda‡, Yuta Takagi†, Yutaro Yamashita†, Wu YuXin†, Mikino Arakawa†, Yuri Maitani†, Vannessa Lawai¶, Kurumi Nakagawa†, Natsuko Furukawa‡, Atsuko Takeuchi**, Chisato Tode**, Maya Kamao††, Akimori Wada‡‡, Zainab Ngaini¶, and Yoshitomo Suhara‡, **

†Laboratory of Biochemistry, Department of Bioscience and Engineering, College of Systems Engineering and Science, Shibaura Institute of Technology, 307 Fukasaku, Minuma-ku, Saitama 337-8570, Japan

‡Laboratory of Organic Synthesis and Medicinal Chemistry, Department of Bioscience and Engineering, College of Systems Engineering and Science, Shibaura Institute of Technology, 307 Fukasaku, Minuma-ku, Saitama 337-8570, Japan

§Laboratory of Toxicology, School of Veterinary Medicine, Kitasato University, E23-35-1, Towada, Aomori 034-0021, Japan

|| Department of Computer Science, Tokyo Institute of Technology, 4259-J3-1818, Nagatsuta-cho, Midori-ku, Yokohama-shi, Kanagawa 226-0026, Japan

¶ Faculty of Resource Science and Technology, Universiti Malaysia Sarawak, 94300 Kota Samarahan, Sarawak, Malaysia

** Instrumental Analysis Center, Kobe Pharmaceutical University, 4-19-1 Motoyamakita-machi, Higashinada-ku, Kobe 658-8558, Japan

†† Extension Center, Kobe Pharmaceutical University, 4-19-1 Motoyamakita-machi, Higashinada-ku, Kobe 658-8558, Japan

‡‡ Department of Life Science for Organic Chemistry, Kobe Pharmaceutical University, 4-19-1 Motoyamakita-machi, Higashinada-ku, Kobe 658-8558, Japan

* *Correspondence:suhara@sic.shibaura-it.ac.jp*

Contents:

The Supporting Information is available free of charge on the ACS Publications website.
^{1}H NMR and ^{13}C NMR spectra of **5** – **9**, **10** – **13**, **17** – **25**, and **27**; HPLC data of **3–9** used in biological assays; Biological assay data; Molecular docking analysis (file type, PDF)

I. ^{1}H and ^{13}C NMR chart of compounds 5 – 9, 10 – 13, 17 – 25, and 27 ……………. S3 – S21

II. HPLC data of compounds 3 – 9 used in biological assays…………………………S22 –S26

III. Supplementary Fig.1 – Fig.6 ………………………………………………………. S27-S32

I. ^{1}H and ^{13}C NMR chart of compounds 5 – 9, 10 – 13, 17 – 25, and 27

^{1}H NMR chart of compound **5**

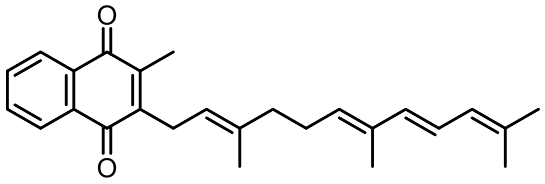

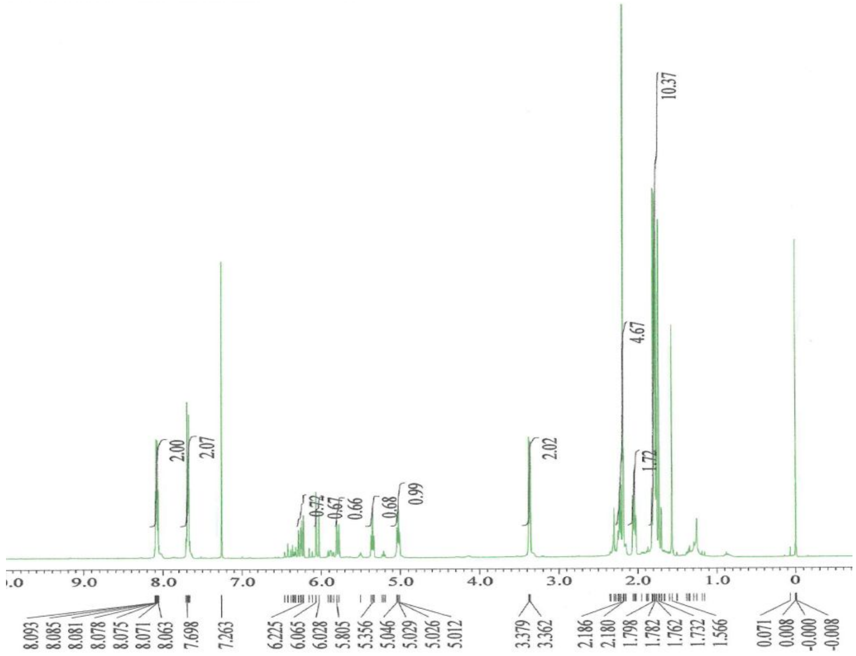

^{13}CNMR chart of compound **5**

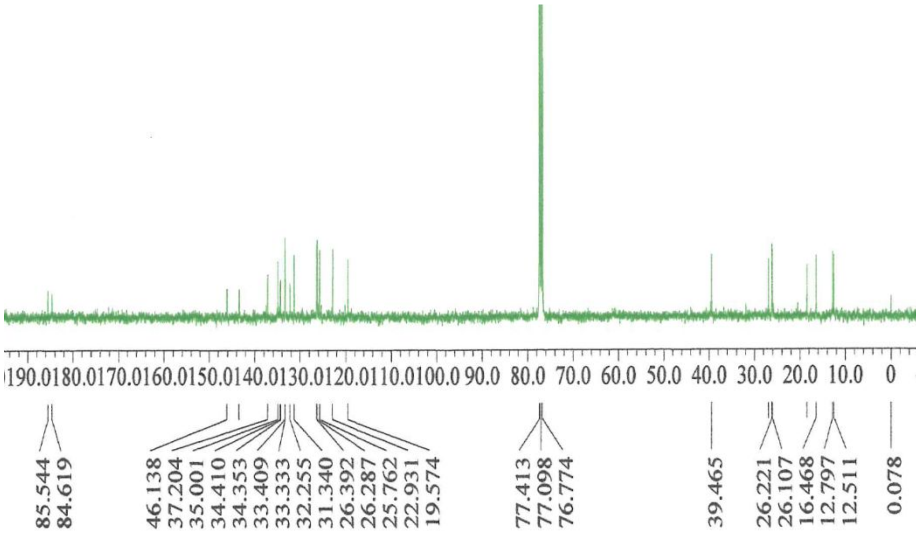

^{1}H NMR chart of compound **6**

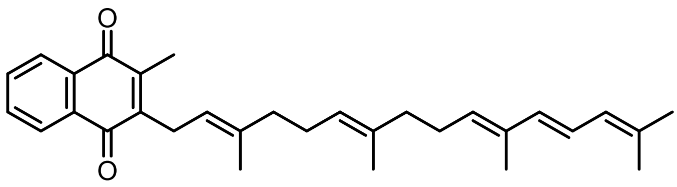

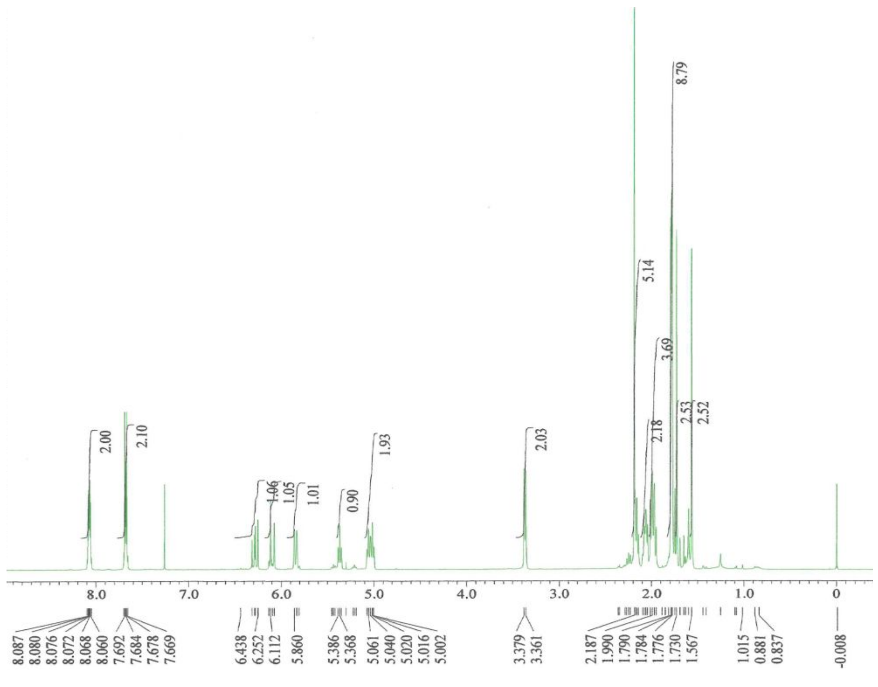

^{13}C NMR chart of compound **6**

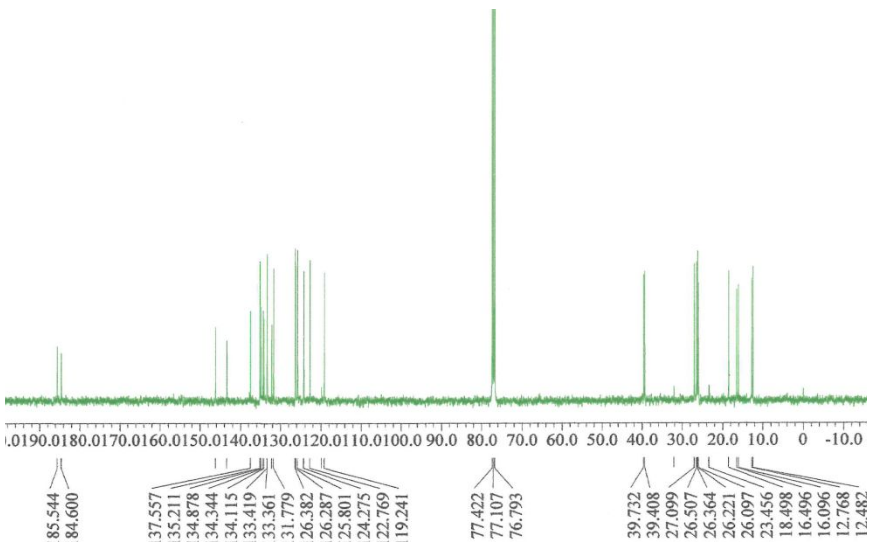

^{1}H NMR chart of compound **10**

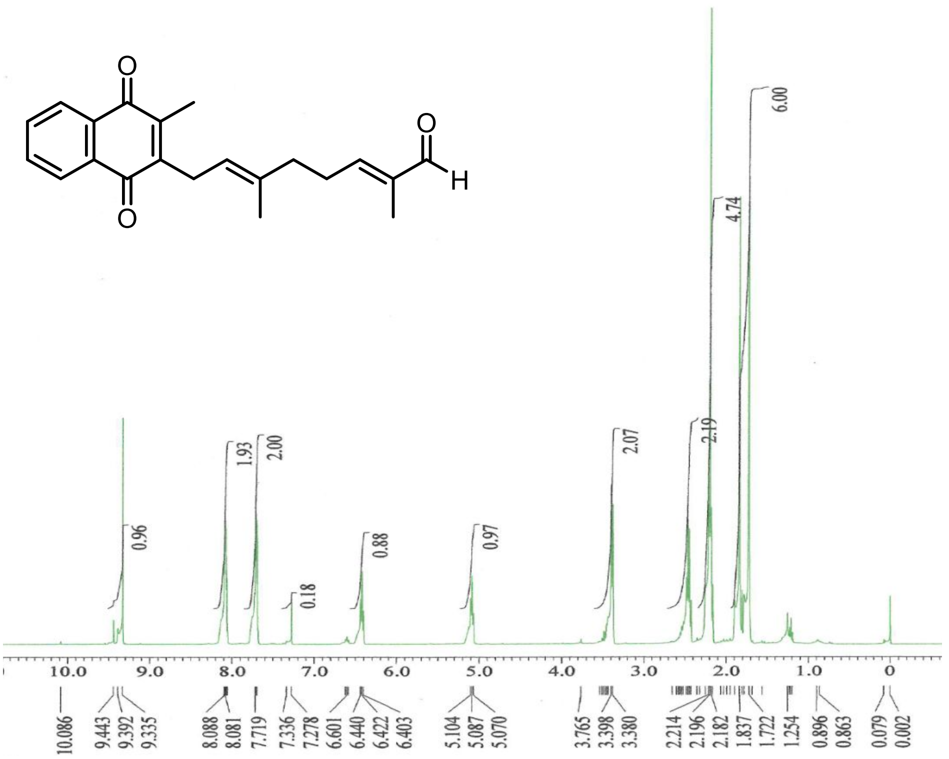

^{13}C NMR chart of compound **10**

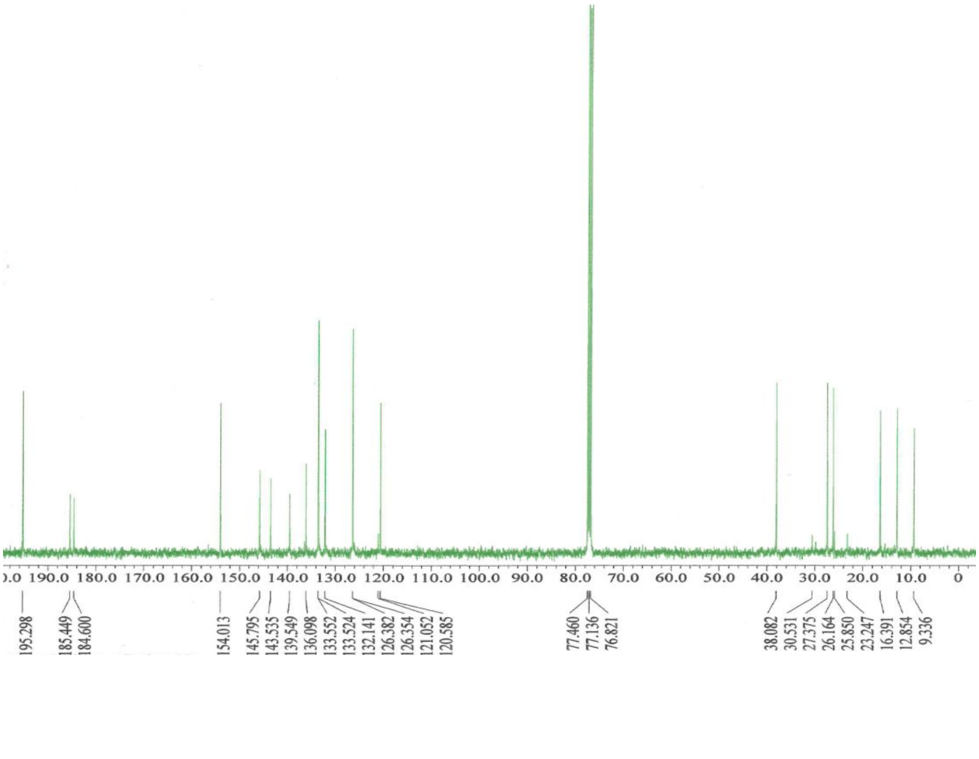

^{1}H NMR chart of compound **11**

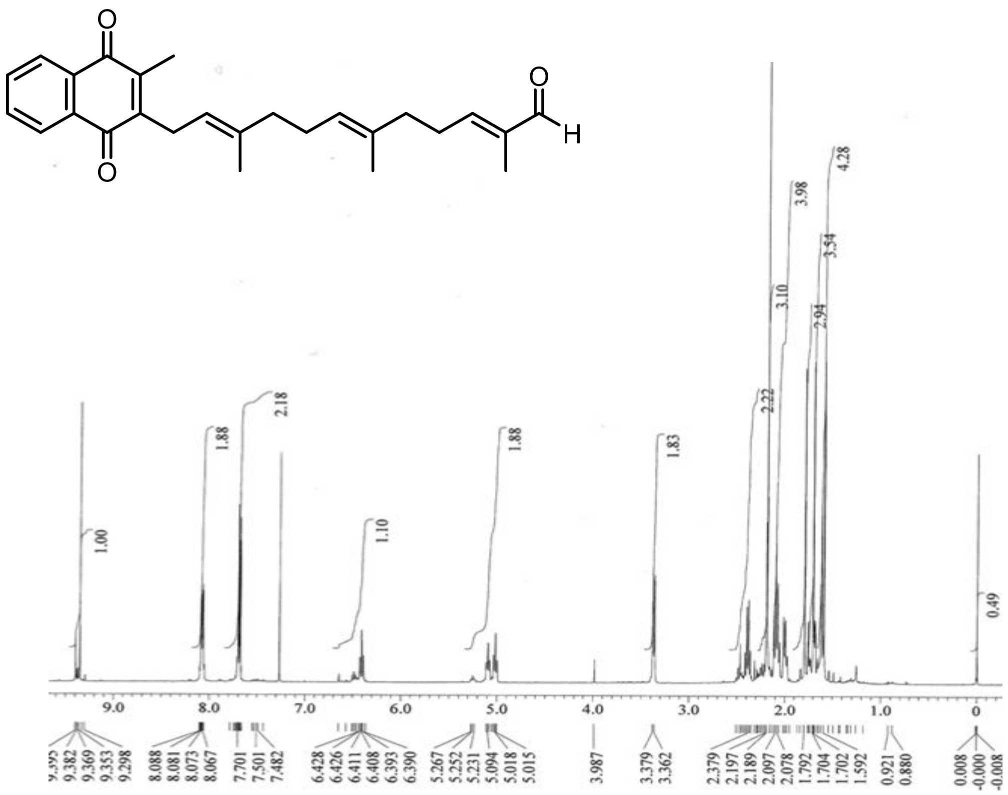

^{13}C NMR chart of compound **11**

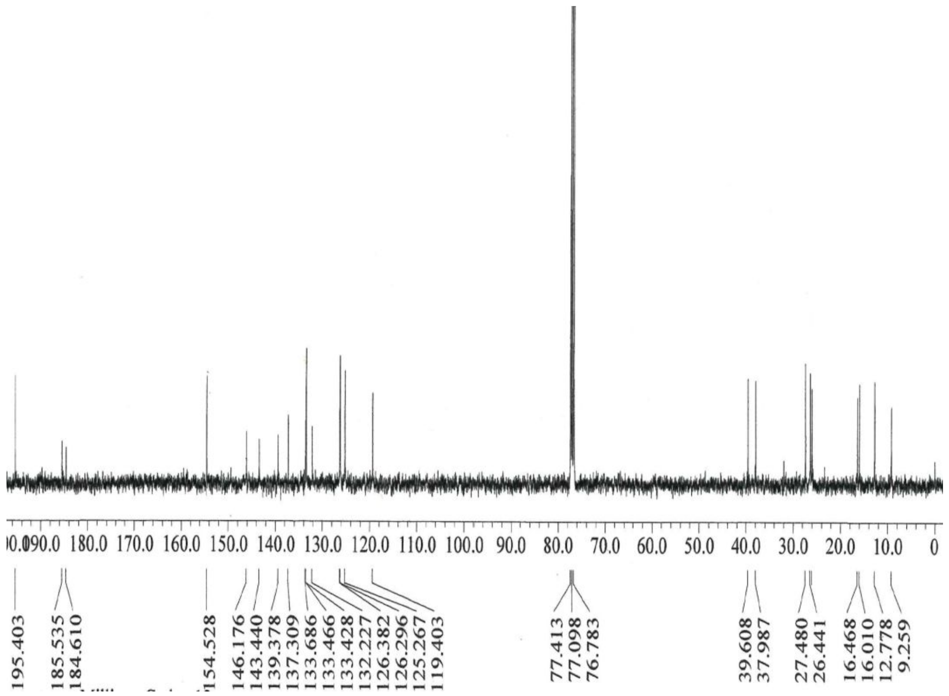

^1H NMR chart of compound **12**

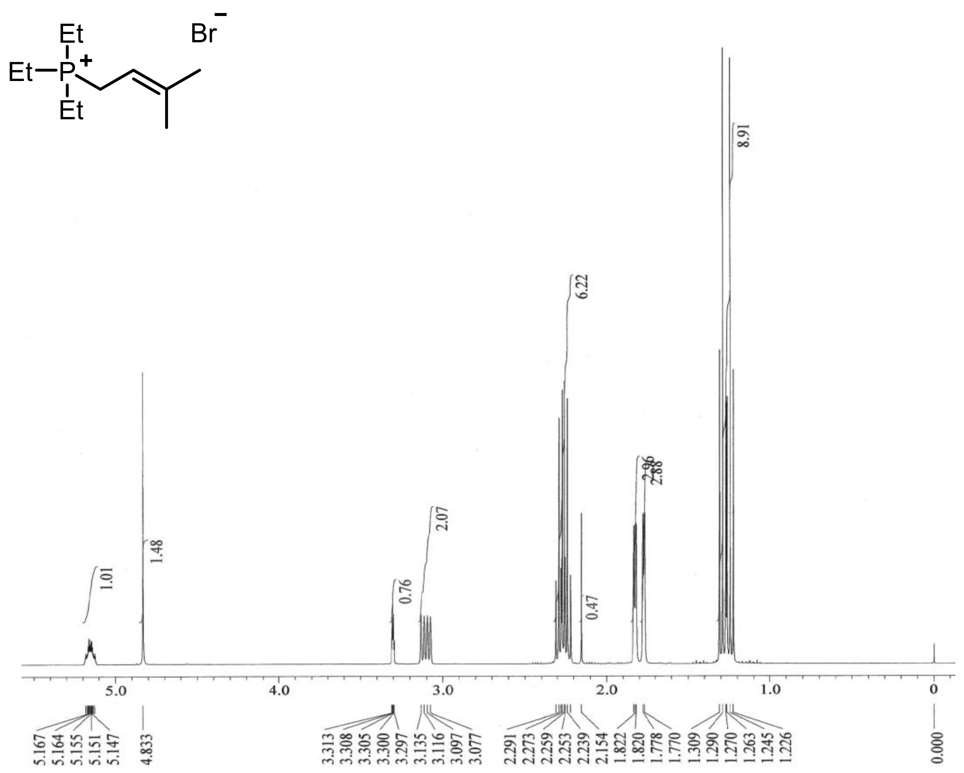

^{13}C NMR chart of compound **12**

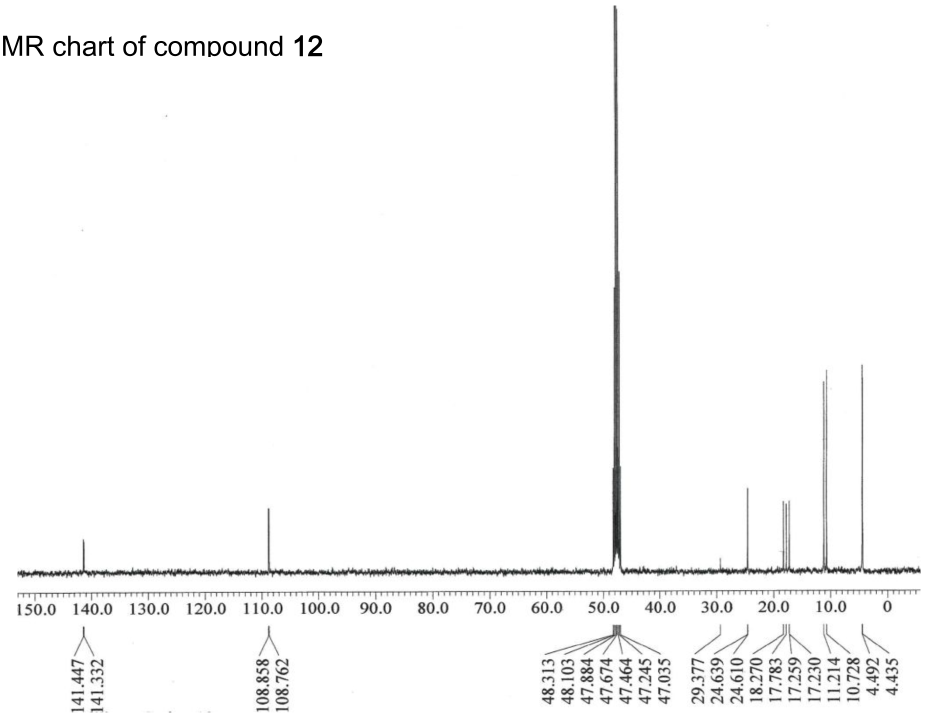

^{1}H NMR chart of compound **13**

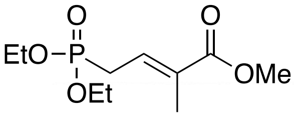

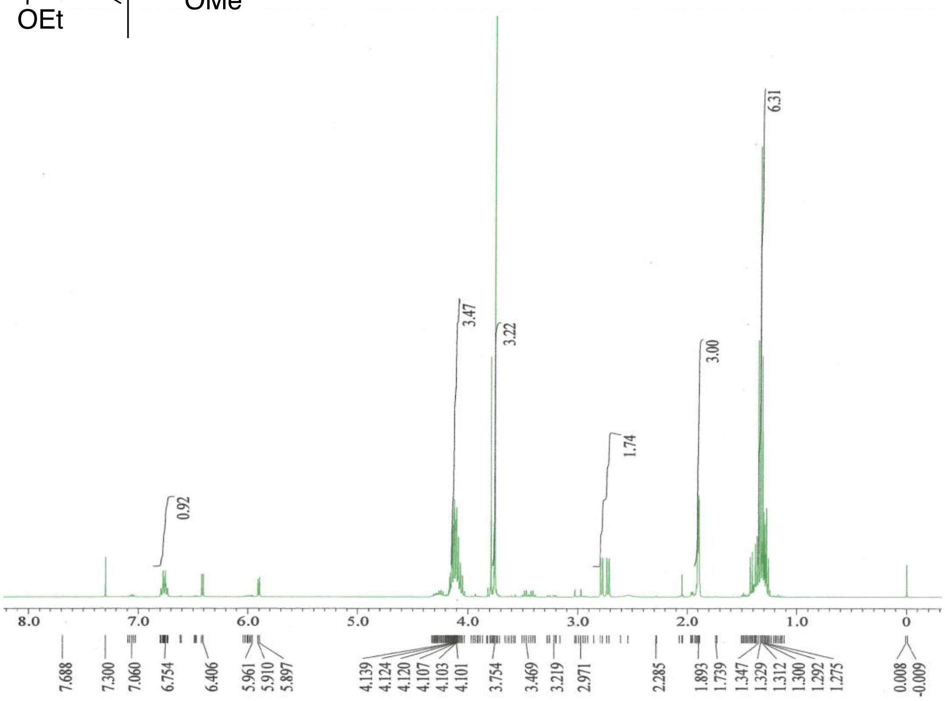

^{13}C NMR chart of compound **13**

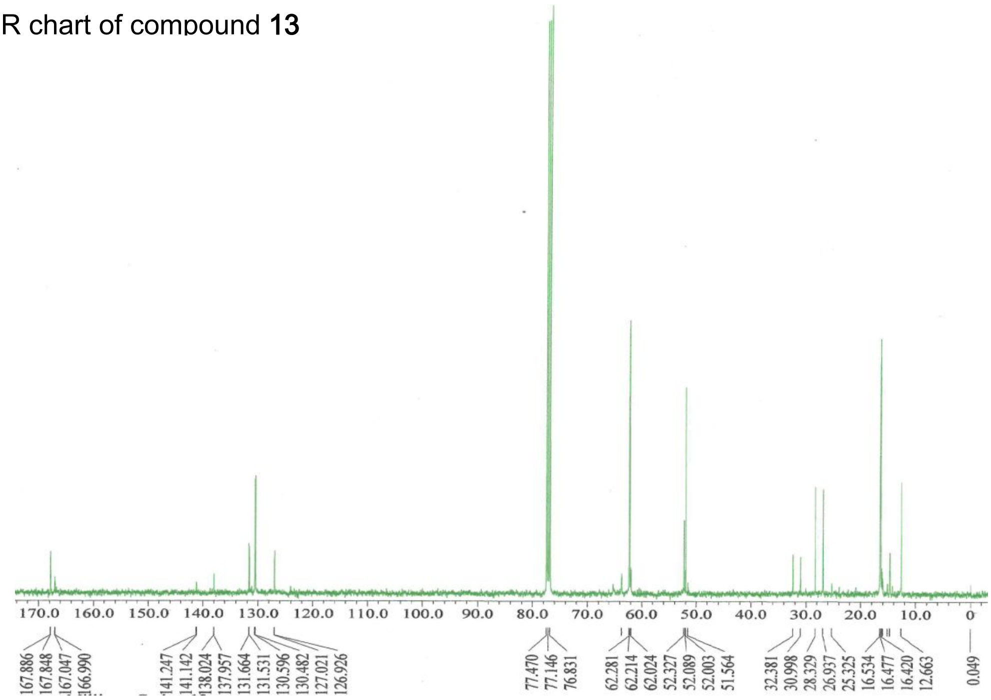

^{1}H NMR chart of compound **7**

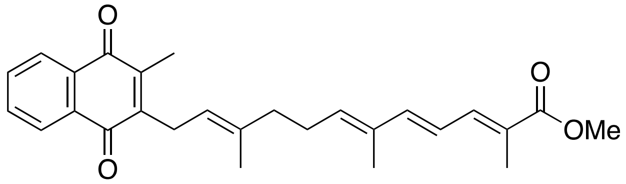

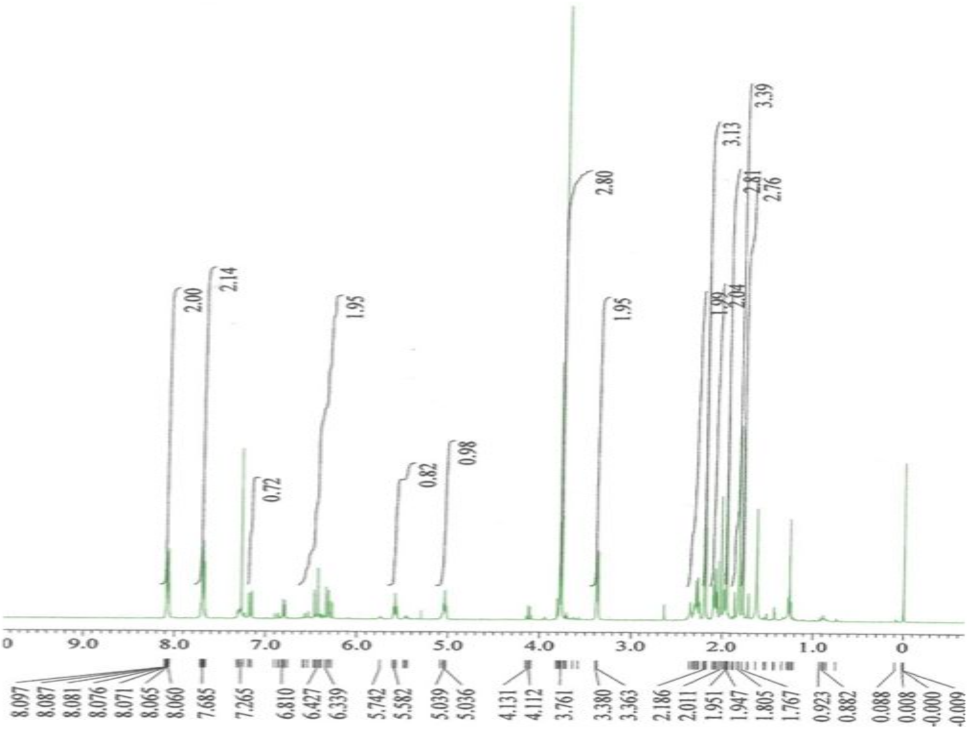

^{13}C NMR chart of compound **7**

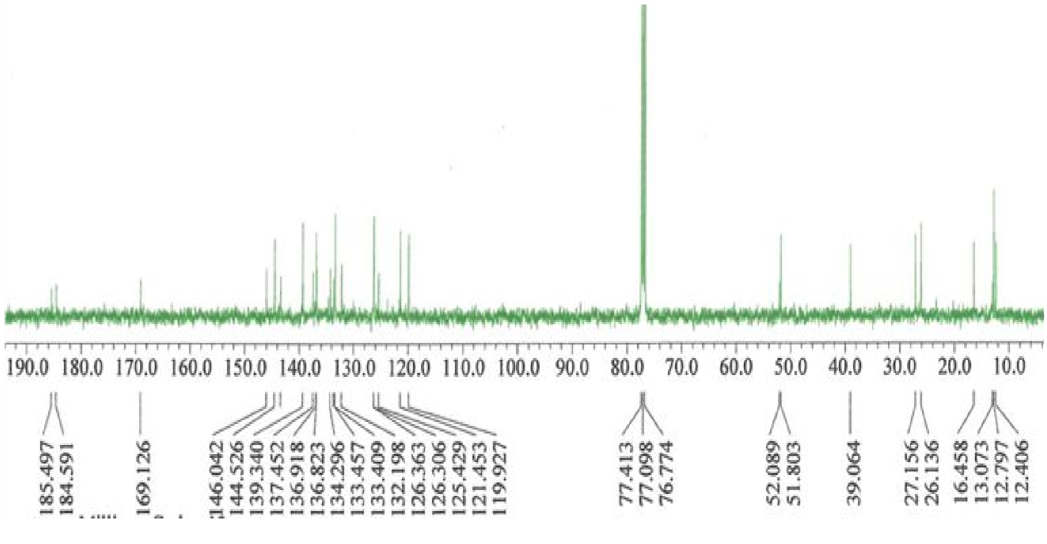

^{1}H NMR chart of compound **8**

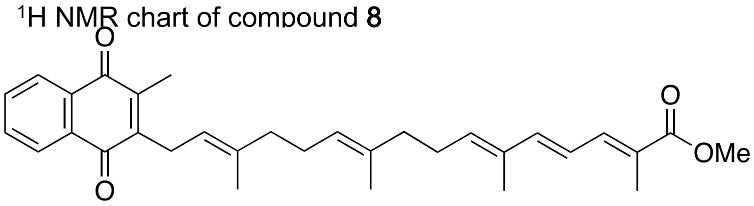

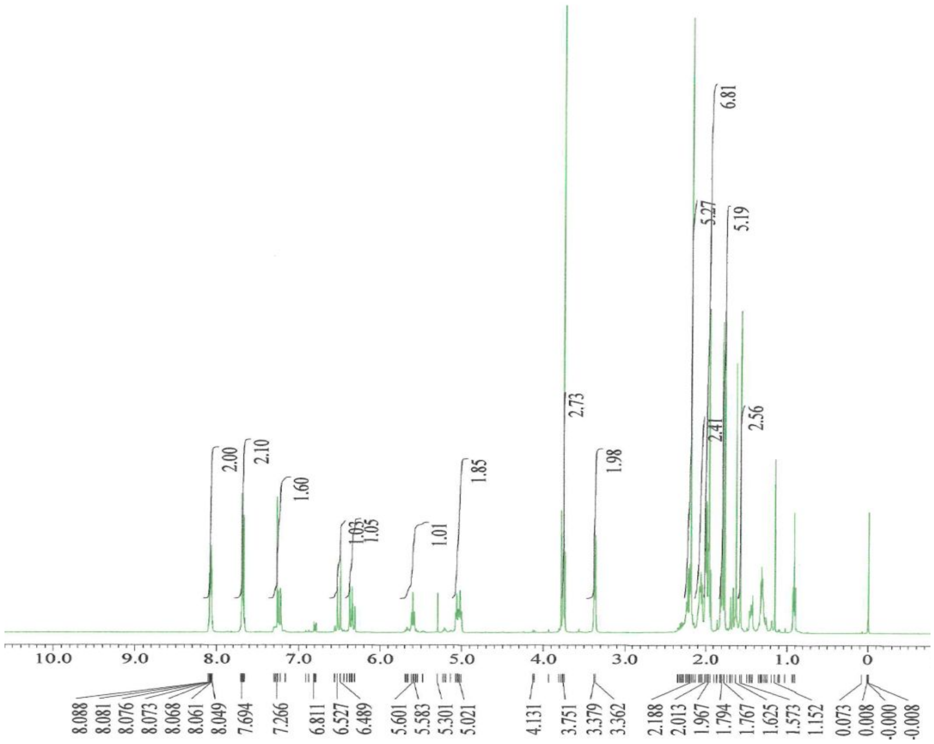

^{13}C NMR chart of compound **8**

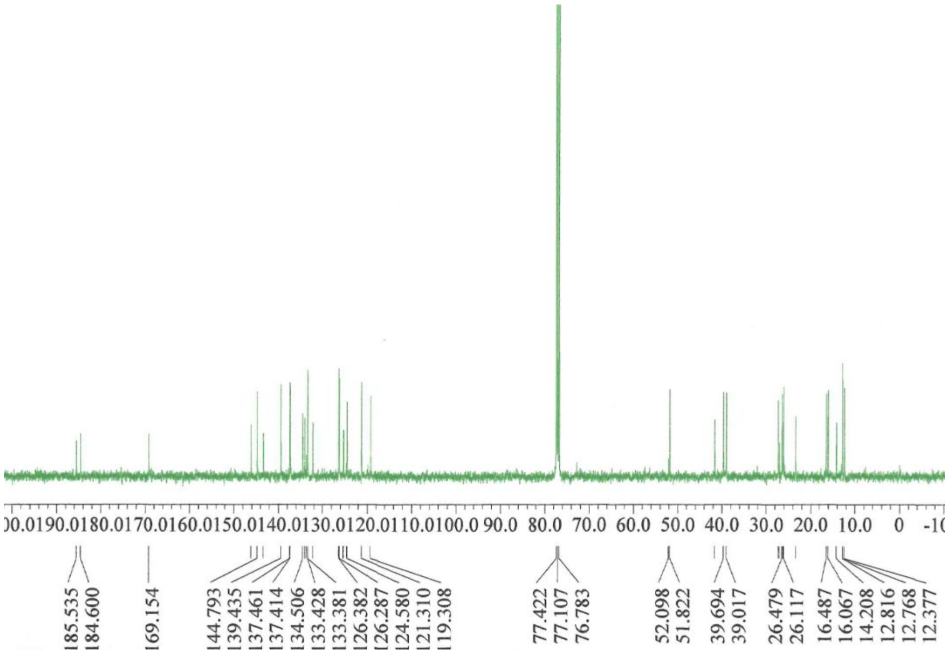

^{1}H NMR chart of compound **17**

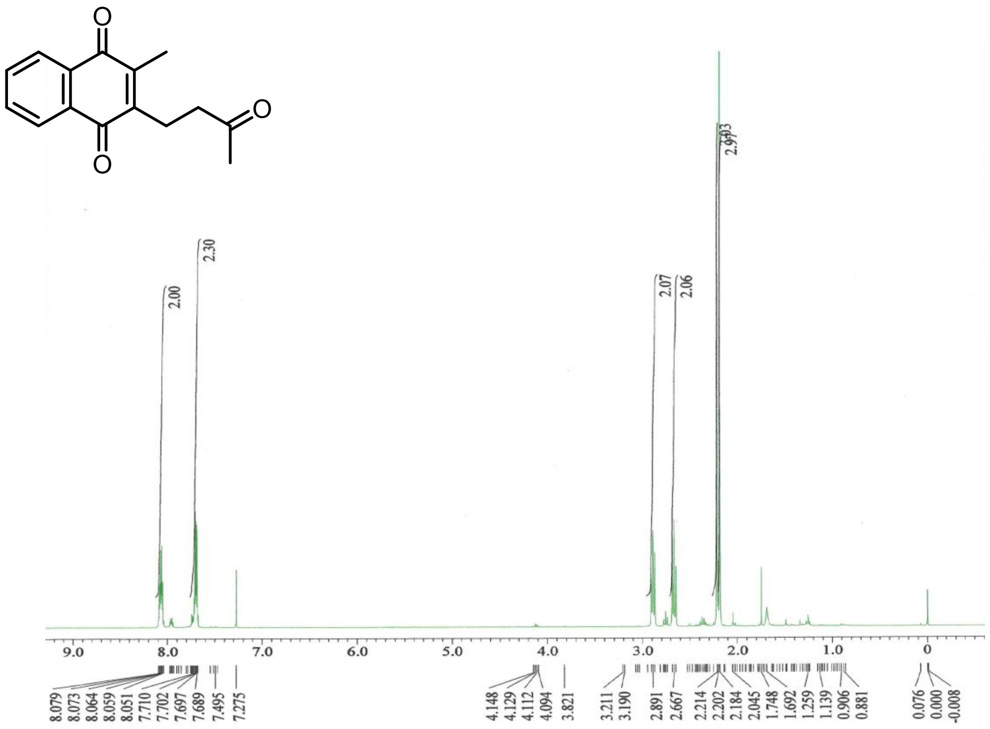

^{13}C NMR chart of compound **17**

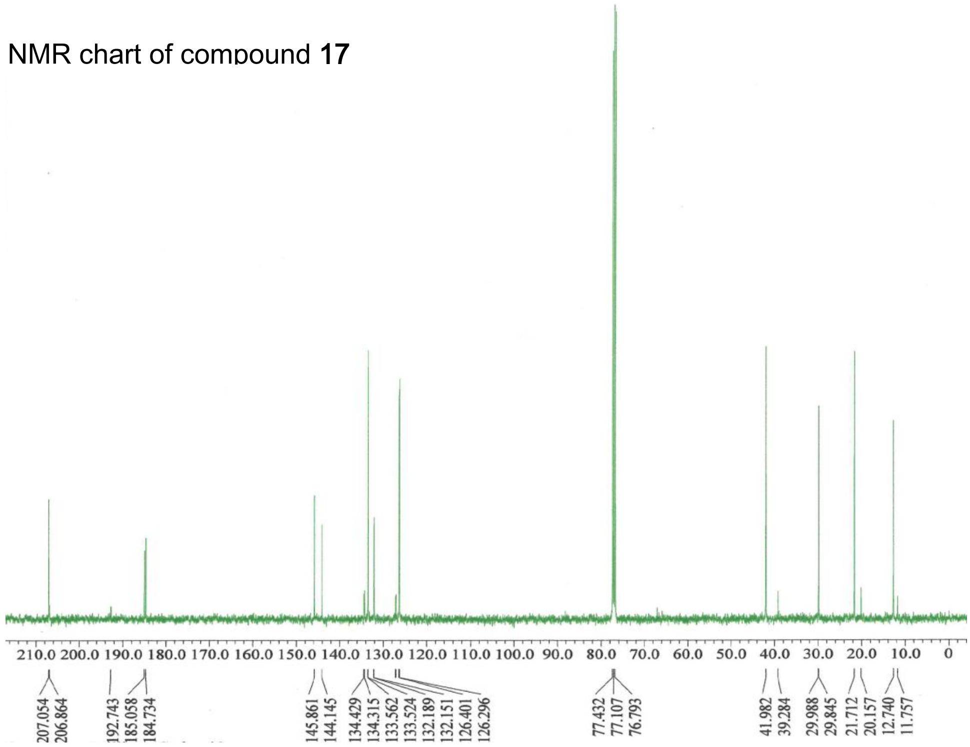

^{1}H NMR chart of compound **18**

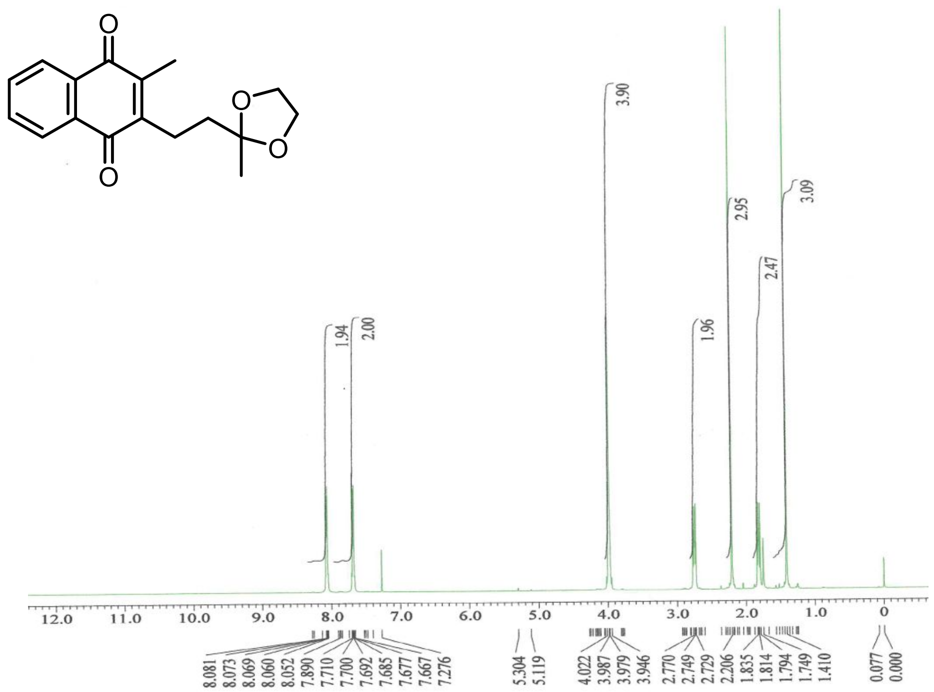

^{13}C NMR chart of compound **18**

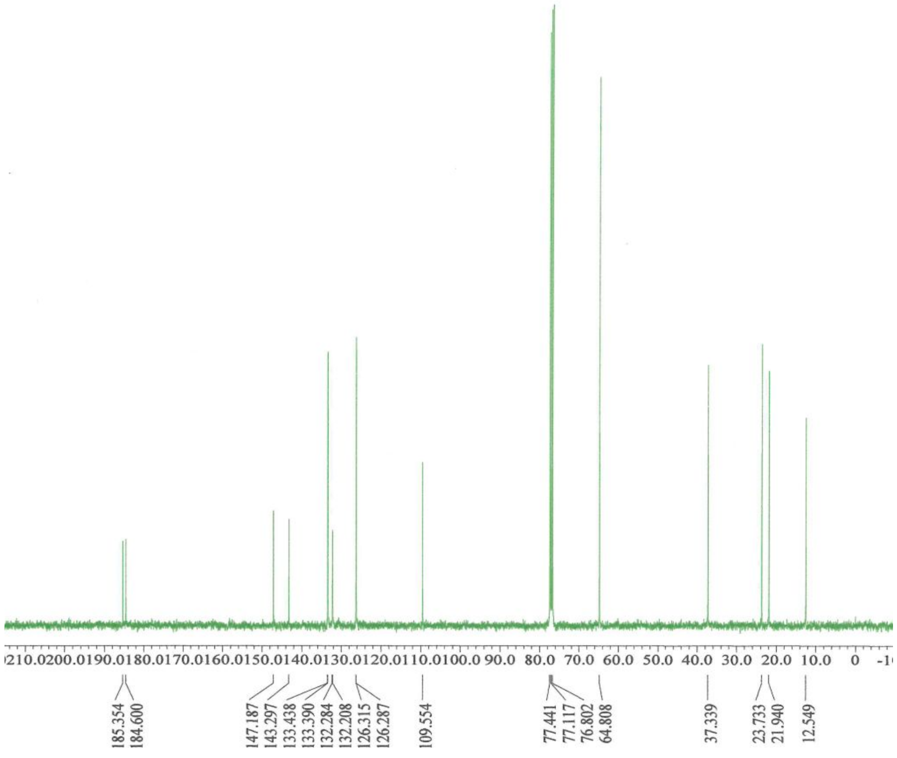

^{1}H NMR chart of compound **19**

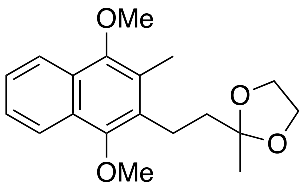

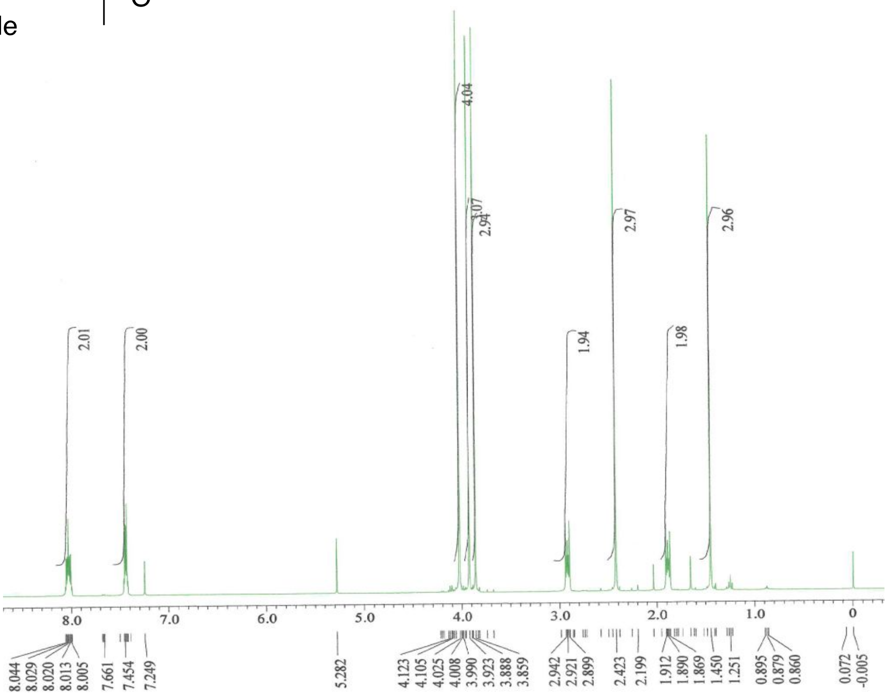

^{13}C NMR chart of compound **19**

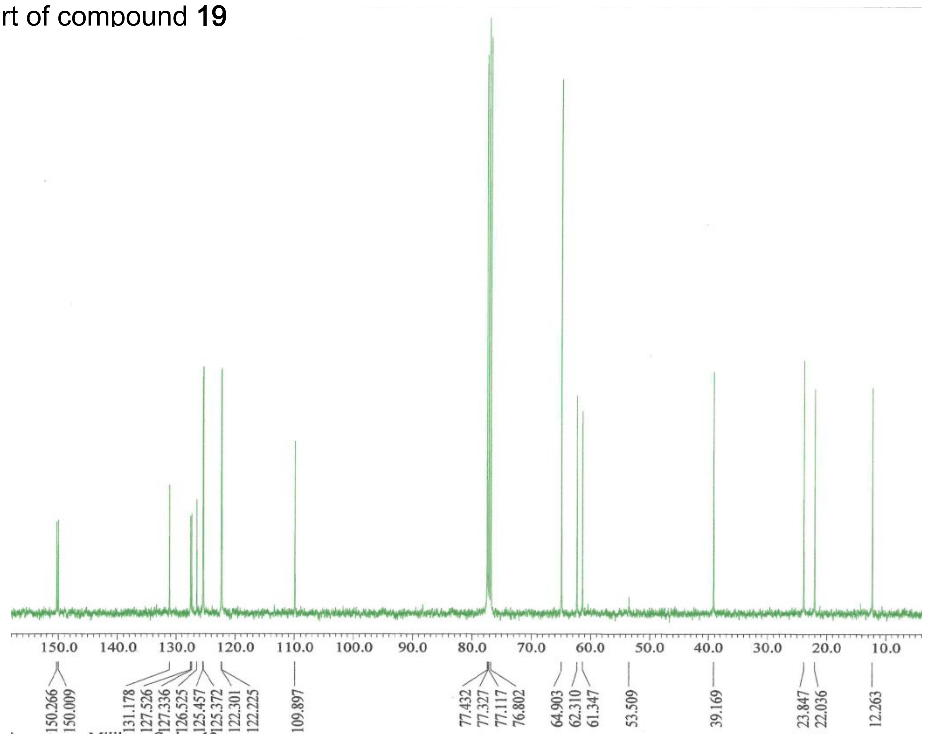

^{1}H NMR chart of compound **20**

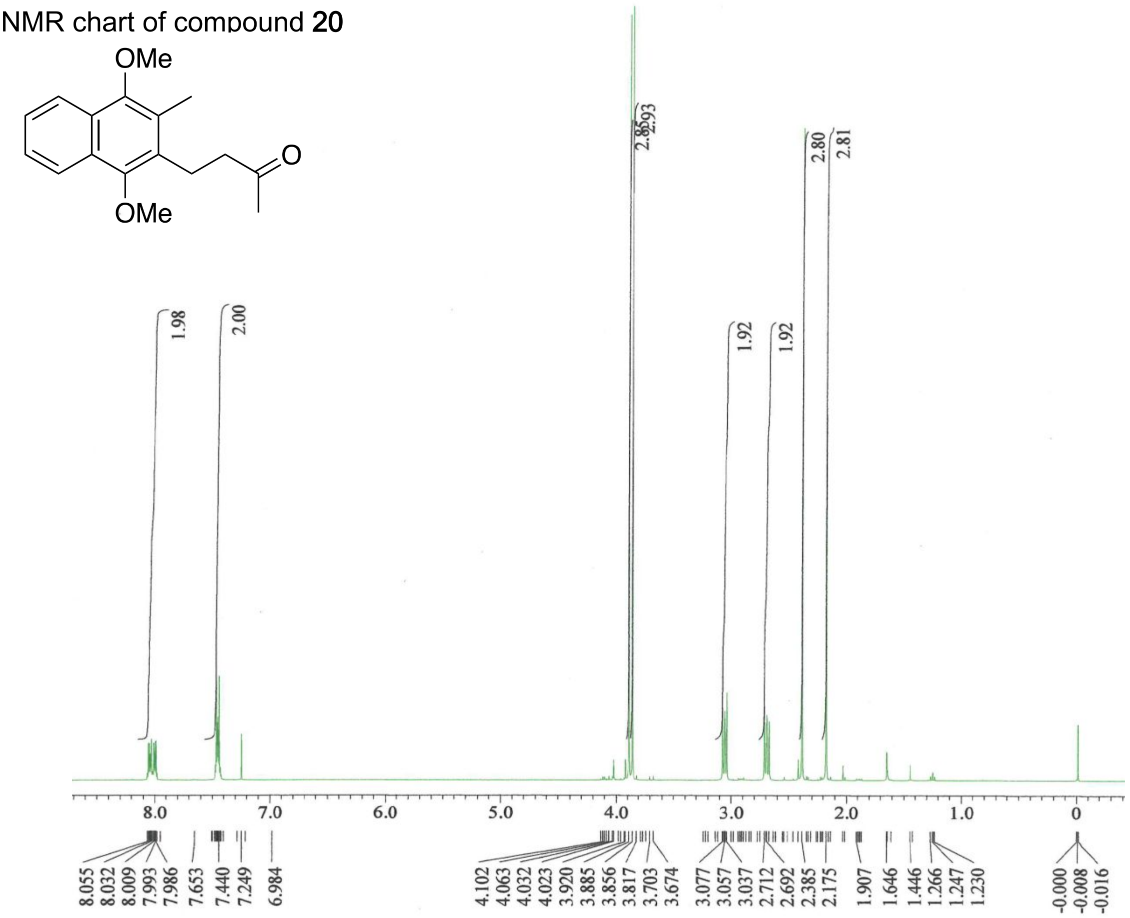

^{13}C NMR chart of compound **20**

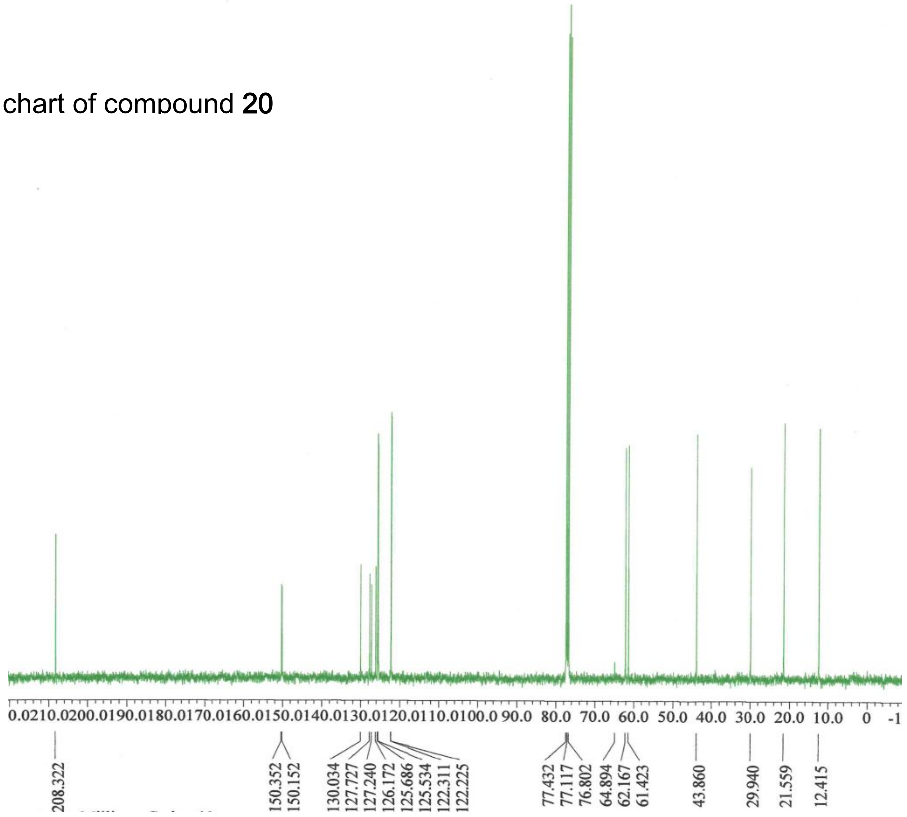

^{1}H NMR chart of compound **21**

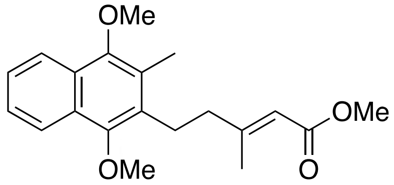

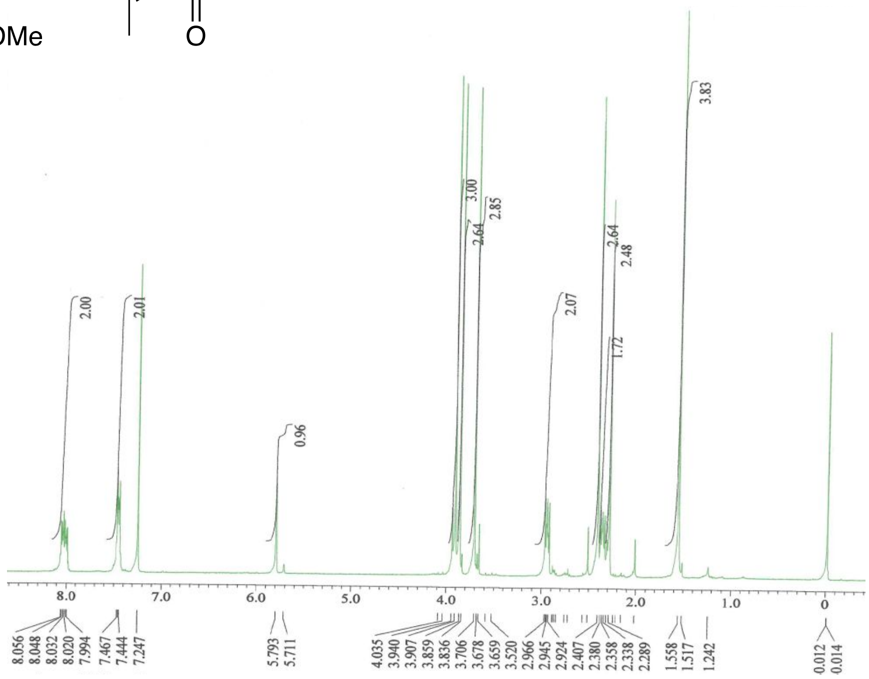

^{13}C NMR chart of compound **21**

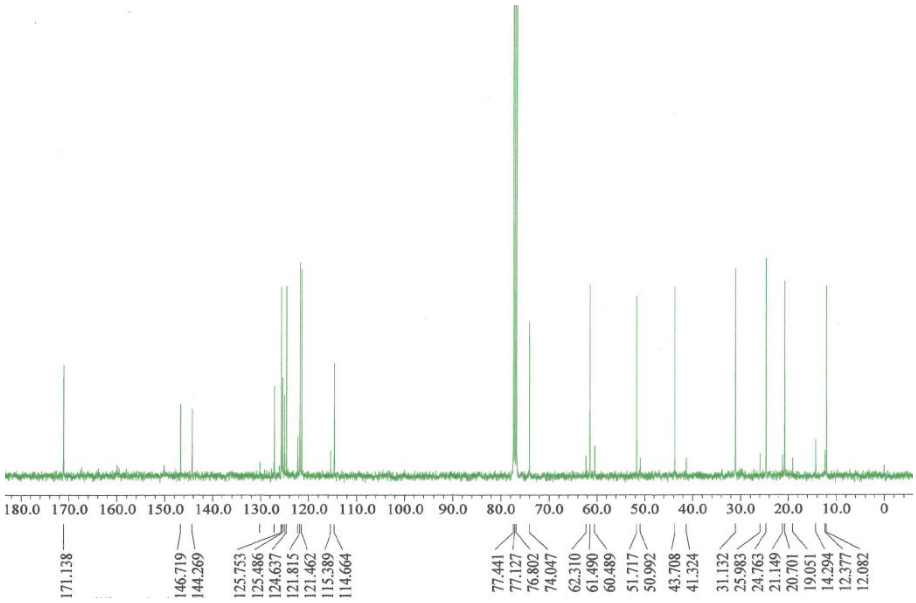

^{1}H NMR chart of compound **22**

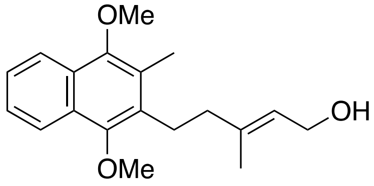

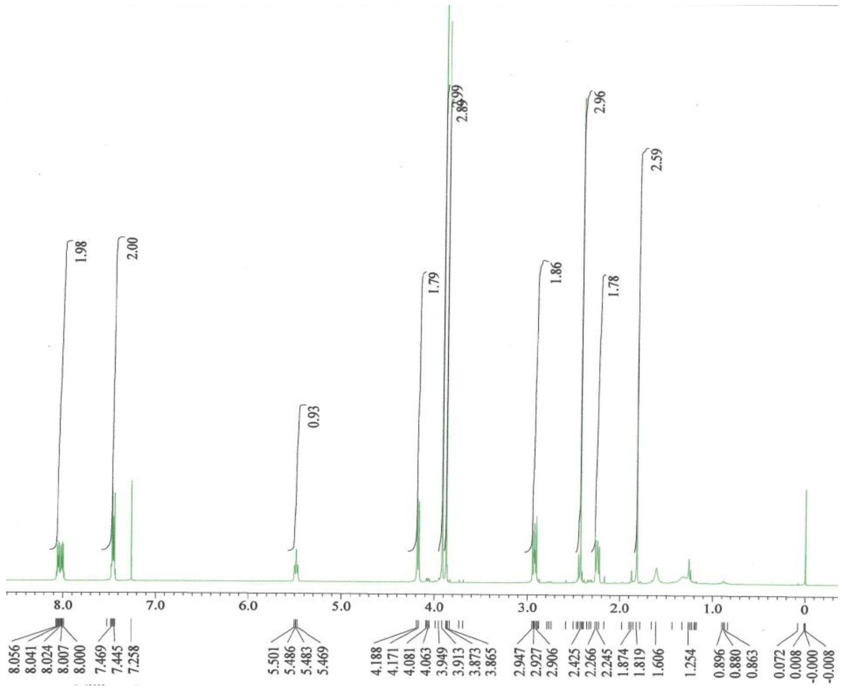

^{13}C NMR chart of compound **22**

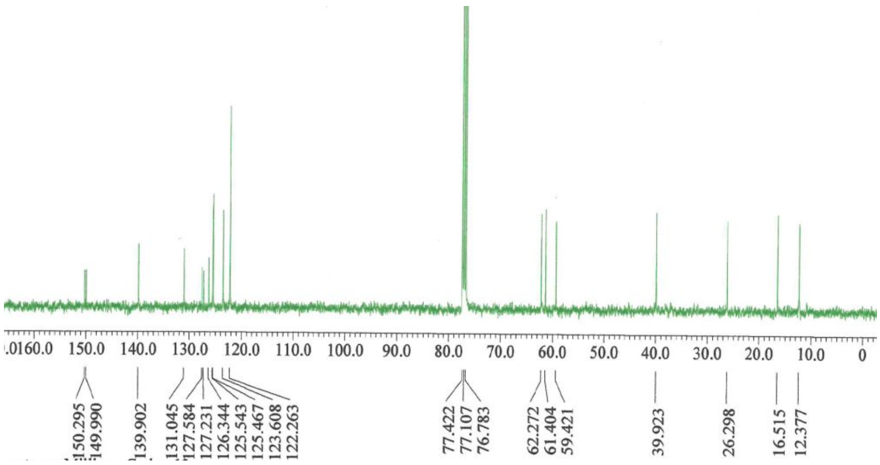

^{1}H NMR chart of compound **23**

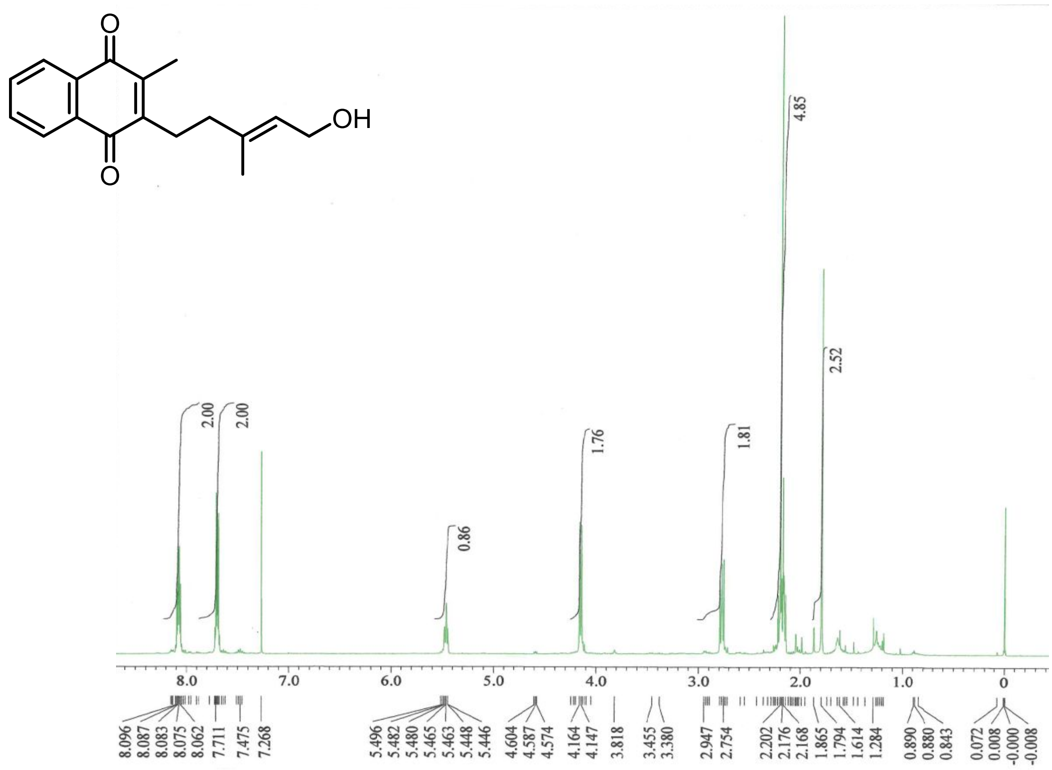

^{13}C NMR chart of compound **23**

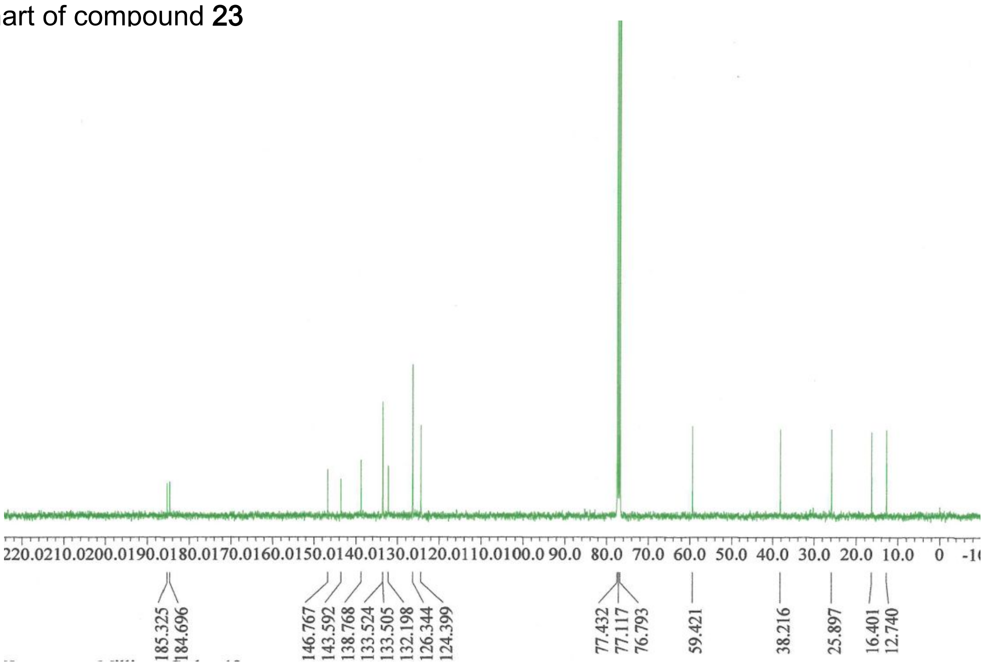

^{1}H NMR chart of compound **24**

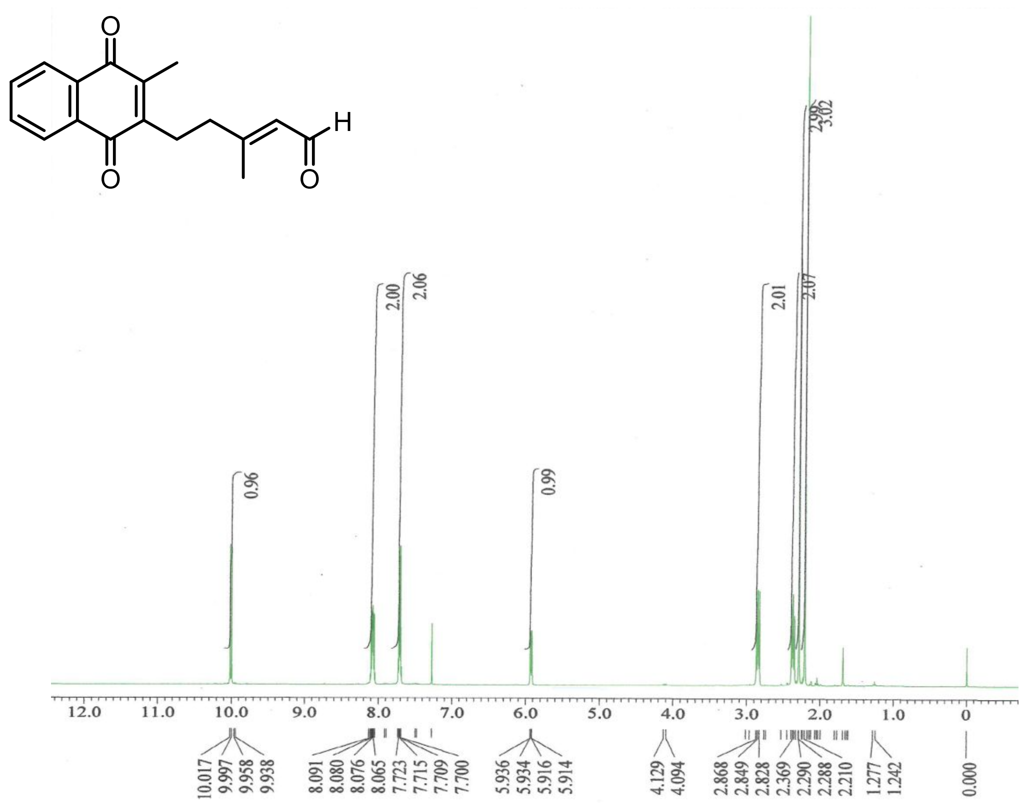

^{13}C NMR chart of compound **24**

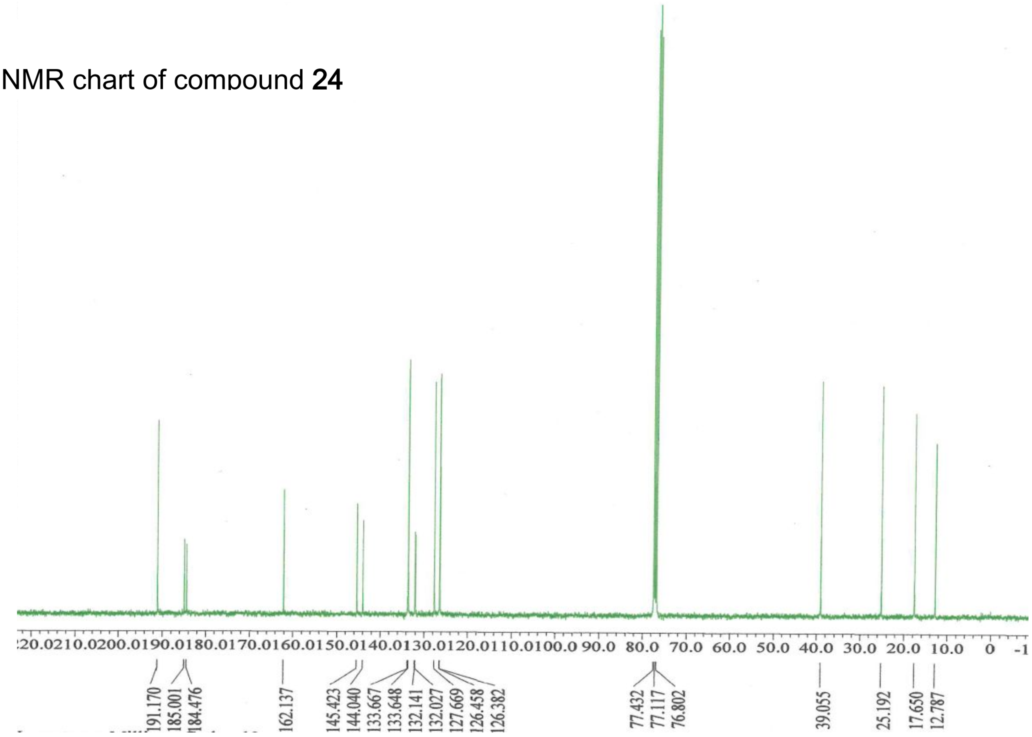

^1H NMR chart of compound **25**

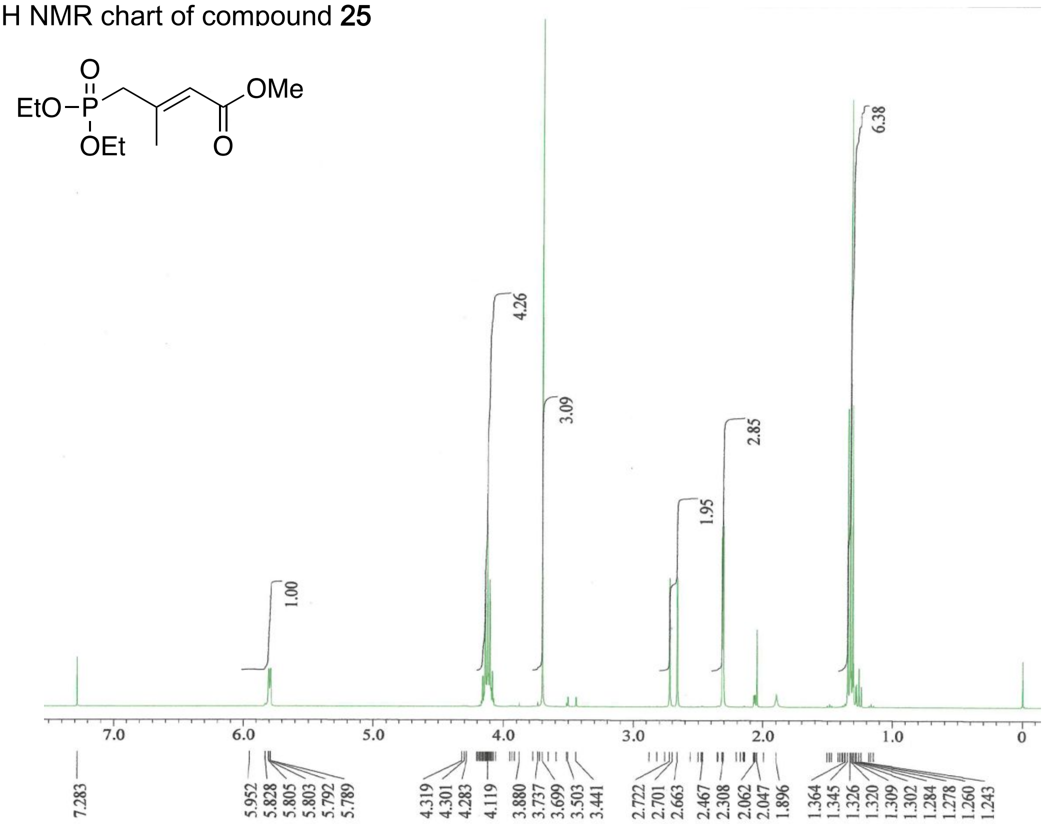

^{13}C NMR chart of compound **25**

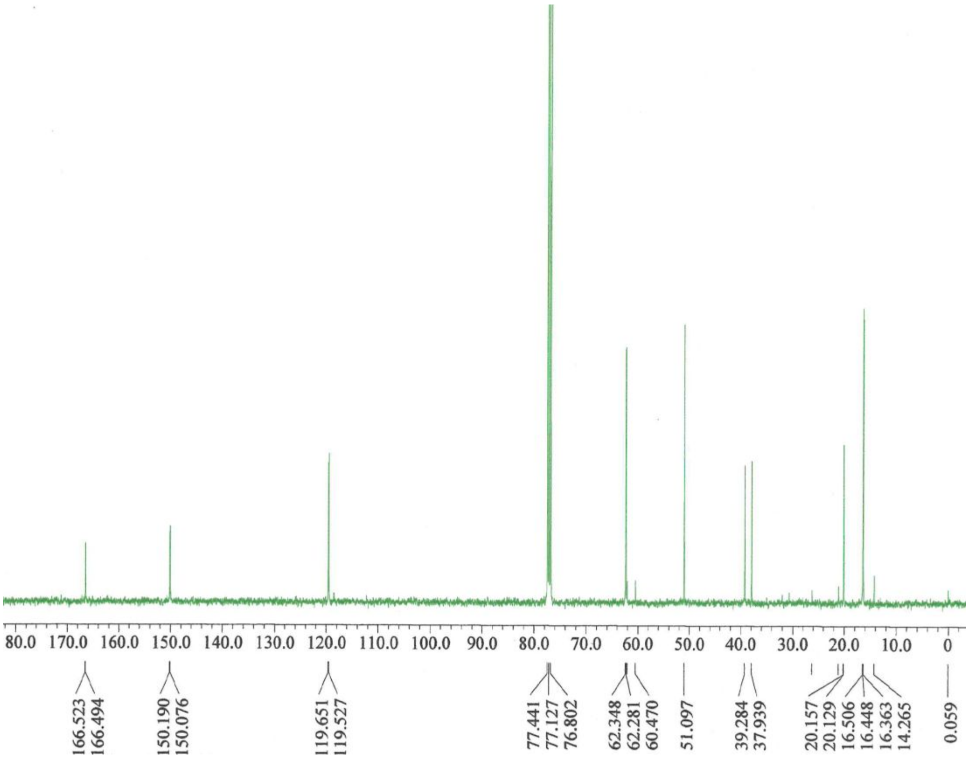

^{1}H NMR chart of compound **9**

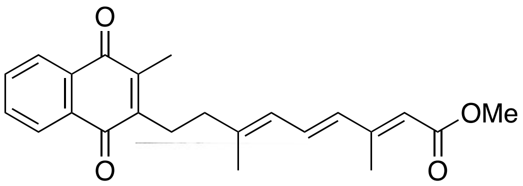

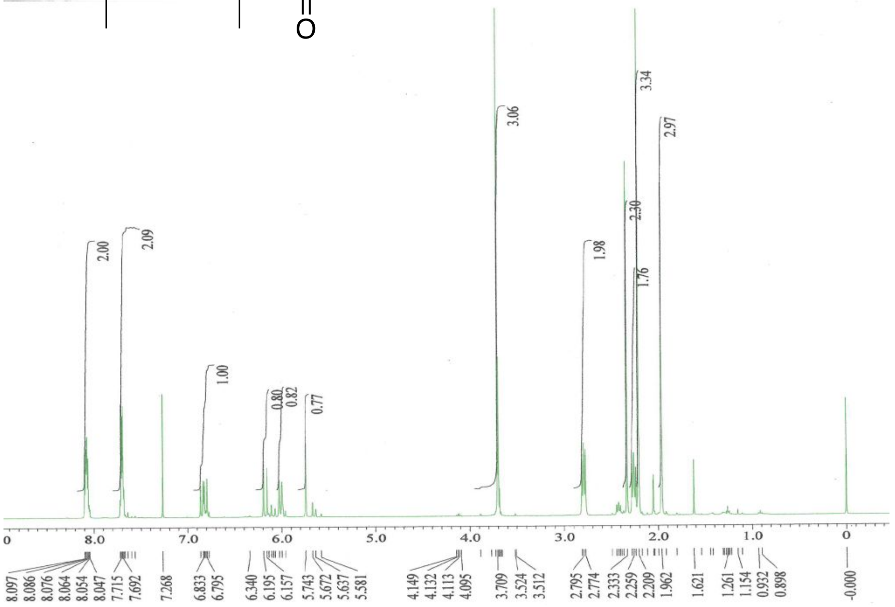

^{13}C NMR chart of compound **9**

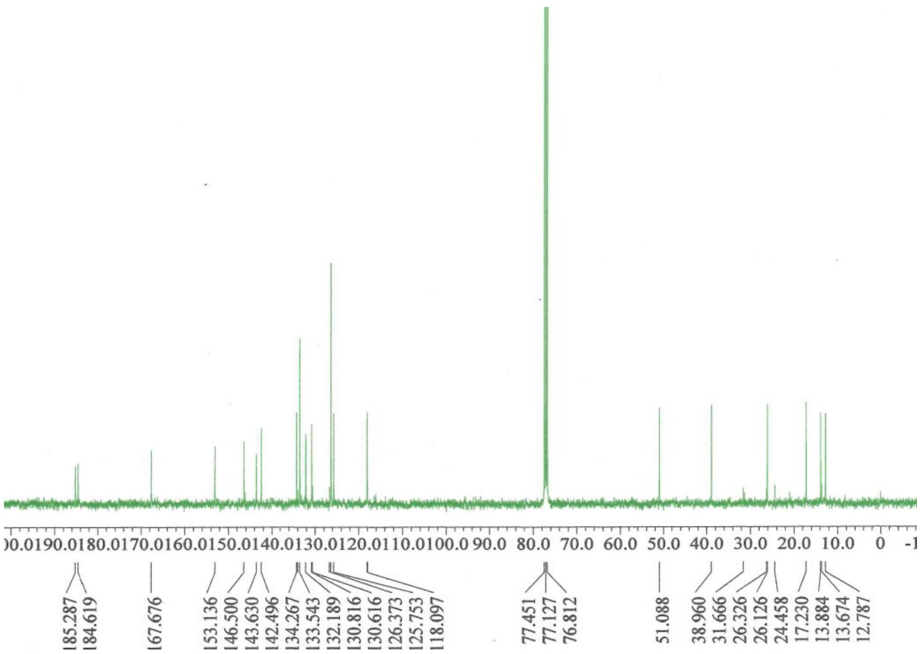

^{1}H NMR chart of compound **27**

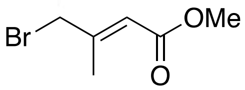

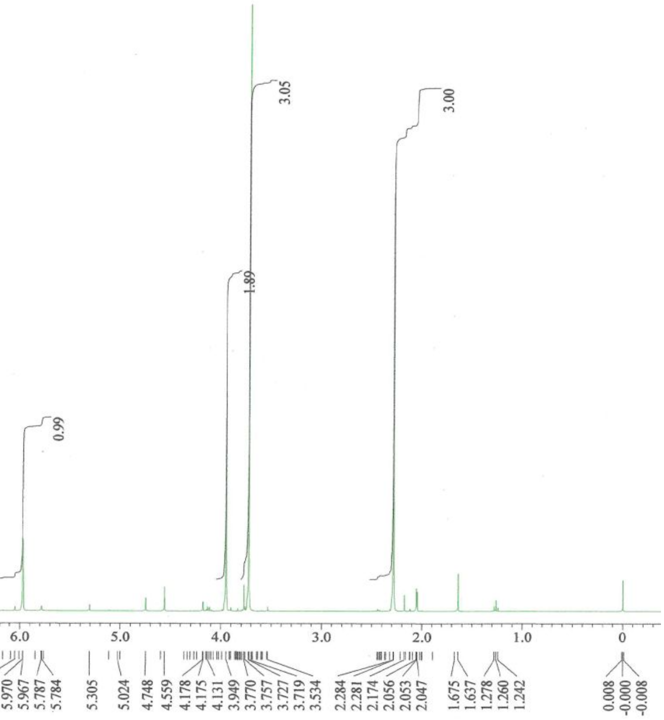

^{13}C NMR chart of compound **27**

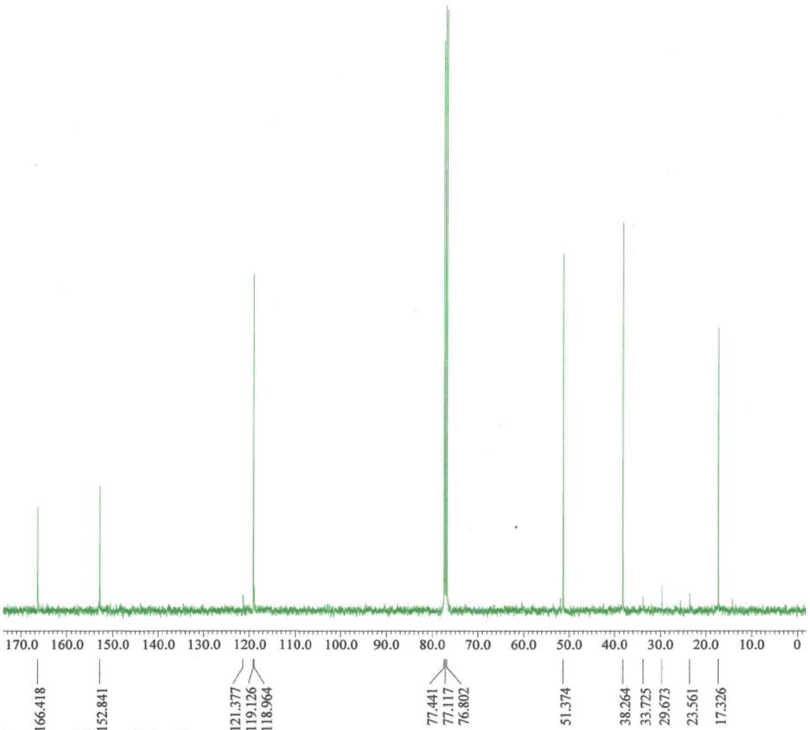

II. HPLC data of compounds 3 – 9 used on biological assays

The purity of compounds **3** – **9** was confirmed by following method. The HPLC analyses were conducted with a Shimadzu HPLC system (Simadzu, Kyoto, Japan) consisting of a binary pump (LC-10ADvp liquid chromatography), an automatic solvent degasser (DGU-12A degasser), and a manualinjector. Separations were carried out using a reversed-phase C_{18} analytical column (COSMOSIL 5C_{18}-AR-II; 4.6 mm i.d. × 250 mm) (Nakalai tesque, Kyoto, Japan) with a solvent system consisting of an isocratic solvent. The solvent contained either methanol, ethanol, or water was delivered at 1.0 mL/min. This mobile phase was passed through the column at 1.0 mL/min. The column was maintained at 40 °C with a column oven (CTO-10ACvp column oven). Vitamin K analogues were detected at 258 nm with an SPD-10Avp UV-VIS detector.

Compound **3**

Eluant:EtOH, Flow Rate: 1.0 mL / min,

Detection: Ch.1 254 nm, Retention Time: 3.473 min

Purity: 95%

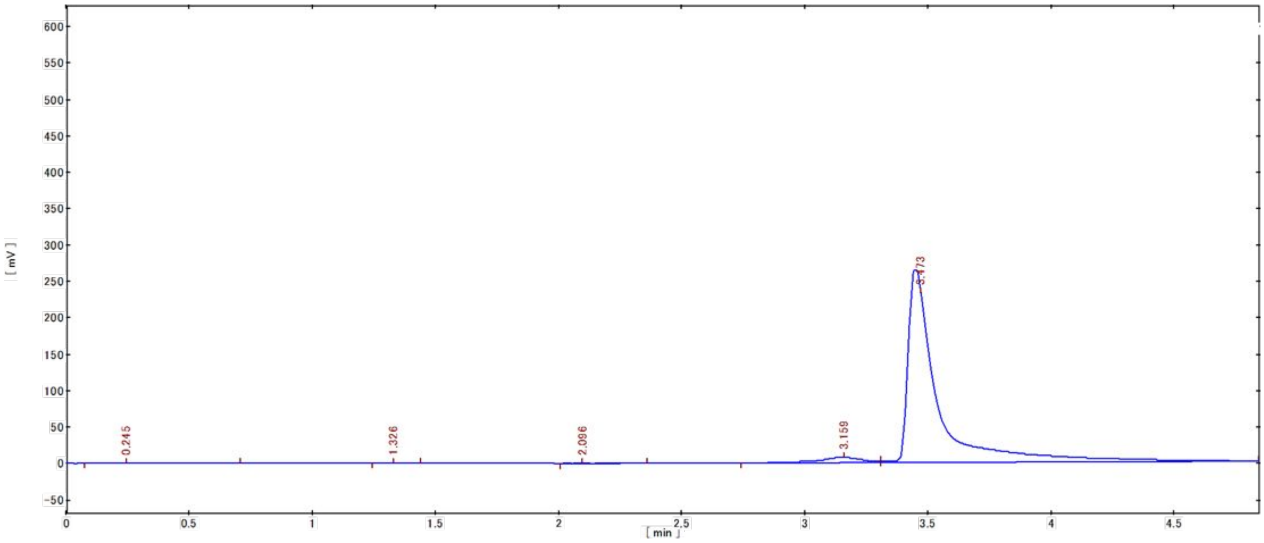

Compound **4**

Eluant:EtOH, Flow Rate: 1.0 mL / min,

Detection: Ch.1 254 nm, Retention Time: 4.192 min

Purity: 97%

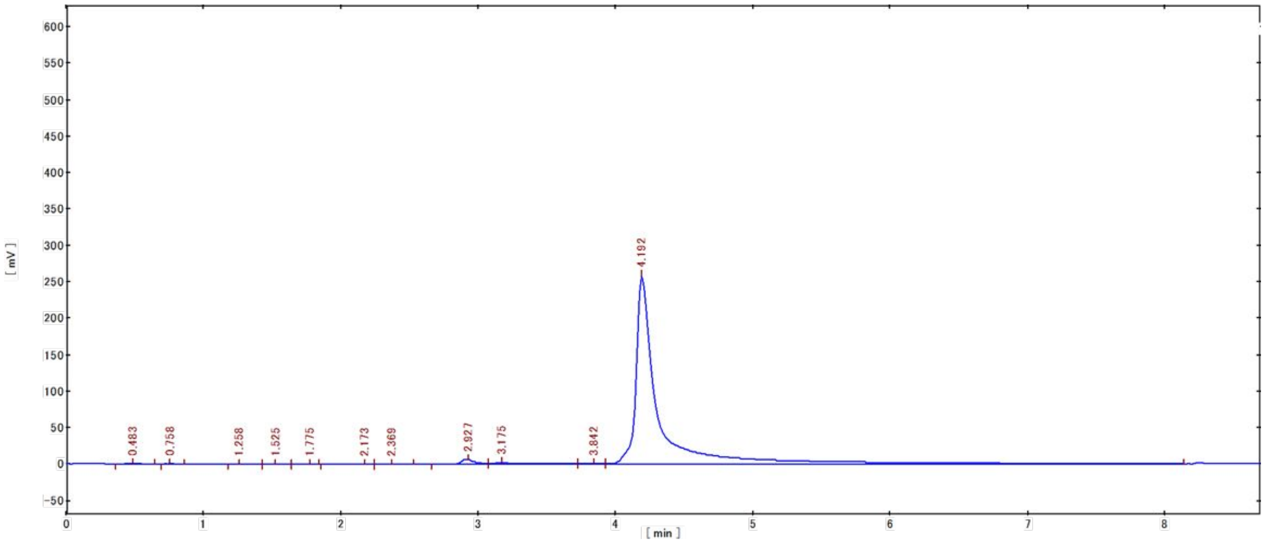

Compound **5**

Eluant:EtOH, Flow Rate: 1.0 mL / min,

Detection: Ch.1 254 nm, Retention Time: 3.953 min

Purity: 99%

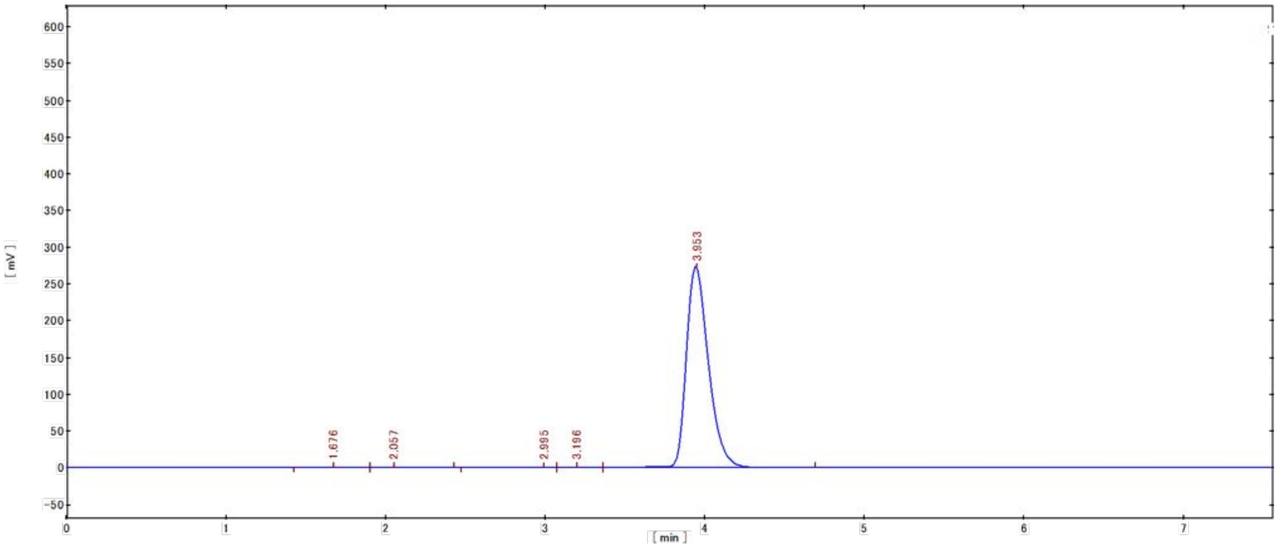

Compound **6**

Eluant:EtOH, Flow Rate: 1.0 mL / min,

Detection: Ch.1 254 nm, Retention Time: 2.605 min

Purity: 99%

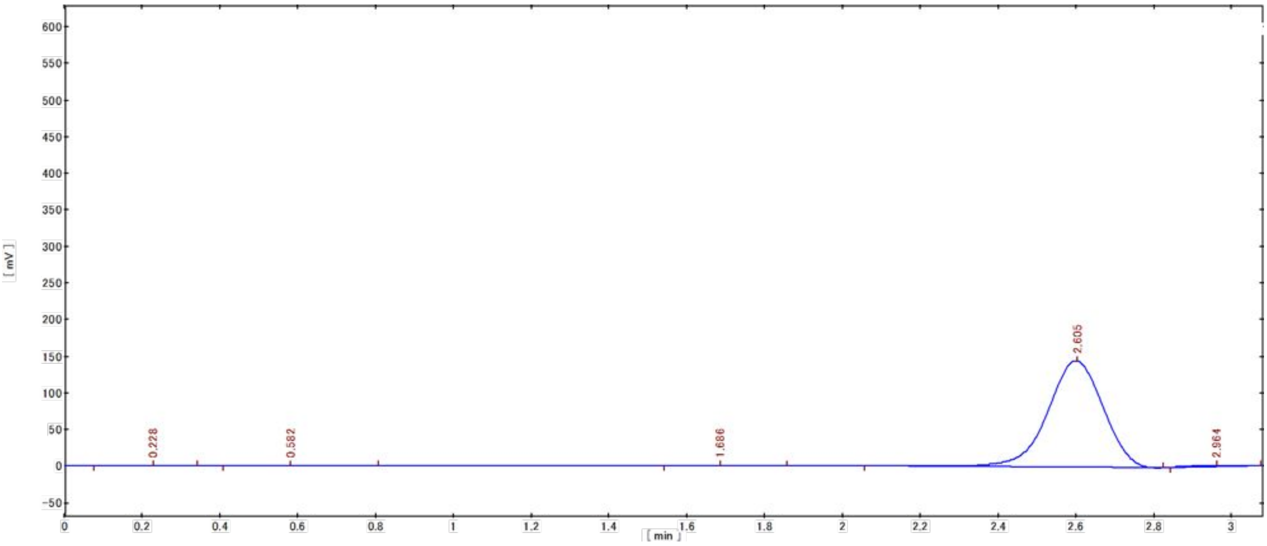

Compound **7**

Eluant:EtOH, Flow Rate: 1.0 mL / min,

Detection: Ch.1 254 nm, Retention Time: 2.605 min

Purity: 98%

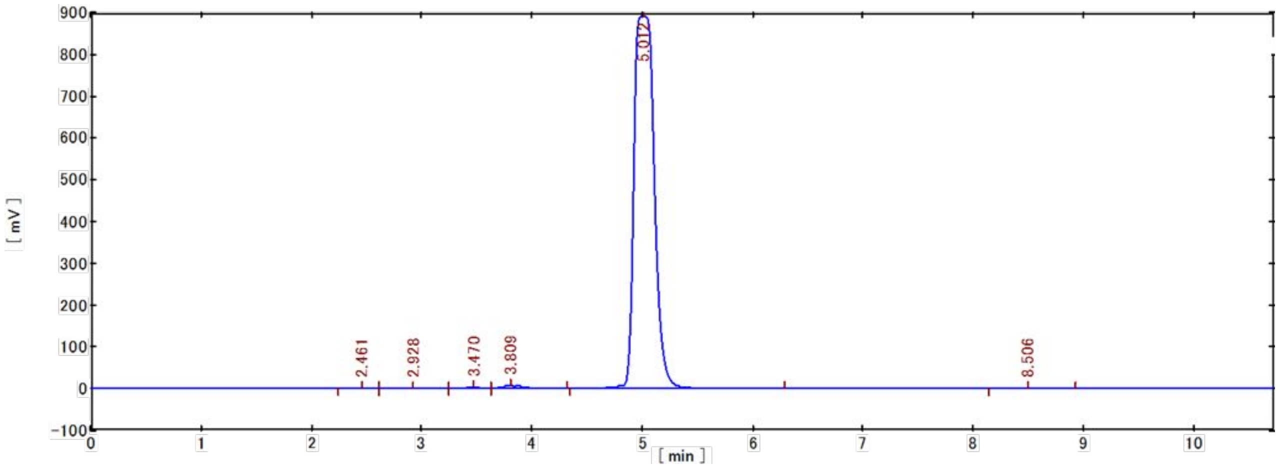

Compound **8**

Eluant:EtOH, Flow Rate: 1.0 mL / min,

Detection: Ch.1 254 nm, Retention Time: 6.728 min

Purity: 96%

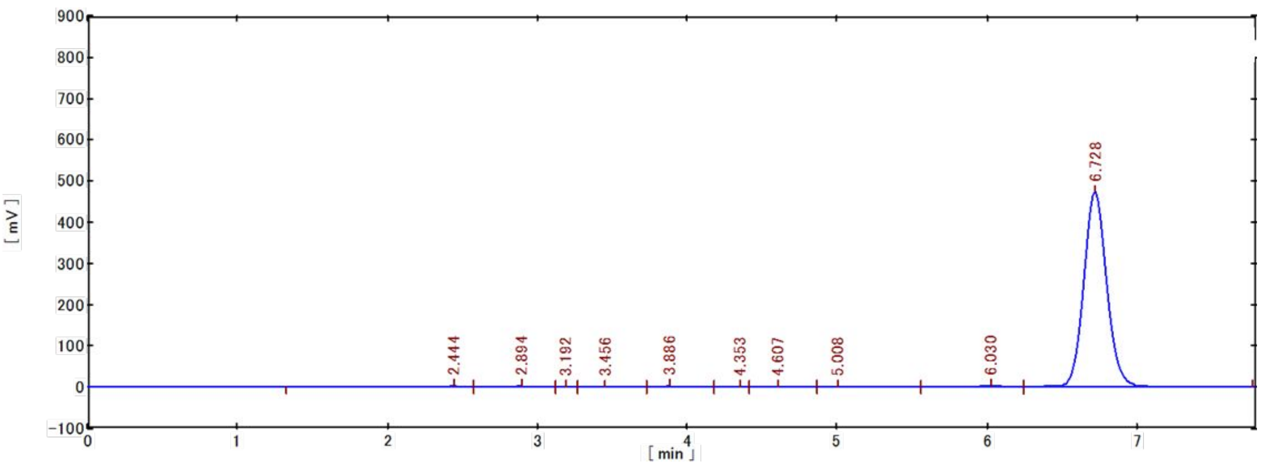

Compound **9**

Eluant:Acetonitrile, Flow Rate: 1.0 mL / min,

Detection: Ch.1 254 nm, Retention Time: 3.674 min

Purity: 93%

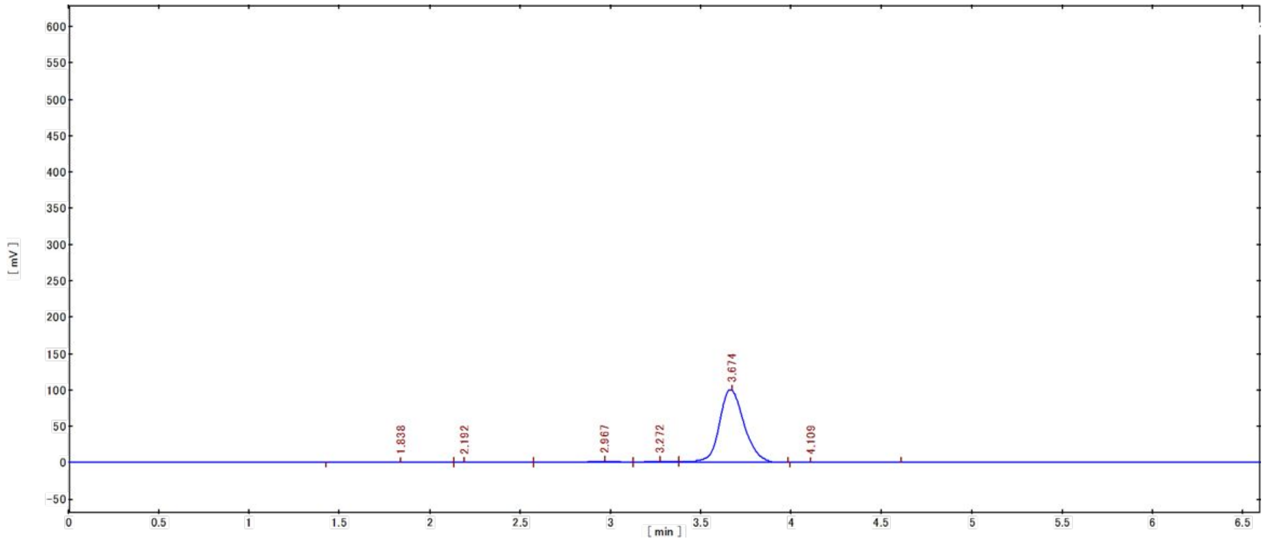

III.Supplementary Fig.1 – Fig.7

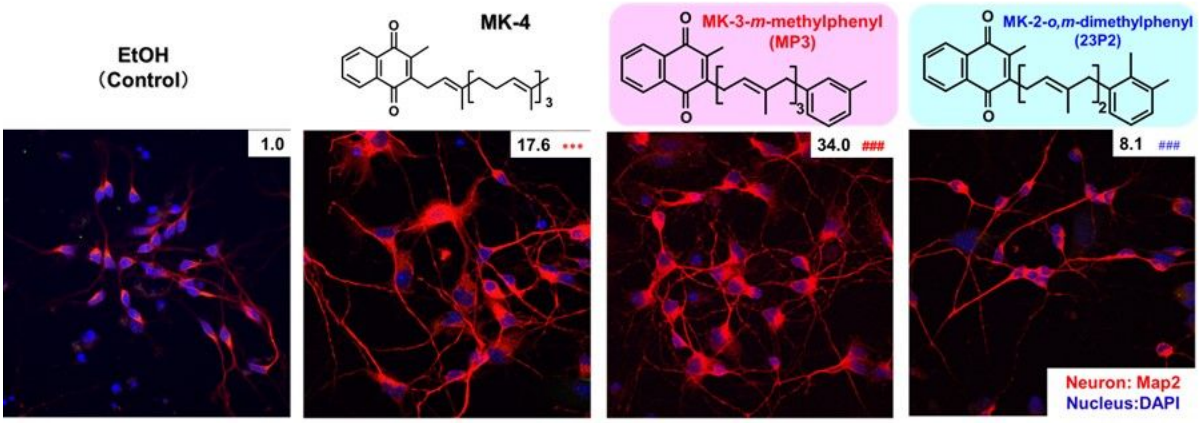

Supplementary Fig. 1. Structural formulae of MK-4, MP3, and 23P2, and their actions on the differentiation of neural stem cells into neurons. Neural stem cells obtained from mouse embryos were treated with EtOH (EtOH content in the medium was 0.1%) as a negative control, MK-4 as a positive control, MK-3-*m*-methylphenyl (MP3) as a compound that promotes neural stem cell differentiation into neurons, and MK-2-*o*,*m*-dimethylphenyl (23P2) as a compound that suppresses neural stem cell differentiation into neurons. After the treatment, the cells were immunostained for microtubule-associated protein 2 (Map2) and images of the cells were collected. Then, four areas were randomly selected in each immunostained image and the number of Map2-positive neurons were quantified and averaged across the four areas; the relative value of Map2-positive neurons (red) in cells treated with EtOH is shown in the figure are presented in the top-right corner of each image. Significant differences were assessed compared with EtOH ($^{***}p < 0.001$) or MK-4 treatment ($^{\#\#\#}p < 0.001$) (Dunnet's test).

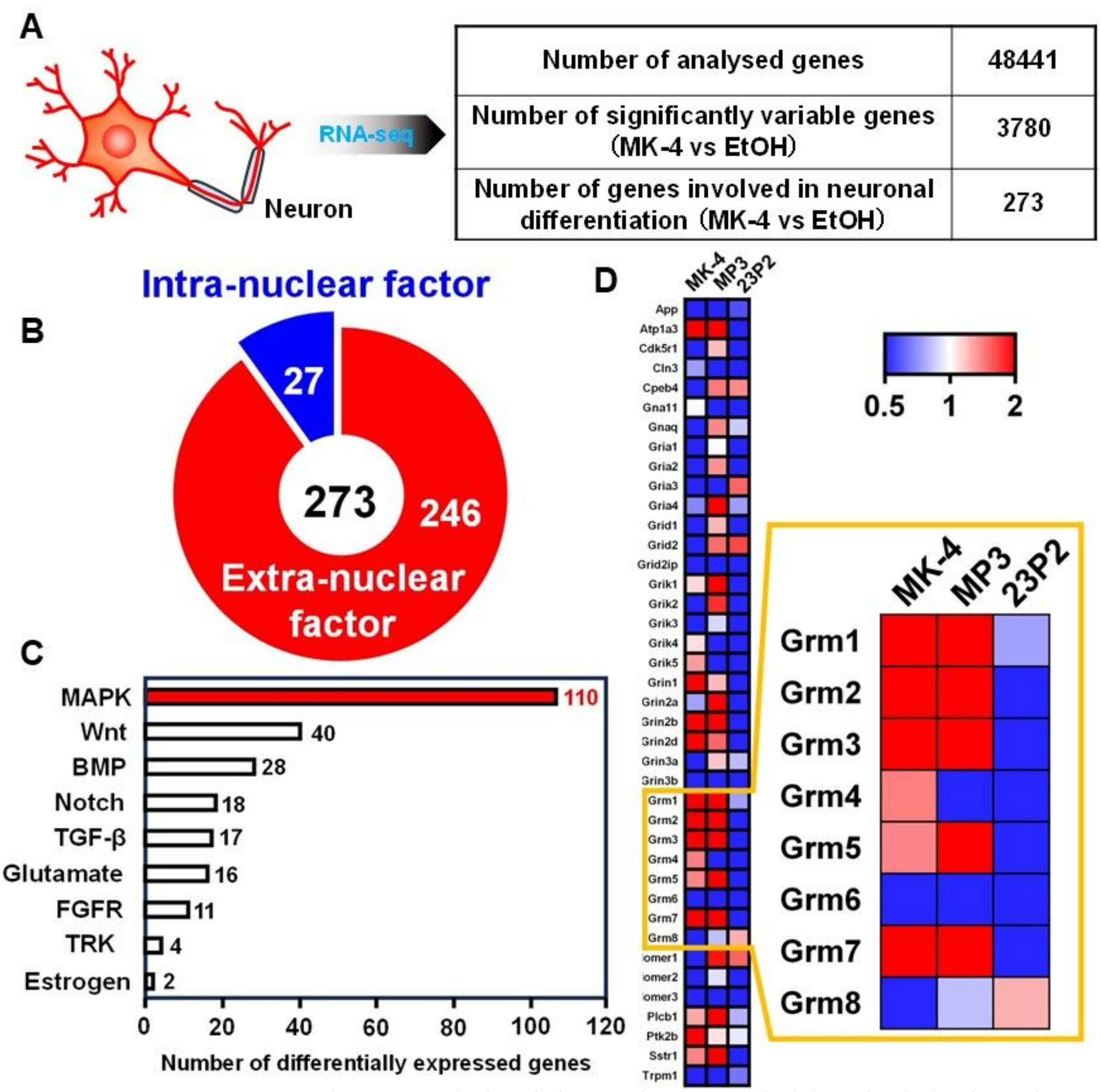

Supplementary Fig. 2. Transcriptome analysis of the mechanism underlying the induction of neuronal differentiation by MK-4 and vitamin K analogues . (A) Transcriptome analysis of MK-4–treated murine neural stem cells. Of 48,441 genes evaluated, 3780 were changed more than 2-fold in MK-4–treated cells, among which 273 genes were involved in neuronal differentiation. (B) Pie chart showing the proportion of extra- and intra-nuclear factors among the 273 identified genes involved in neuronal differentiation. (C) Of the 246 extra-nuclear factors involved in neuronal differentiation, 110 genes were found to be involved in the MAPK cascade. (D) Heat map of the most variable MAPK cascade genes involved in neuronal differentiation in MK-4–, MP3-, or 23P2-treated murine neural stem cells. Genes coding for metabotropic glutamate receptors were the most highly variable.

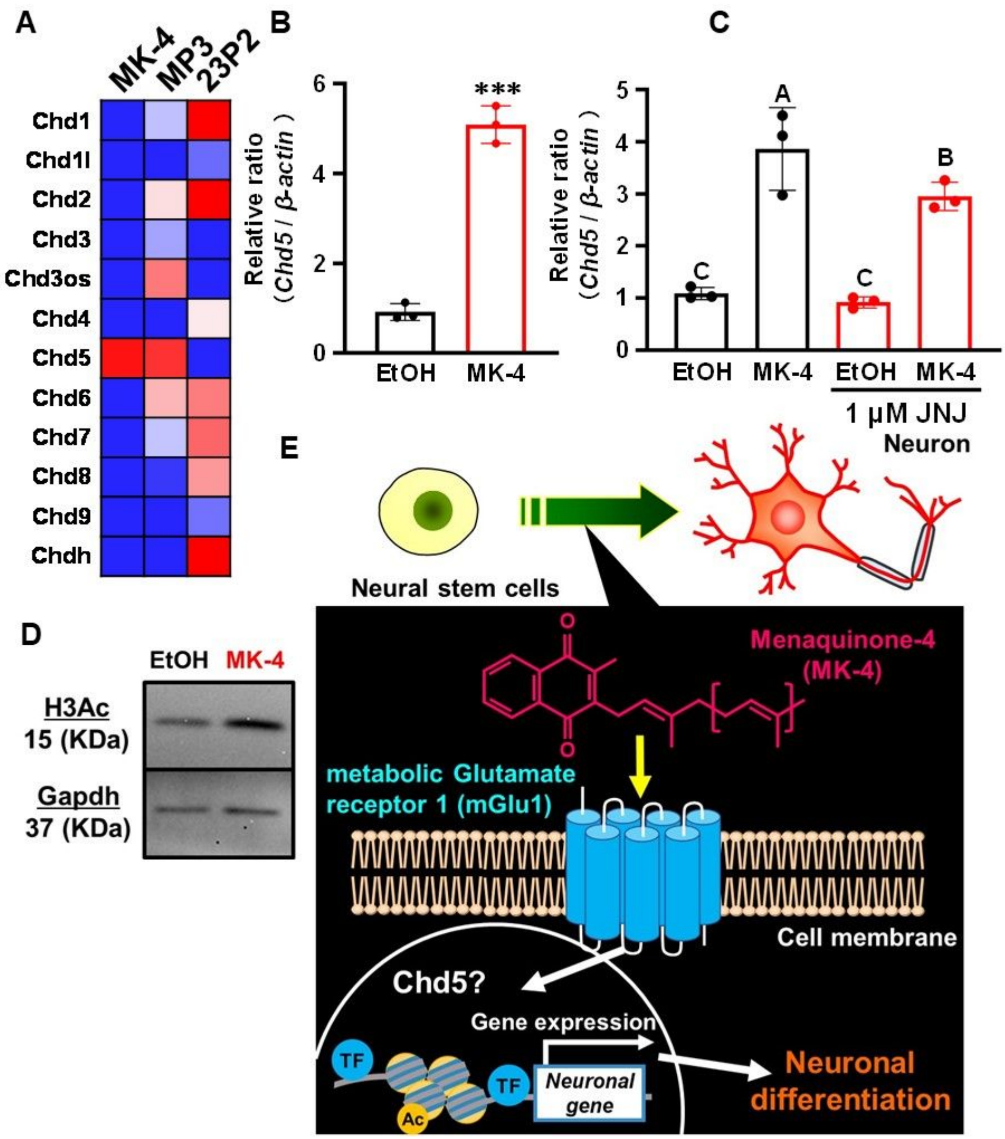

Supplementary Fig. 3. MK-4 may be transcriptionally activated (euchromatin state) in neural stem cells by Chd5 via metabotropic glutamate receptor 1 to activate histone acetylation and promote its activity to induce differentiation into neurons. (A) Heat map of Chd genes, the most highly variable gene family identified in our transcriptome analysis of 27 intranuclear factors and Chd genes heat map. Among them, *Chd5* gene expression varied greatly in MK-4–, MP3-, and 23P2-treated murine neural stem cells. (B) *Chd5* mRNA expression was significantly increased in MK-4–treated neural stem cells. Significant difference from EtOH : ***$p < 0.001$ (Student's *t*-test). (C) mRNA expression level of *Chd5* was significantly decreased in murine neural stem cells treated with a metabotropic glutamate receptor 1 inhibitor (JNJ16259685) and MK-4 compared to cells treated with MK-4 alone. Values not sharing a common letter in each group were significantly different ($n = 3$); $p < 0.05$ (Tukey–Kramer HSD test). (D) Western blot analysis showing that acetylation of histone H3 was activated in MK-4–treated neural stem cells. (E) Overview of the proposed mechanism by which MK-4 induces neural stem cell differentiation into neurons.

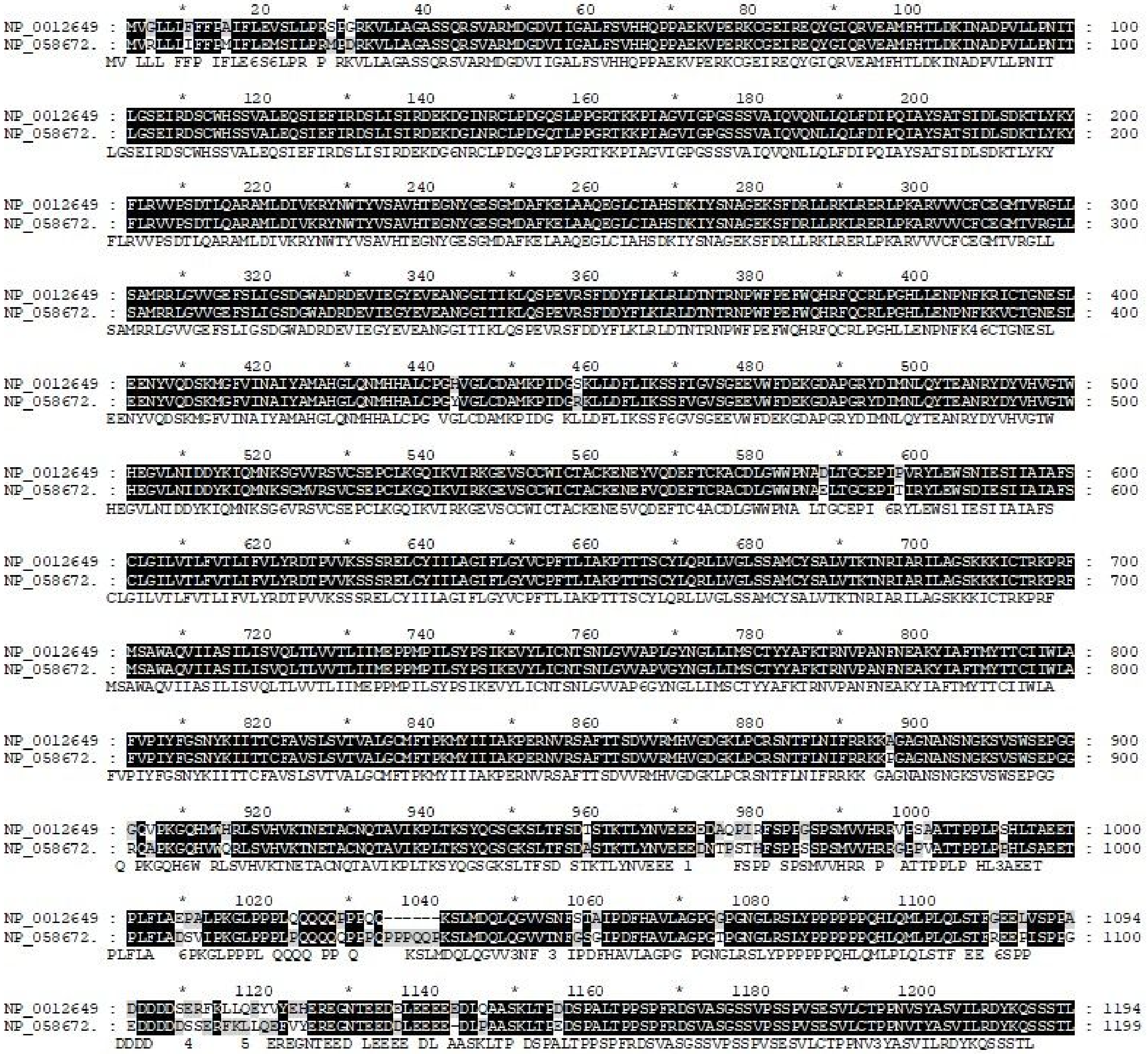

Supplementary Fig. 4. Human and mouse mGluR1 with 93.5% homology

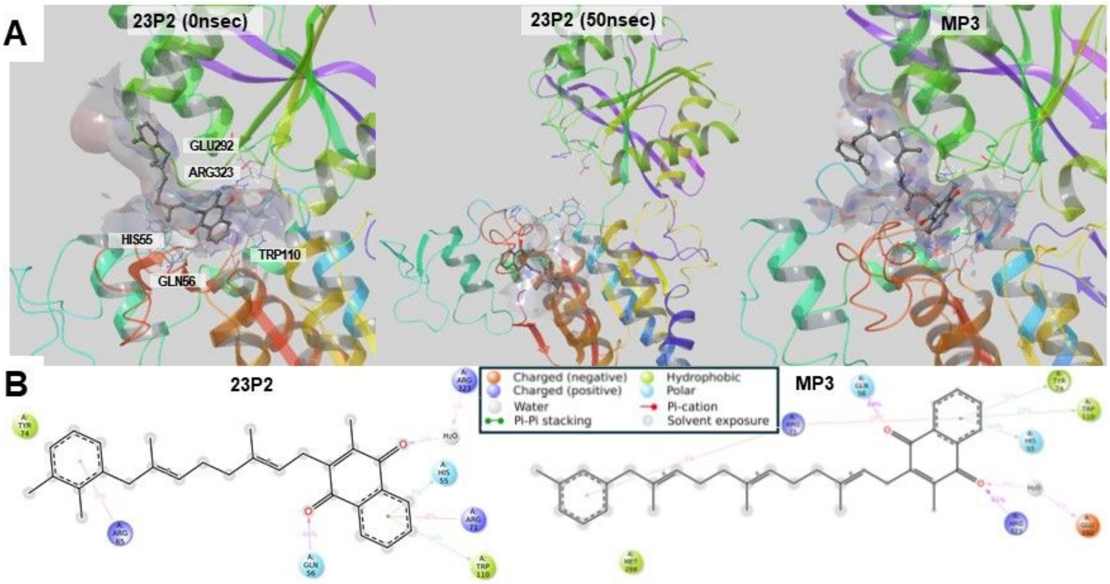

Supplementary Fig. 5. Structural analysis of the binding of human mGluR1 with the indicated vitamin K analogues. (A) Molecular docking analysis of the agonist binding site of mGluR1 and the binding poses of 23P2 and MP3. Binding surfaces are shown with an electrostatic potential overlay (blue, positive; red, negative). Docking results for 23P2 are shown at two time points: at 0.00 ns, 23P2 was bound to human mGluR1, but at 50.00 ns, it has been released from the ligand binding domain. (B) Schematic diagrams of the interactions between ligand atoms and protein residues. Interactions that occurred for more than 5.0% of the simulation time from 0.00 to 50.00 ns are shown.

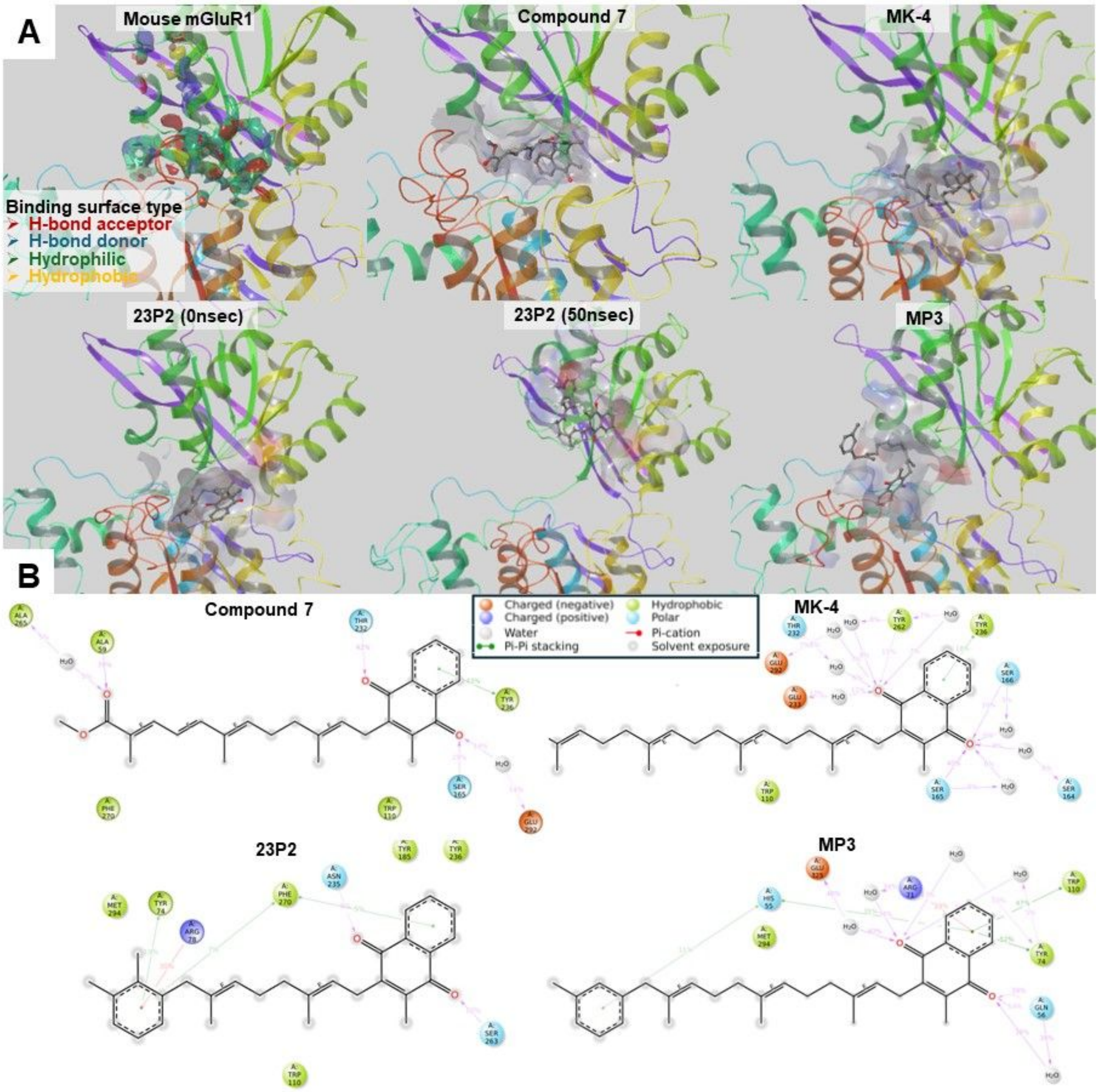

Supplementary Fig. 6. Structural analysis of the binding of murine mGluR1 with the indicated vitamin K analogues. (A) Molecular docking analysis of the agonist binding site of mGluR1 and the binding poses of vitamin K compounds. The binding surfaces are shown with an electrostatic potential overlay (blue, positive; red, negative). The docking results for 23P2 are shown at two timepoints: at 0.00 ns, 23P2 was bound to murine mGluR1, but at 50.00 ns it had been released from the ligand binding domain. (B) Schematic diagrams of the interactions between ligand atoms and mGluR1 residues. Interactions that occurred for more than 5.0% of the simulation time from 0.00 to 50.00 ns are shown.

A

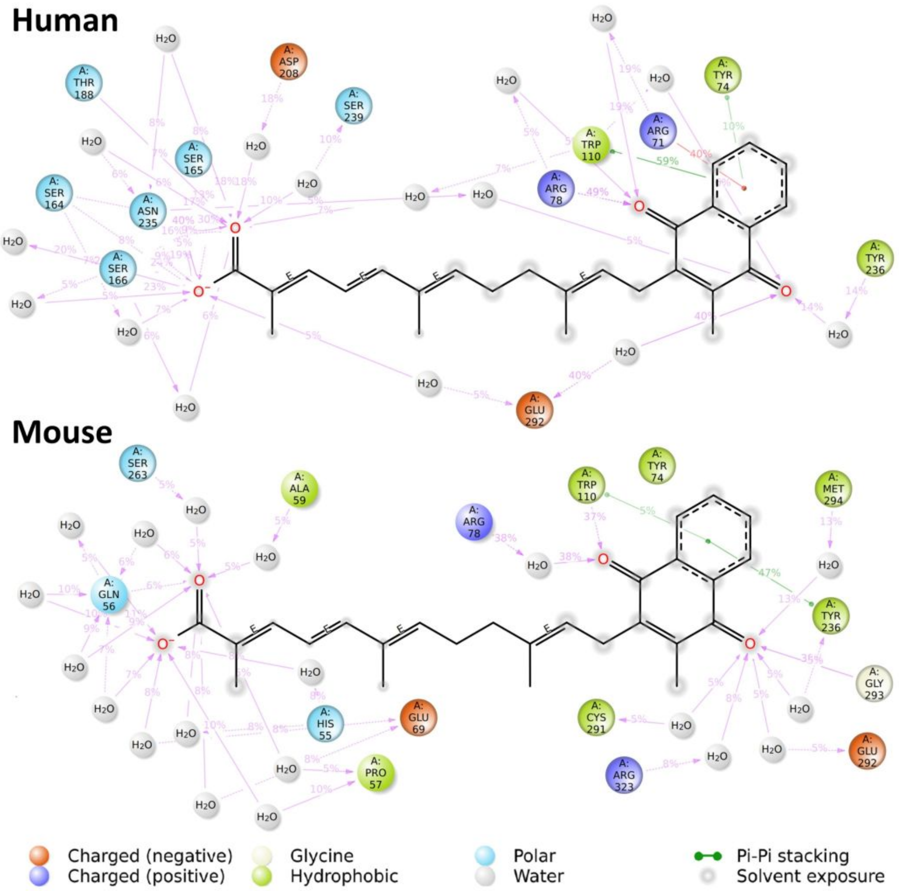

B

Compound	Docking score (kcal/mol)	e-model (kcal/mol)
MK-4	-6.03	-68.5
Compound **7** ($COOCH_3$ foam)	-8.38	-75.5
Compound **7'** (COOH foam)	-8.85	-96.7
Quisqualic acid (Positive Contorol)	-9.29	-79.5

Supplementary Fig. 7. Docking Simulation of Carboxylic Acid-Type Vitamin K Analog (Compound **7′**) with mGluR1. (A) Schematic diagrams of the interactions between Compound 7′ atoms and Human and Mouse mGluR1 residues. Interactions that occurred for more than 5.0% of the simulation time from 0.00 to 50.00 ns are shown. (B) Docking scores of vitamin K compounds against human mGluR1. Quisqualic acid was used as a positive control for mGluR1.A more negative docking score (kcal/mol) indicates stronger predicted binding affinity, while a more negative e-model score (kcal/mol) suggests a more favorable binding pose.
